# Gene-environment interaction study for BMI reveals interactions between genetic factors and physical activity, alcohol consumption and socioeconomic status

**DOI:** 10.1371/journal.pgen.1006977

**Published:** 2017-09-05

**Authors:** Mathias Rask-Andersen, Torgny Karlsson, Weronica E. Ek, Åsa Johansson

**Affiliations:** Department of Immunology, Genetics and Pathology, Science for Life Laboratory, Uppsala University, Uppsala, Sweden; Fred Hutchinson Cancer Research Center, UNITED STATES

## Abstract

Previous genome-wide association studies (GWAS) have identified hundreds of genetic loci to be associated with body mass index (BMI) and risk of obesity. Genetic effects can differ between individuals depending on lifestyle or environmental factors due to gene-environment interactions. In this study, we examine gene-environment interactions in 362,496 unrelated participants with Caucasian ancestry from the UK Biobank resource. A total of 94 BMI-associated SNPs, selected from a previous GWAS on BMI, were used to construct weighted genetic scores for BMI *(GS*_*BMI*_*)*. Linear regression modeling was used to estimate the effect of gene-environment interactions on BMI for 131 lifestyle factors related to: dietary habits, smoking and alcohol consumption, physical activity, socioeconomic status, mental health, sleeping patterns, as well as female-specific factors such as menopause and childbirth. In total, 15 lifestyle factors were observed to interact with *GS*_*BMI*_, of which alcohol intake frequency, usual walking pace, and Townsend deprivation index, a measure of socioeconomic status, were all highly significant (*p* = 1.45*10^−29^, *p* = 3.83*10^−26^, *p* = 4.66*10^−11^, respectively). Interestingly, the frequency of alcohol consumption, rather than the total weekly amount resulted in a significant interaction. The *FTO* locus was the strongest single locus interacting with any of the lifestyle factors. However, 13 significant interactions were also observed after omitting the *FTO* locus from the genetic score. Our analyses indicate that many lifestyle factors modify the genetic effects on BMI with some groups of individuals having more than double the effect of the genetic score. However, the underlying causal mechanisms of gene-environmental interactions are difficult to deduce from cross-sectional data alone and controlled experiments are required to fully characterise the causal factors.

## Author summary

Genome-wide association studies (GWAS) have identified hundreds of genes as being associated with body mass index (BMI). How these genetic effects are modulated by lifestyle factors has not been extensively investigated previously. Here we utilise data from approximately 360,000 participants from the UK Biobank, aged 40–69 years old, to identify interactions between genetic and lifestyle factors in relation to BMI. We investigated 131 lifestyle factors, of which 15 influence the genetic effects on BMI. The most significant factors were those related to physical activity, alcohol consumption, and socioeconomic status. For example, the effect of a genetic score for BMI was almost twice as high in participants who reported never drinking alcohol compared to every-day drinkers. Similarly, the effect of the genetic score for BMI was 2.5 times higher in participants who reported having a slow walking pace compared to participants who reported having a brisk walking pace. Our results show that many lifestyle factors influence the genetic effects, which suggests that changing our lifestyle may be a way to influence our genetic risk for obesity and other common human disorders.

## Introduction

Gene-environment interactions result from individuals responding differently to environmental stimuli depending on their genotype, or from genetic effects that vary between groups of individuals depending on their lifestyles. In humans, the most famous examples include skin color and risk of melanoma in response to ultra-violet rays, and phenylketonuria (PKU) in response to foods containing phenylalanine in individuals who carry mutations that lead to phenylalanine hydroxylase deficiency [1]. Gene-environmental interactions are likely to exist for complex human traits and identifying gene-environment interactions can potentially improve risk-assessment for disease and help unravel underlying biological pathways [1].

Obesity and being overweight are serious public health issues due to their strong associations with diseases such as cardiovascular disease, type 2 diabetes and cancer. In addition, their global prevalence has increased dramatically over the latter part of the 20^th^ century and up to the present day [2]. Body mass index (BMI) is a standardised measure of human body size that is calculated from weight and height. Twin studies have demonstrated a heritable component of BMI and genome-wide association studies (GWAS) have shown that BMI is influenced by hundreds of common genetic variants [3–5]. Recently, a GWAS for BMI on 339,224 individuals, reported 97 genetic loci to be associated with variation in BMI [4]. However, only a few studies have investigated the effect of gene-environment interactions on BMI. Previous studies have reported physical activity to attenuate the effect of genetic factors on BMI, including the effects of genetic variants within the *FTO* locus [6–9]. Identification of gene-environment interactions for complex human traits poses several challenges. For instance, most GWAS of complex traits have been performed by large-scale meta-analyses of multiple cohorts, which complicate a harmonised collection of lifestyle and environmental data. Also, the effects of genetic variants identified through GWAS are generally small [4], and differences in the effects of genetic variants between groups exposed to different lifestyle factors may be difficult to detect in smaller cohorts due to lack of statistical power.

Initiatives such as the UK Biobank provide a unique opportunity to study interactions between genetic and lifestyle factors. Data collection in UK Biobank has been performed in a standardised manner and data include a large number of lifestyle and environmental factors collected from approximately half a million UK citizens, as well as comprehensive, genome-wide genotyping [10]. Recent studies on the UK Biobank found that the effect of the *FTO* locus variant, rs1421085, interacts with several lifestyle risk factors such as alcohol consumption, sleep patterns, diet and physical activity [9]. Another recent study on the UK Biobank determined that the effect of a genetic risk score for BMI is modified by socioeconomic status [11].

Here, we study the effects of gene-environment interactions on BMI, by analysing 131 lifestyle factors assessed by touchscreen questionnaires. These factors include diet, smoking, alcohol consumption habits, physical activity, socioeconomic status, mental health, sleep, general health as well as factors that are specific to females such as number of live births. For the purpose of our analyses, we constructed a genetic score for BMI (*GS*_*BMI*_) which was composed of 94 single-nucleotide polymorphisms (SNPs) that have previously been associated with BMI in a GWAS [4].

## Materials and methods

### UK Biobank cohort

We utilised data from the UK Biobank Resource (http://www.ukbiobank.ac.uk/about-biobank-uk/) [10] for all analyses. UK Biobank has recruited more than 500,000 individuals aged 40–69 from the United Kingdom during the years 2006–2010. Participants underwent standardised measurements of anthropomorphic traits, and additionally provided biological samples and detailed information about themselves via touchscreen questionnaires. Genotyping had been performed using two custom-designed UK Biobank Axiom Arrays with 820,967 and 807,411 SNPs respectively (BiLEVE and Axiom). Genotypes that were not directly assayed had been imputed [12] using a combined set consisting of the UK10K [13] haplotype reference panel and the 1000 Genomes phase 3 reference panel [14]. We utilized the initial release of genotype data (data accessed January 2016) as a discovery cohort, and the remaining participants with genotype data available in the second release as a replication cohort (data accessed July 2017). In the initial release, data were available for 73,355,667 SNPs in 152,249 UK Biobank participants. To identify related individuals, we used information provided by UK Biobank (Data-Field: 22011—Genetic relatedness pairing). Briefly, kinship coefficients had been calculated for each pair of participants in the cohort using the genetic data and pairs of related individuals had been identified (at least 3^rd^ degree relatives = kinship coefficient > 0.044). In addition, only people who self-identified as white British (Data-Field 21000) and that were classified as Caucasians based on the genetic principal components (Data-Field 22006) were included. After filtering, 116,138 individuals remained for the analysis in the discovery cohort. The same filtering was applied to the replication cohort, leaving 246,358 participants for the replication.

### Ethics

All participants had provided signed consent to participate in UK Biobank [15]. UK Biobank has been given ethical approval to collect participant data by the North West Multicentre Research Ethics Committee, which covers the UK; the National Information Governance Board for Health & Social Care, which covers England and Wales, and the Community Health Index Advisory Group, which covers Scotland. UK Biobank possesses a generic Research Tissue Bank approval granted by the National Research Ethics Service (http://www.hra.nhs.uk/), which lets applicants conduct research on UK Biobank data without obtaining separate ethical approvals. Access to UK Biobank genetic and phenotypic data was granted under application no. 15152: “Interaction between diet, food preference and lifestyle with genetic factors influencing body mass, body adiposity and obesity”. Written consent was obtained from all participants.

### Phenotypic measurements

Participants’ weights were assessed by a variety of means during the initial UK Biobank assessment centre visit. For weight, we utilised data-field 21002, which is an amalgate of all weight values into a single item. Standing height was measured on a SECA 240 Height Measure. BMI was constructed from height and weight measurements during participants’ initial visit to assessment centers. For most analyses, BMI was transformed using rank-based inverse normal transformation.

Lifestyle factors have primarily been collected via self-report touchscreen questionnaire. All lifestyle variables that had been assessed in more than 20,000 of the participants were included for analyses. This resulted in 131 quantitative, ordinal and categorical measurements of lifestyle factors representing dietary habits, general health, sleep, smoking and alcohol consumption, physical activity, mental health and socioeconomic status (S1 Table).

We aimed to use linear regression models to test for interaction between lifestyle factors and genetic factors on BMI. To this end, “Prefer not to answer” and “I don’t know” were set to “missing” in our analyses. We removed the 99^th^ percentile of quantitative phenotypic variables, such as, for example: “Average weekly red wine intake” in number of glasses, and “Duration of moderate physical activity” in minutes, to reduce the effect of outliers. We analysed ordinal phenotypic data as quantitative variables. For example, data-field 1558—frequency of alcohol intake: which is coded as: 1 = “Daily or almost daily”, 2 = “Three or four times a week”, 3 = “Once or twice a week”, 4 = “One to three times a month”, 5 = “special occasions only”, 6 = “Never”.

Data field 20126 represents bipolar and major depression status among participants. This variable was derived from self-report questionnaire data [16]. Very few patients were assessed to have bipolar disorder type I and II (n = 808 & 807 respectively) and these were designated missing. Severity of depression was assessed as 0 = “No depression”, 1 = “Single probable major depression episode”, 2 = “Probable recurrent major depression (moderate)”, 3 = “Probable recurrent major depression (severe)”. Categories 1 to 3 were combined and this field was recoded as “No depression” = 0, and “Probable depression” = 1. ‘Had menopause’ (Data-field 2724) was recoded to better represent linearity: participants who were uncertain due to having undergone a hysterectomy were designated “missing”. Data field 680: “Own or rent accommodation lived in”, was recoded to better represent linearity with regard to socioeconomic status: 1 = “Own outright”, 2 = “Own with mortgage”, 3 = “Rent, from local authority”, 4 = “Rent, from private landlord or letting agency”. Categories 5: “Pay rent and part mortgage (shared ownership)” and 6: “Live in accommodation rent free”, were set to missing due to the low number of participants in these groups (N = 303 and N = 735, respectively). Self-reported drinking habits were converted to amounts in ml alcohol per week using standard sizes for serving and percentages: red wine—125 ml per glass, 13.5% alcohol; white wine– 125 ml per glass, 12.0% alcohol; beer and cider– 570 ml per pint, 5.5% alcohol; spirits– 30 ml per measure, 41.5% alcohol; fortified wine– 58 ml per glass, 19% alcohol. Amounts of exercise per week for specific exercise-types, e.g., “10+ minute walks”, “walking for pleasure”, “moderate physical activity”, and “vigorous physical activity”; were calculated by multiplying the exercise frequency per week with the duration of activity in minutes.

### Replication of BMI SNPs and calculations of genetic scores for BMI (GS_BMI_)

Genotype data for 97 SNPs that have previously been identified to be associated with BMI [4] were considered for our analyses (S2 Table). One SNP, rs2033529 was not part of the UK biobank dataset and was replaced by another linked SNP rs751414 (r^2^ = 0.99, D’ = 1). Three SNPs were removed due to deviation form Hardy Weinberg equilibrium, which left 94 SNPs for the analyses. Since many of these variants have not been replicated in an independent cohort, we first tested for association in the initial release of genotype data from the UK Biobank cohort. This was done using linear regression models with BMI as a response variable. BMI was first transformed using a rank-based inverse normal transformation similar to the discovery study [4]. The UK Biobank participants were genotyped on two different genotyping arrays: (BiLEVE and Axiom), and a variable to adjust for this was included as a covariate in addition to sex, age, age^2^ and the first 15 genetic principal components (PCs). Some of the SNPs were identified as being associated with BMI in females or males separately in the previous study [4]. We therefore also tested for association in males and females separately and compared whether there was a significant difference in the estimates between males and females.

Genotype data was used in dosage format, where SNP genotypes were represented by the number of copies of the effective allele. To calculate *GS*_*BMI*_, regression coefficients (*β-*estimates) were retrieved from the GIANT consortium meta-analysis for BMI for the European populations with males and females combined [4]. Weighted *GS*_*BMI*_ were then calculated for each individual by multiplying the number of effective alleles for each of the 94 SNPs (all SNPs in HWE in UK biobank) with the respective *β-*estimates (i.e., ${\hat{\beta }}_{SNP,i},\;i=1,\ldots ,94$) and calculating the sum over all SNPs (S2 Table):

$${{GS}_{BMI}=\sum_{i=1}^{94}\beta }_{SNP,i}*\;{SNP}_{i}$$ (1)

### Statistical analysis to identify gene × environment interactions

Linear regression modeling was used to estimate the effect of gene-environment interaction (*GS*_*BMI*_ × *E*) on BMI, for 131 lifestyle factors (*E*) separately. In addition to the *GS*_*BMI*_ *× E* interaction term, each of the 131 models was adjusted for covariates: age, age^2^, sex, PCs, and genotyping array (batch). Interaction terms for *GS*_*BMI*_ with age, age^2^, and sex as well as interaction terms for the lifestyle factor with age, age^2^, and sex were also included in order to properly control for possible confounding effects of these interactions, in accordance with previously published recommendations [17], such that:

$${BMI}_{}=\beta_{0}+\beta_{1}{GS}_{BMI}+\beta_{2}E+\beta_{3}{GS}_{BMI}\times E+\beta_{4}Age{+\beta }_{5}{Age}^{2}{+\beta }_{6}Sex{+\beta_{7}{GS}_{BMI}\times Age{+\beta }_{8}{GS}_{BMI}\times {Age}^{2}{+\beta }_{9}{GS}_{BMI}\times Sex+\beta_{10}E\times Age{+\beta }_{11}{E\times Age}^{2}{+\beta }_{12}E\times Sex+\beta }_{13}Batch+\sum_{i=1}^{15}\beta_{PC,i}{PC}_{i}+\varepsilon$$ (2)

We assume that the error term *ε ~ NID(0*, *σ*^*2*^*)*. The models also included 15 principal components (*PCs*) to account for effects of population stratification in UK Biobank. In the primary analyses, models for each of the 131 lifestyle factors were analysed separately. The aim of this study was to investigate the effect of the interaction term *GS*_*BMI*_*×E* on BMI. For this purpose, we focused our attention on the estimate of the coefficient *β*_*3*_ in (2), and more specifically whether this estimate significantly deviated from zero. The null hypothesis *H*_*0*_: *β*_*3*_ *= 0* was either accepted or rejected, depending on the outcome of a two-sided marginal student’s t-test, which in this case (i.e., one degree-of-freedom difference between the nested models and normal regularity conditions) is equivalent to a likelihood-ratio test of the hypothesis *H*_*0*_: *β*_*3*_ *= 0*. P-values lower than the significance level *α =* 0.05/131 *≈* 3.82*10^−4^ were considered significant to account for the family-wise error rate using the Bonferroni method. Interaction effects that were considered significant in the discovery cohort were then tested in the replication cohort using the same covariates as well as 15 PCs. Calculations were performed in R version 3.3.0 [18] using the “lm” function included in the stats package.

In order to visualise and make it easier to interpret the significant interactions, we also estimated the effect of *GS*_*BMI*_ and of individual SNPs on BMI in different subgroups with regards to lifestyles, e.g., the genetic effect in participants with different frequencies of alcohol consumption. In these analyses, linear regression was performed in the subgroups using the same covariates as above, but with untransformed BMI values as a response variable, for easier interpretability of the regression coefficients (presented in kg/m^2^). Here the differences in effect between the subgroups reflect the interaction term from the previous analyses. Interactions were visualised with bar graphs using the ggplot2 package in R. We also used the plot3D R package to visualise interactions in 3D-plots.

### Sensitivity analyses

Genetic variants within intron one of *FTO* have consistently been shown to be the strongest genetic factors associated with BMI [3–5,19,20]. We therefore constructed a genetic score that excluded the *FTO*-linked SNP rs1558902 (*GS*_*BMI*_*’*), and performed linear regression modeling in exactly the same manner as previously. We also performed additional analyses to assess how individual SNPs interacted with lifestyle factors. SNP-interactions that were considered significant in the discovery cohort were further tested in the replication cohort. We also performed sensitivity analyses by including TDI and its interactions with age, age^2^, sex, *GS*_*BMI*_, as well as each of the lifestyle factors in the model, in addition to the variables described in Eq (2).

For calculating *GS*_*BMI*_, we used the regression coefficients from the GIANT consortium. However, in a discovery GWAS, the regression coefficients are often overestimated, which will introduce a bias in the *GS*_*BMI*_. For this reason, we also performed additional analyses using the regression coefficients estimated in UK Biobank, for the sake of comparison.

### Stepwise linear regression

To determine which of the interacting lifestyle factors had an independent contribution in the regression model for BMI, we performed stepwise linear regression (SLR) using the ‘step’ function included in the ‘stats’ package in R [18]. This function uses the Akaike information criterion (AIC) to select variables for the model. A base-model for BMI was constructed that included *GS*_*BMI*_, age, age^2^, sex, a batch variable to control for the two genotyping platforms used in UK Biobank, as well as 15 principal components. Variables that were significant after replication were included in SLR. SLR was performed using ‘both’ directions so that variables were either added or dropped depending on how they improved AIC. The process is repeated until no improvement in AIC can be made. Individuals with any missing data were excluded from the analyses, and in order to maintain a large sample size for the analyses, we performed SLR on a combined set of the discovery and replication cohorts. Individuals with missing data in any of the tested factors were excluded before running SLR, which resulted in 290,441 participants remaining after filtering. All secondary interactions between variables were included in the analysis to control for potential confounding, in accordance with recommendations by Keller [17].

## Results

**Fig 1:**
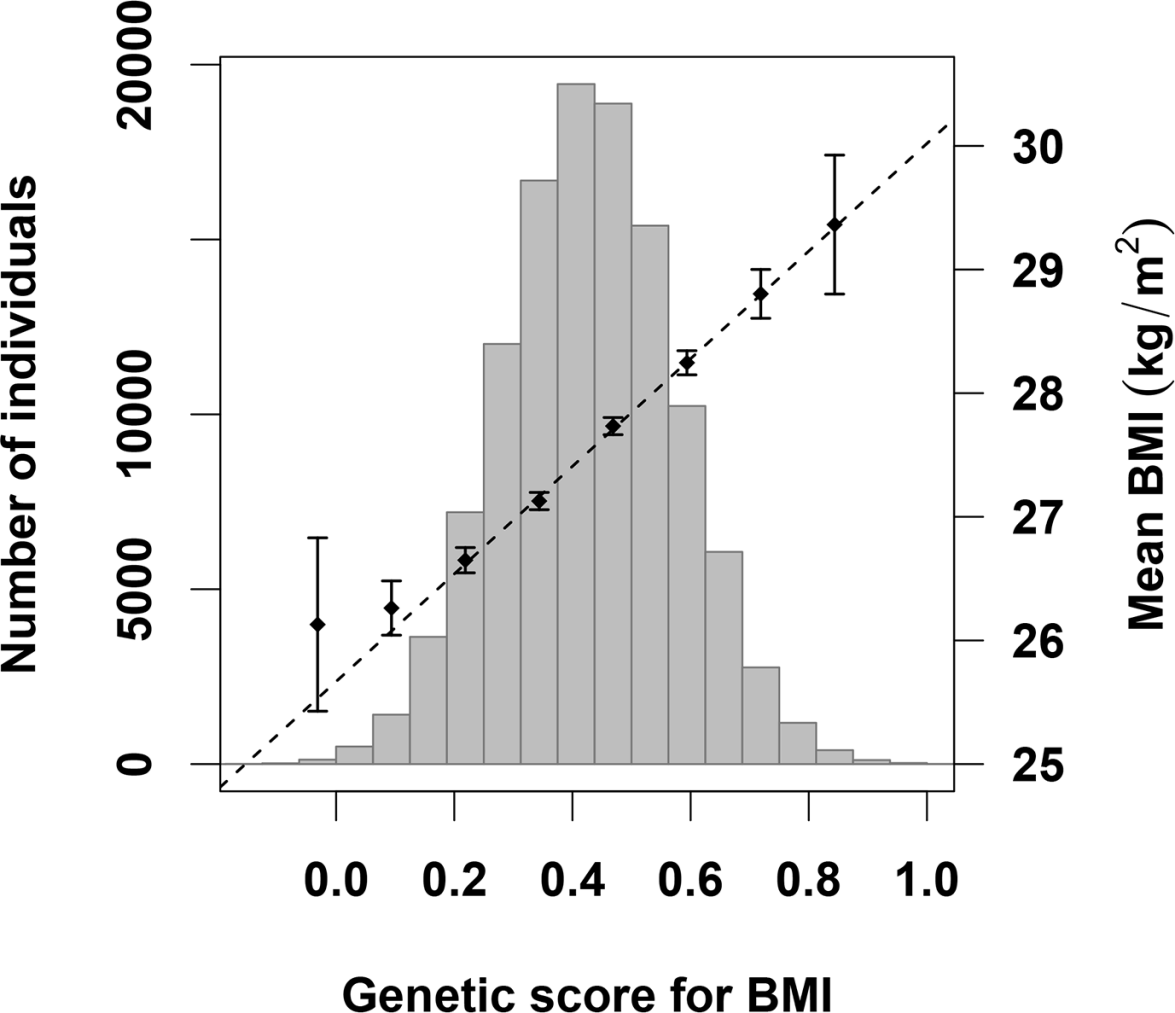
Distribution of weighted genetic scores for BMI (*GS*_*BMI*_) in participants from the UK Biobank (left y-axis). The average BMI in kg/m^2^ (right y-axis) for participants in each bin of the histogram is plotted as black diamonds ± 95% confidence interval. The dotted line represents the regression of BMI across *GS*_*BMI*_.

Genotype and phenotype data were available for 487,409 participants, of which 362,496 passed the QC and were included in the analyses (116,138 in the discovery and 246,358 in the replication). Basic characteristics are provided in Table 1. The distribution of BMI was slightly skewed (S2 Fig), and we therefore applied a rank-based inverse normal transformation of BMI prior to the analyses (Fig 1), in agreement with previous GWAS on BMI [4]. Out of the 94 SNPs that were in HWE in UK Biobank, the association with BMI replicated for 63 of them (S1 Table). However, the effect sizes for the 94 SNPs were consistent with previous data from the GIANT consortium (S1 Fig) [4]. We therefore included all 94 SNPs in the construction of *GS*_*BMI*_. No differences in effect size between males and females were observed for any of the 94 SNPs (S3 Table) and we therefore used the same regression coefficients for calculating the *GS*_*BMI*_ in males and females. *GS*_*BMI*_ was calculated so that a one-unit increase in *GS*_*BMI*_ was associated with a one-standard deviation increase in BMI, which in our data equals 4.83 kg/m^2^ (Fig 1). *GS*_*BMI*_ explained 1.85% of the variation in BMI in the studied subset of the UK Biobank participants.

**Table 1:** Basic characteristics of UK Biobank participants included for this study, stratified by weight status.

(Discovery cohort / replication cohort)	Underweight BMI < 20		Normal weight (BMI = 20–25)	Overweight (BMI = 25–30)	Obese BMI > 30	
Number of participants	2,611 / 5,646		34,769 / 76,489	49,432 / 105,005	29,011 / 58,386	
Males (%)	22.2 / 19.7		37.2 / 35.6	54.4 / 53.9	49.8 / 47.9	
Age at assessment centre (years)	55.4 ± 8.3 / 55.1 ± 8.3		56.5 ± 8.2 / 56.8 ± 8.0	57.6 ± 8.0 / 57.4 ± 7.96	57.4 ± 7.8 / 57.2 ± 7.8	
Height (cm)	167.0 ± 8.4 / 167.0 ± 8.3		168.1 ± 8.9 / 168.2 ± 8.9	170 ± 9.3 / 169.7 ± 9.4	168.4 ± 9.4 / 168.2 ± 9.5	
Weight (kg)	53.1 ± 6 / 53.0 ± 5.9		65.5 ± 8.1 / 65.5 ± 8.2	78.8 ± 9.6 / 78.8 ± 9.6	96.5 ± 14.4 / 96.0 ± 14.3	
BMI (kg/m2)	19 ± 0.9 / 19.0 ± 0.9		23.1 ± 1.3 / 23.1 ± 1.31	27.3 ± 1.4 / 27.3 ± 1.4	34 ± 3.9 / 33.9 ± 3.8	
**Smoking status (%) (never / previous / current)**						
Never	58.7 / 61.8		59.9 / 59.5	52.1 / 53.7	48.3 / 51.5	
Previous	19.0 / 24.1		27.8 / 31.2	36.3 / 37.3	40.7 / 40.0	
Current	22.3 / 14.1		13.0 / 9.3	11.6 / 8.9	11.0 / 8.5	
Moderate physical activity (days per week)	4.0 ± 2.4 / 3.7 ± 2.5		3.9 ± 2.3 / 3.4 ± 2.5	3.6 ± 2.3 / 3.42 ± 2.46	3.3 ± 2.4 / 3.0 ± 2.5	
**Alcohol consumption frequency (%)**						
Daily or almost daily	26.1 / 23.1		24.8 / 23.6	24.0 / 22.4	18.1 / 16.1	
Three or four times a week	23.1 / 23.2		26.6 /26.1	26.2 / 25.8	21.8 / 20.0	
Once or twice a week	24.1 / 22.2		27.5 / 25.8	28.3 / 26.8	28.9 / 27.2	
One to three times a month	11.9 / 10.3		11.0 / 10.1	11.3 / 10.3	14.4 / 13.8	
Special occasions only	14.6 / 11.4		10.0 / 8.6	10.1 / 9.1	16.7 / 14.6	
Never	12.5 / 9.8		6.6 / 5.8	6.3 / 5.6	9.5 / 8.3	
Townsend deprivation index	-1.0 ± 3.3 / -1.3 ± 3.1		-1.7 ± 2.9 / -1.8 ± 2.8	-1.6 ± 2.9 / -1.7 ± 2.8	-1.05 ± 3.14 / -1.14 ± 3.09	

Values are presented as average ± standard deviations, total number or percentages (%).

Linear regression modeling in the initial release of genotype data from the UK Biobank cohort (discovery cohort) revealed *GS*_*BMI*_ to interact with 19 lifestyle factors related to physical activity, alcohol consumption, smoking, socioeconomic status, sleep, mental health and number of live births (Table 2, S4–S9 Tables), when we applied Bonferroni adjustment for multiple testing (*p* < 3.8*10^−4^). Of these, interactions with 15 factors were replicated using the second release of genotype data from the UK Biobank cohort (Table 3). If we instead apply the false discovery rate to adjust for multiple testing in the discovery cohort, 38 interacting lifestyle factors were identified. The additional FDR-significant factors fall into the same categories as previously mentioned with the addition of variables related to variation in diet, and intake of bread, processed meat, and salad or raw vegetables (S4–S9 Tables).

**Table 2:** Factors observed to interact with *GS*_*BMI*_ after adjusting for multiple by using Bonferroni method.

ID	Name	N	*E*		*GS*_*BMI*_ *× E*		*GS*_*BMI*_*' × E*	
			*p1*	*β1*	*p2*	*β2*	*p3*	*β3*
924	Usual walking pace	115,525	<2.2*10^−308^	-0.49	1.10*10^−19^	-0.25	1.04*10^−14^	-0.23
1558	Alcohol intake frequency.	116,063	<2.2*10^−308^	8.35*10^−2^	1.87*10^−16^	9.92*10^−2^	4.37*10^−13^	9.34*10^−2^
189	Townsend deprivation index at recruitment	115,988	1.32*10^−121^	2.31*10^−2^	2.38*10^−10^	3.80*10^−2^	3.57*10^−8^	3.52*10^−2^
884	Number of days/week of moderate physical activity 10+ minutes	110,619	3.43*10^−290^	-4.61*10^−2^	1.46*10^−7^	-4.07*10^−2^	1.65*10^−6^	-3.96*10^−2^
1960	Fed-up feelings	113,941	2.99*10^−203^	0.18	4.43*10^−7^	0.19	2.39*10^−6^	0.19
738	Average total household income before tax	100,421	7.50*10^−142^	-7.21*10^−2^	4.60*10^−7^	-8.78*10^−2^	5.85*10^−5^	-7.47*10^−2^
2080	Frequency of tiredness / lethargy in last 2 weeks	112,854	<2.2*10^−308^	0.15	5.68*10^−7^	0.11	9.31*10^−6^	0.10
1070	Time spent watching television (TV)	110,003	<2.2*10^−308^	0.13	9.11*10^−7^	5.96*10^−2^	9.15*10^−6^	5.74*10^−2^
728	Number of vehicles in household	115,444	0.94	-2.62*10^−4^	1.02*10^−6^	-0.10	4.29*10^−5^	-9.23*10^−2^
943	Frequency of stair climbing in last 4 weeks	115,244	<2.2*10^−308^	-9.46*10^−2^	3.67*10^−6^	-6.40*10^−2^	2.34*10^−4^	-5.44*10^−2^
2050	Frequency of depressed mood in last 2 weeks	111,366	1.49*10^−65^	8.38*10^−2^	1.14*10^−5^	0.13	3.59*10^−5^	0.13
709	Number in household	115,505	4.55*10^−5^	-1.03*10^−2^	1.65*10^−5^	-6.71*10^−2^	7.17*10^−5^	-6.81*10^−2^
864	Number of days/week walked 10+ minutes	114,174	1.35*10^−256^	-5.16*10^−2^	2.68*10^−5^	-3.92*10^−2^	1.02*10^−4^	-3.88*10^−2^
1190	Nap during day	116,098	4.14*10^−289^	0.18	3.96*10^−5^	0.13	1.76*10^−4^	0.12
137	Number of treatments/ medications taken	116,127	<2.2*10^−308^	8.17*10^−2^	7.02*10^−5^	2.63*10^−2^	1.18*10^−3^	2.29*10^−2^
2734	Number of live births	61,087	1.30*10^−22^	3.85*10^−2^	9.78*10^−5^	-9.41*10^−2^	2.55*10^−4^	-9.41*10^−2^
20116	Smoking status	115,827	2.83*10^−18^	3.68*10^−2^	1.58*10^−4^	9.73*10^−2^	2.36*10^−3^	8.36*10^−2^
1568	Average weekly red wine intake	81,566	3.36*10^−9^	-4.12*10^−3^	2.37*10^−4^	-1.58*10^−2^	1.20*10^−4^	-1.77*10^−2^
904	Number of days/week of vigorous physical activity 10+ minutes	110,534	1.80*10^−255^	-5.22*10^−2^	3.09*10^−4^	-3.38*10^−2^	3.06*10^−4^	-3.61*10^−2^

*p*-values < 3.8*10^−4^ (0.05/131) were considered statistically significant *GS*_*BMI*_*−*Genetic score for BMI composed of the effects of 94 BMI-associated SNPs; *β1*—Estimates of the effect of environmental factors, *E*, on BMI (rank-transformed); *p1*—the corresponding p-values; *β2*—Estimates of the interaction term for *GS*_*BMI*_ *x E* with corresponding p-values (*p2*). *GS*_*BMI*_*'*—genetic score for BMI excluding the *FTO* SNP rs1558902 with corresponding estimates (*β3)* and p-values *(p3)*.

**Table 3:** Replication of interactions observed in the discovery cohort.

ID	Name	N	*GS*_*BMI*_ *× E* replication		*GS*_*BMI*_*’ × E* replication	
			*p1*	*β1*	*p2*	*β2*
924	Usual walking pace	244,780	3.83*10^−26^	-1.75*10^−1^	3.32*10^−26^	-1.88*10^−1^
1558	Alcohol intake frequency.	245,491	1.45*10^−29^	9.42*10^−2^	5.64*10^−24^	9.00*10^−2^
189	Townsend deprivation index (TDI) at recruitment	245,204	4.66*10^−11^	2.76*10^−2^	4.93*10^−10^	2.79*10^−2^
884	Number of days/week of moderate physical activity 10+ minutes	245,491	1.44*10^−7^	-2.58*10^−2^	1.08*10^−4^	-2.03*10^−2^
1960	Fed-up feelings	245,491	4.25*10^−5^	9.36*10^−2^	5.99*10^−4^	8.37*10^−2^
738	Average total household income before tax	244,709	2.79*10^−3^	-1.85*10^−2^	2.00*10^−2^	-1.54*10^−2^
2080	Frequency of tiredness / lethargy in last 2 weeks	245,491	1.51*10^−10^	8.38*10^−2^	1.97*10^−8^	7.83*10^−2^
1070	Time spent watching television (TV)	245,491	1.61*10^−7^	2.06*10^−2^	1.80*10^−5^	1.79*10^−2^
728	Number of vehicles in household	244,961	6.02*10^−6^	-6.14*10^−2^	1.75*10^−5^	-6.24*10^−2^
943	Frequency of stair climbing in last 4 weeks	244,530	3.56*10^−13^	-6.74*10^−2^	2.42*10^−11^	-6.61*10^−2^
2050	Frequency of depressed mood in last 2 weeks	245,491	1.95*10^−3^	4.88*10^−2^	2.73*10^−3^	5.05*10^−2^
709	Number in household	244,961	6.11*10^−1^	-5.25*10^−3^	9.12*10^−1^	1.21*10^−3^
864	Number of days/week walked 10+ minutes	245,491	8.92*10^−14^	-4.37*10^−2^	6.36*10^−12^	-4.29*10^−2^
1190	Nap during day	245,491	2.34*10^−7^	1.08*10^−1^	4.46*10^−5^	9.06*10^−2^
137	Number of treatments/ medications taken	245,483	4.39*10^−15^	3.68*10^−2^	8.79*10^−12^	3.41*10^−2^
2734	Number of live births	133,475	4.04*10^−1^	-1.32*10^−2^	8.61*10^−1^	-2.98*10^−3^
20116	Smoking status	245,491	2.56*10^−3^	5.42*10^−2^	4.69*10^−2^	3.80*10^−2^
1568	Average weekly red wine intake	177,221	1.81*10^−1^	-3.32*10^−3^	2.93*10^−1^	-2.79*10^−3^
904	Number of days/week of vigorous physical activity 10+ minutes	245,491	1.48*10^−12^	-4.33*10^−2^	4.43*10^−8^	-3.59*10^−2^

Replication was performed for both *GS*_*BMI*_ *(p1 and β1)* and *GS*_*BMI*_*’ (p2 and β2)*. Bonferroni correction was used to adjust for multiple testing and *p-*values < 2.6*10^−3^ (0.05/19) were considered significant.

**Fig 2:**
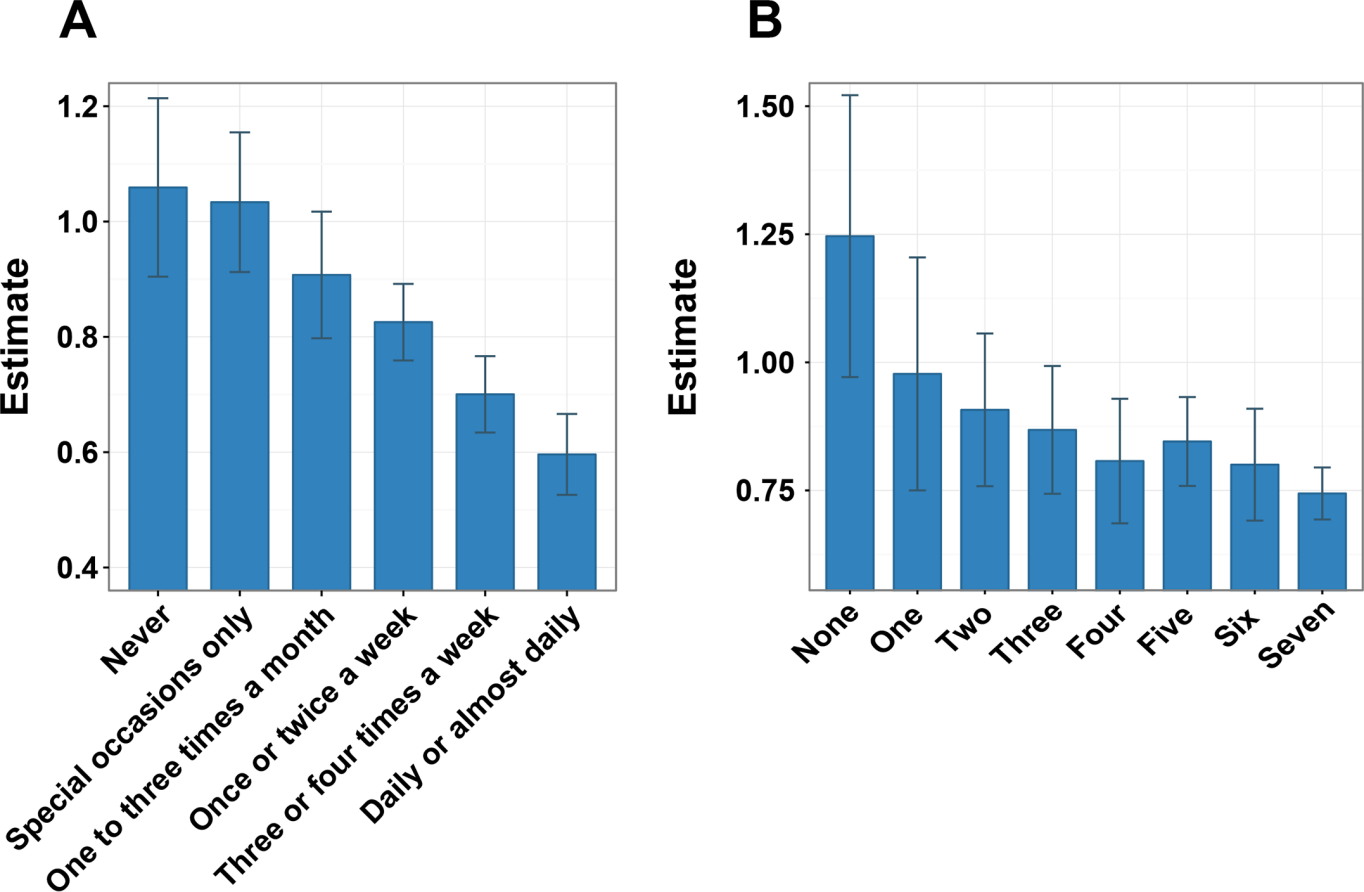
**Interaction between *GS***_***BMI***_ **genotype with frequency of alcohol consumption (A) and frequency of more than 10 minutes of walking per week (B).** (A) Effect on BMI per *GS*_*BMI*_ by frequency of alcohol intake. The self-report questionnaire asked: “About how often do you drink alcohol?” The effect per *GS*_*BMI*_ is higher in low-frequency alcohol consumers compared to high-frequency consumers: (“Never”: N = 7,944; “Special occasions only”: N = 12,767; “once or twice a week”: N = 12,966; “One to three times a month”: N = 30,412; “Three or four times a week”: 27,250; “Daily or almost daily”: N = 24,424.) (B) Effect on BMI per *GS*_*BMI*_ by frequency of 10+ minutes of walking. The self-report questionnaire asked: “In a typical week, on how many days did you walk for at least 10 minutes at a time? (Include walking that you do at work, travelling to and from work, and for sport or leisure).” (“None”: N = 2,528; “One”: N = 7,046; “Two”: 7,046; “Three”: 9,215; “Four”: N = 9,393; “Five”: 18,441; “Six”: N = 11,334; “Seven”: N = 53,125). Error bars represent 95% CI.

**Fig 3:**
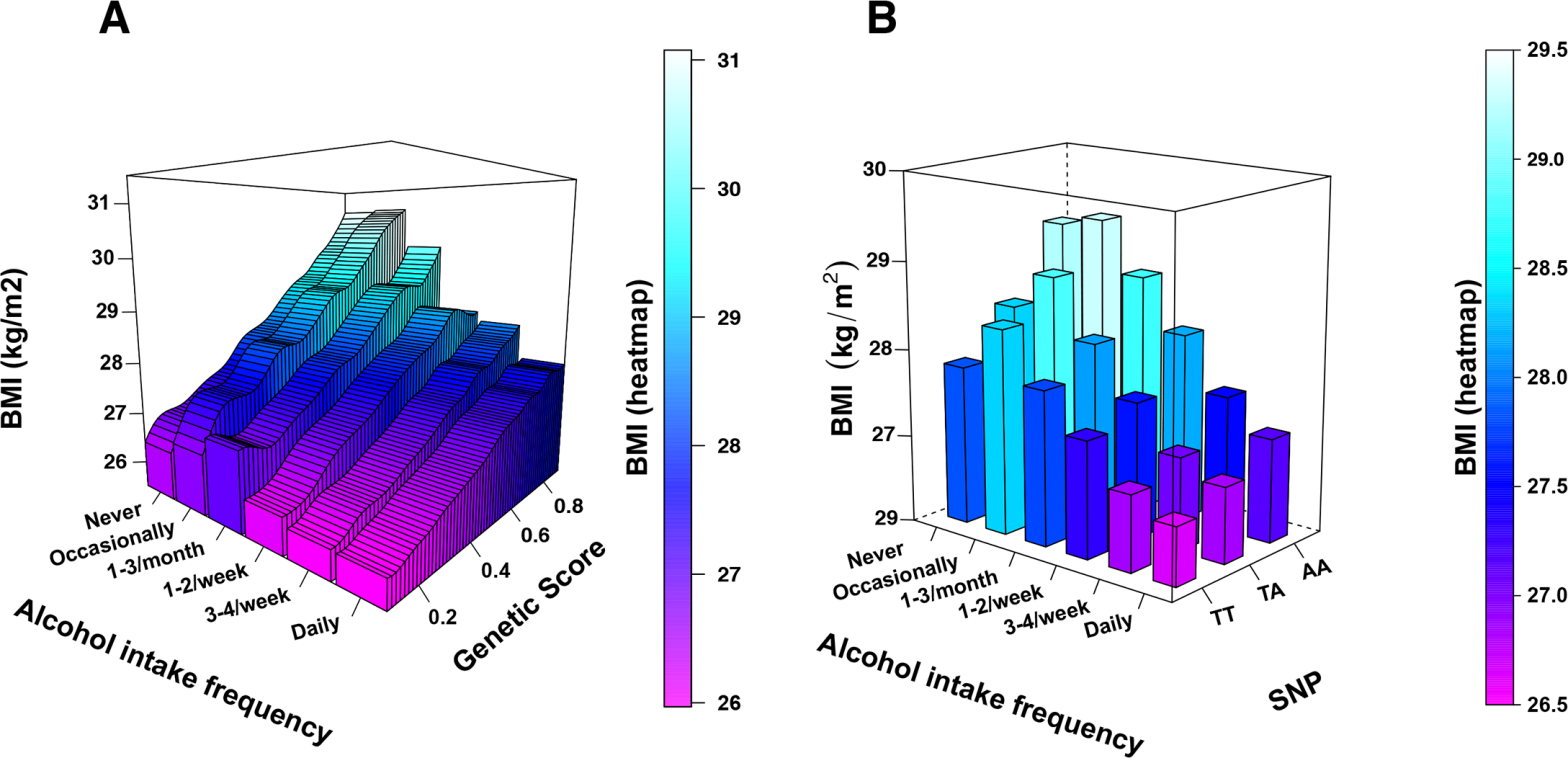
**Interaction between (A) *GS***_***BMI***_ **and (B) rs1558902, with alcohol intake frequency.** (A) BMI was plotted against *GS*_*BMI*_. and stratified by alcohol consumption frequency. The effect of *GS*_*BMI*_, i.e., the increase in BMI with *GS*_*BMI*_, was lower in UK Biobank participants who consume alcohol less frequently, compared to participants who consume alcohol more frequently. (B) Mean BMI per genotype of the FTO-linked SNP, rs1558902, is plotted by frequency of alcohol intake. The effect of rs1558902, i.e. the increase in BMI with copies of the A-allele, was lower in high-frequency alcohol consumers, and higher in low-frequency consumers.

Strong evidence for interaction with *GS*_*BMI*_ was seen for alcohol intake frequency (*p* = 1.45*10^−29^) with larger effect of *GS*_*BMI*_ in infrequent drinkers (Fig 2A, Tables 2 and 3, S5 Table). The effect of *GS*_*BMI*_ decreased, in a dose-dependent manner, as alcohol consumption frequency increased and the effect of *GS*_*BMI*_ was less than half the effect in everyday drinkers compared to infrequent drinkers (Fig 2A). The interaction between *GS*_*BMI*_ and alcohol intake frequency means that the increase in BMI per *GS*_*BMI*_ unit is higher in infrequent drinkers compared to more frequent drinkers (Fig 3A). In addition to the interaction between *GS*_*BMI*_ and alcohol intake frequency, we also observe a highly significant inverse association between alcohol intake frequency and BMI (S3 Fig, *p* < 2.2*10^−308^).

In contrast to alcohol frequency, we were unable to observe significant interactions with the number of alcoholic beverages per week or total weekly alcohol intake (gram/week). An interaction was observed with total weekly intake of red wine in the discovery cohort, where higher genetic effects were associated with low weekly consumption of red wine (Table 2, S5 Table). However, this effect was not observed in the replication cohort (Table 3).

Interactions were also identified for several factors related to physical activity, such as: usual walking pace, stair climbing, and TV watching, as well as frequencies of light- (Fig 3B), moderate-, as well as vigorous exercise (Tables 2 and 3, S6 Table). Particularly strong evidence was found for an interaction with walking pace, (*p* = 3.38*10^−26^, Table 2). While the frequencies of physical activity were interacting with *GS*_*BMI*_, no significant interactions were identified for durations of physical activity (S6 Table).

Several markers of socioeconomic status were observed to interact with *GS*_*BMI*_ in the discovery cohort including: Townsend deprivation index (TDI), as well as number of vehicles in household and total household income (Tables 2 and 3, S7 Table). TDI is a composite score for socioeconomic status that is generated for each national census output area and incorporates area inhabitants’ unemployment rates, car- and house-ownership as well as the number of people in a household. Higher TDI corresponds to a larger degree of social deprivation and was associated with an increased effect of *GS*_*BMI*_ (Tables 2 and 3, S7 Table).

We also observed significant interactions with factors related to depression including: fed-up feelings and frequency of depressed mood (Tables 2 and 3, S8 Table). Higher effects of *GS*_*BMI*_ were observed in participants who reported often feeling fed-up, and in participants who reported higher frequency of feeling down, depressed or hopeless (data field 2050). Interactions were also observed for factors related to sleeping patterns with higher genetic effects in the group that often take a nap during the day and in the group that often felt tired or reported having low energy. Significant interactions were also identified for smoking status (Tables 2 and 3, S5 Table) and number of treatments/medications taken (Tables 2 and 3, S9 Table), where smokers had a higher effect of *GS*_*BMI*_ compared to non-smokers and where the genetic effects increased with number of treatments/medications taken (Table 3).

We followed up the findings from the main analyses by performing linear regression modeling on individual SNPs that were included in *GS*_*BMI*_ to study their interactions with lifestyle factors (supplementary data). These analyses resulted in large number of statistical tests (94 SNPs times 131 lifestyle factors = 12,314 tests), which reduces the statistical power. Adjusting for all tests performed using Bonferroni or FDR resulted in only one significant interaction between rs1558902 at the FTO locus and usual walking pace (Table 4). However, using the same *p*-value cut-off as for *GS*_*BMI*_, (*p* = 3.82 *10^−4^) interactions were found for eleven lifestyle factors in the discovery cohort, of which three interactions with the *FTO* SNP rs1558902 could be replicated (Table 4). Rs1558902 was observed to interact with total household income, usual walking pace and alcohol intake frequency (Table 4). Previous GWAS has estimated the effect of the *FTO* variant, rs1558902, to 0.39 kg/m^2^ per A-allele [5]. Stratification by frequency of alcohol intake revealed the effect of rs1558902 to be 0.64 kg/m^2^ per A-allele in non-drinkers, and to be attenuated to 0.25 kg/m^2^ per A-allele in participants that drink daily or almost daily. The interaction between rs1558902 and alcohol intake frequency means that, the effect of rs1558902, i.e. the increase in BMI per allele, is lower in high-frequency alcohol consumers, and higher in low-frequency consumers (Fig 3B).

**Table 4:** Interactions between individual SNPs and lifestyle factors.

**Lifestyle factor**	**SNP**	**Closest protein coding gene(s)**	***Discovery***		***Replication***	
			*β1*	*p1*	*β2*	*p2*
738: Average total household income	rs1558902	*FTO*	-1.45*10^−2^	3.43*10^−4^	-1.08*10^−2^	1.09*10^−4^
924: Walking pace	rs1558902	*FTO*	-3.41*10^−2^	1.96*10^−7^	-2.82*10^−2^	7.04*10^−10^
1070: Time spent watching television	rs16851483	*RASA2*	2.22*10^−2^	9.01*10^−5^	-2.03*10^−3^	5.81*10^−1^
1190: Nap during day	rs1016287	*FLJ30838*	-2.85*10^−2^	1.67*10^−4^	-4.12*10^−3^	4.34*10^−1^
1438: Bread intake	rs12885454	*FOXG1/PRKD1*	2.08*10^−3^	3.81*10^−4^	4.16*10^−4^	2.60*10^−1^
1558: Alcohol intake Frequency	rs3810291	*ZC3H4*	1.14*10^−2^	1.27*10^−4^	-4.85*10^−3^	1.89*10^−2^
1558: Alcohol intake Frequency	rs1558902	*FTO*	1.15*10^−2^	4.89*10^−5^	9.99*10^−3^	3.32*10^−7^
2080: Frequency of tiredness/lethargy in last 2 weeks	rs1516725	*ETV5*	2.81*10^−2^	1.06*10^−4^	-7.18*10^−3^	1.57*10^−1^
2090: Seen doctor (GP) for nerves, anxiety, tension or depression	rs2650492	*SBK1*	4.07*10^−2^	2.69*10^−5^	-6.70*10^−3^	3.14*10^−1^
4581: Financial situation satisfaction	rs1558902	*FTO*	2.86*10^−2^	9.66*10^−5^	5.44*10^−3^	3.14*10^−1^
20161: Pack years of smoking	rs11847697	*PRKD1*	3.67*10^−3^	1.96*10^−4^	6.12*10^−4^	4.74*10^−1^
Estimated weekly consumption of alcohol (g)	rs11057405	*CLIP1*	-1.28*10^−4^	2.58*10^−4^	4.01*10^−5^	1.45*10^−1^

*p1-2*: p-value for tests of the estimated effect size deviating from zero. *β1–2*: Estimated effect size of the interaction terms. p-values < 3.82*10^-4^ were considered significant.

### Sensitivity analyses

To test whether interactions were driven primarily by the *FTO*-SNP rs1558902, we constructed a genetic score for BMI with rs1558902 excluded (*GS*_*BMI*_*’*). In the discovery analysis, all Bonferroni significant interaction terms from the previous analyses remained significant when we used *GS*_*BMI*_*’*, except for smoking status and number of treatments/medications taken (Table 2, S4–S9 Tables). Effect estimates for all significant interactions were in the same direction and within 15% of the interaction effects with the previous *GS*_*BMI*_. We also utilized *GS*_*BMI*_*’* in the replication cohort, and replication was successful for all 15 interactions observed with *GS*_*BMI*_, except for frequency of depressed mood and smoking status (Table 3).

We also performed sensitivity analyses by including TDI and its interaction terms as covariates in linear regression models. These results were highly correlated with previous results (Pearson’s *r* = 0.98 for effect estimates of interaction terms). Including TDI as a covariate led to a general slight decrease in effect sizes of interaction terms. The largest decreases were seen for factors related to socioeconomic status (S11 Table) and smoking status, which is consistent with the highly significant correlation between these variables and TDI (S12 Table).

In the present study, we utilised SNP effect estimates from a previous GWAS by the GIANT consortium [4] to calculate the genetic score for BMI. These estimates may be somewhat overestimated due to the “winner’s curse” [21]. Using overestimated effect sizes results in a *GS*_*BMI*_ that is associated with a slightly lower BMI-increase in UK Biobank, compared to when using the correct effect size estimates. This can clearly be seen in our data since a one-unit increase in the *GS*_*BMI*_ results in a 0.82 unit increase in the rank transformed BMI in UK Biobank. We therefore also tested interaction effects using a genetic score composed of SNP effect estimates calculated in the UK Biobank cohort (*GS*_*BMI*_*_UKBB*), so that a single-standard-unit increase in the *GS*_*BMI*_*_UKBB* results in an exactly 1.00 unit increase in the rank-transformed BMI, which is 22% higher compared to 0.82 for *GS*_*BMI*_. The interaction results were also strongly correlated between using *GS*_*BMI*_*_UKBB or GS*_*BMI*_ (Pearson’s *r* = 0.99 for effect estimates of interaction terms; S11 Table). However, the effect estimates for FDR-significant interaction terms were, not surprisingly, on average 16% larger when we utilised *GS*_*BMI*_*_UKBB* (S11 Table).

### Stepwise linear regression

Several of the exposures that were found to interact with *GS*_*BMI*_ in our primary analysis showed highly significant evidence for correlation with one another (S12 Table). In order to identify the most informative interacting variables and interaction terms for a predictive model for BMI, we performed SLR on a combined set of the discovery and replication cohort. SLR was performed in both directions using the 15 lifestyle factors whose interactions with *GS*_*BMI*_ were replicated (Table 3). This resulted in inclusion of 290,441 participants with non-missing data when combining the discovery and replication cohorts. The final model generated by SRL included gene-environment interaction terms for ten lifestyle factors (S10 Table, supplementary Data), of which eight were nominally significant: alcohol intake frequency (*p* = 2.82*10^−15^), usual walking pace (*p* = 1.55*10^−14^), frequency of 10+ minute walks (*p* = 6.26*10^−4^), smoking status (p = 1.45*10^−3^), frequency of vigorous exercise (*p* = 2.28*10^−3^), number of vehicles in household (p = 1.03*10^−2^), TDI (p = 3.02*10^−2^) and frequency of tiredness/lethargy (p = 3.87*10^−2^) and. The adjusted *R*^*2*^ value for the final model was 0.1957.

## Discussion

In this study, we performed a gene-environment interaction study using genetic variants and self-reported lifestyle data. We identified several lifestyle factors that influence the effect of genetic variants on BMI. Interactions were observed for factors related to alcohol intake, physical activity, socioeconomic status, mental health and sleeping patterns. Interactions were seen for factors related to physical activity, where a more active lifestyle attenuated the genetic effects, which is consistent with previous reports [6–9]. Interactions were observed for light, moderate intensity, and vigorous physical activity. However, we observed that the interaction between physical activity and the genetic score was strong for frequencies of physical activity, in contrast to durations in minutes/day.

Strong evidence was also observed for an interaction with frequency of alcohol intake. The genetic effect was attenuated with higher frequency of alcohol intake in an almost dose-dependent manner with twice as large effects in non-drinkers compared to daily drinkers. Alcohol consumption is common in western societies, where also most previous GWAS have been performed. Our results indicate that the interaction associated with alcohol intake frequency may have partially attenuated the full effect of BMI-associated genetic variants observed in previous association studies.

Alcohol intake frequency was also associated with lower average BMI. This is consistent with clinical reports of lower BMI and fat mass in severely alcoholic patients [22–24]. *In vitro* and *in vivo* experiments have also shown ethanol exposure to increase lipolysis and reduce white adipose tissue mass [25,26]. This can also be compared to data from the National Institute on Alcohol Abuse and Alcoholism (NIAAA)[27], which suggests that moderate daily consumption of alcoholic beverages, 1–2 drinks per day, reduces the risk of myocardial infarction as well as all-cause mortality [27]. In addition, a cohort study on 38,077 male health professionals reported that alcohol consumption frequency, rather than total amounts, was the primary determinant of the inverse association between alcohol consumption and risk of myocardial infarction [28]. Unfortunately, we do not have data on UK Biobank participants’ daily consumption amounts and we are unable to determine how this factors into the association between alcohol consumption frequency, BMI and the interaction between alcohol intake frequency and *GS*_*BMI*_.

In a previous study on gene-environment interactions in the UK Biobank, Tyrrell *et al*. used a genetic score composed of 69 BMI-associated variants to study interactions with measurements of the obesogenic environment, with focus on physical activity, diet and socioeconomic status [29]. Interactions were identified with measurements of physical activity and socioeconomic status (TDI) [29] which were consistent with the current study. TDI serves as a proxy for environmental and lifestyle factors that are correlated with income and social position. The study by Tyrell *et al*. study contrasts the current in the selection of twelve obesogenic factors. The current study instead utilised a hypothesis-free approach to test interactions between *GS*_*BMI*_ and 131 factors, which allows us to contrast aspects of the same behaviour, e.g., between amounts of physical activity and frequency and also gives us the potential to identify new gene-environment interactions. A drawback to this approach is the increased statistical power required in order to correct for the family-wise error rate.

In our primary analyses, we have investigated interactions between lifestyle factors and a genetic score composed by 94 independent SNPs, located in different loci. SNPs were combined into genetic scores that explain a greater amount of the variation in BMI compared to the individual SNPs, in order to gain statistical power (S1 Supporting Information). These SNPs have previously been shown to influence BMI [4]. However, combining them into a genetic score before testing for interactions with lifestyle factors assumes that the interaction effect of the BMI-increasing alleles are all in the same direction: e.g., that alcohol intake frequency decreases the genetic effects of all SNPs rather than some genetic effects being larger among frequent drinkers and others larger among non-drinkers. For alcohol intake frequency, we have the statistical power to detect an interaction if interaction effects in the same direction are present for at least 37 of the SNPs (S1 Supporting Information). However, if some SNPs are interacting in the opposite direction, our power will decrease dramatically (S1 Supporting Information). It is therefore possible that there are gene-environment interactions that are masked by SNPs having interaction effects with the same environmental factor, but in opposite directions. For this reason, we also performed follow-up analyses of individual SNPs. These analyses revealed that the *FTO*-linked SNP rs1558902, in addition to interacting with alcohol intake frequency, also interacts with average total household income and physical activity.

For BMI, as well as for other complex traits, knowledge on the biological implications of associated genetic variation is limited, which impedes deduction of causal mechanisms underlying gene-environment interactions. The *FTO* variant, rs1558902, is associated with the expression of two upstream genes (*IRX3* and *IRX5*) which affect adipocyte “browning”, i.e. the occurrence of thermogenic ‘beige’ adipocytes in white adipose tissue depots. This may partly explain the observed interaction between rs1558902 and frequency of alcohol intake, as *in vitro* experiments have shown that ethanol exposure interferes with mobilization of glucose transporters to the adipocyte cellular membrane in response to insulin [30]. Beige adipocytes, on the other hand, are able to take up glucose from the circulation in an insulin-independent manner [31]. The altered lipolysis in white adipose tissue due to ethanol exposure, in combination with an altered rate of thermogenesis due to differential propensity for adipocyte browning between individuals with different rs1558902 genotypes may explain the interaction between this SNP and frequency of alcohol intake.

Enrichment analyses from previous GWAS have also implicated central nervous system processes to play an important role in BMI [4,5]. The central nervous system contains regions that regulate several functions related to BMI, such as appetite, homeostasis, reward, and motivation. Ethanol confers several well-known behavioural effects on humans, but also acts in a bi-phasic manner as a central nervous system stimulant at low doses, and a general depressant at higher doses [32]. BMI-associated genetic variants that affect BMI-related central nervous system function may also factor into the observed interaction between alcohol consumption frequency and *GS*_*BMI*_.

A possible limitation to our study is responder bias in the self-report questionnaire data. This may be more likely for factors pertaining to self-image such as alcohol, tobacco use and physical activity. The lack of an interviewing person, and assuring participants of the confidentiality and anonymity of their data aim to reduce the likelihood of responder bias [33]. We tested the validity of factors related to alcohol consumption and physical activity by comparing these to data collected through a 24-hour recall questionnaire. We observe that both frequency and amounts of alcohol consumption, as well as measurements of frequency and duration of physical exercise, agreed well with 24-hour recall data, which supports the validity of these measurements (S2 Supporting Information).

In this study, we primarily investigated associations, and the underlying causal mechanisms behind gene-environment interactions are difficult to deduce from cross-sectional data alone. We constructed separate models for each environmental exposure or lifestyle factor. As such, it is important to be aware that confounding effects of factors that are not included in the models, or that are unknown, may be present in the results from these tests. In order to fully correct for confounding factors and correctly characterise causal factors, controlled experiments such as clinical trials in controlled settings serve as the gold standard. We have attempted to correct for confounding by including TDI and the interaction term TDI*GS_*BMI*_ as covariates in all analyses, which resulted in very little effect on the main results. To identify factors with the highest predictive value, we also performed SLR, which showed evidence for interactions between *GS*_*BMI*_ and alcohol consumption frequency, physical activity, smoking, and socioeconomic status all contributed independently to a predictive model for BMI.

In conclusion, the standardised collection of genetic and lifestyle data in UK Biobank has enabled us to identify several factors that modify the effect of BMI-associated genetic variants. The most significant interactions were observed between *GS*_*BMI*_ and frequency of alcohol intake, frequency of physical activity and socioeconomic status. Previous studies have reported interactions between genetic variants at the *FTO* locus and environmental factors [6–9]. However, most interactions were still observed even when the *FTO* locus was excluded from the genetic score, which indicates that the individual interactions are not solely dependent on *FTO* variants. We can therefore conclude that the presence of genetic interactions is more general and will be identified to a higher degree for individual SNPs once the sample size increases even more and reaches sufficient power.

## Supporting information

S1 Fig Comparison of effect estimates for the 94 SNPs included in the construction of the *GS*_*BMI*_ between UK Biobank and previously published BMI GWAS from the GIANT consortium [4]. (PDF)

S2 Fig **(A) Histogram of BMI for the 116,138 participants from the UK Biobank Resource.** (B) BMI was normalized by rank transformation.
(PDF)

S3 Fig Average BMI ± 95% CI stratified by self reported frequency of alcohol intake (variable 1558). (PDF)

S1 Table Environmental variables that were included in the interaction analyses, along with their corresponding coding and a brief description of their data distribution. Quantitative variables are described by average ± standard deviation. Ordinal variables are described by the number and percentage of individuals in each category.
(DOCX)

S2 Table Results from tests for deviation from Hardy Weinberg equilibrium (HWE) and association with BMI in the UK Biobank cohort. Three SNPs deviated from HWE and were excluded from further analysis. HWE: p-values from tests for deviation from hardy Weinberg equilibrium. *β*: estimated *β*-values from linear regression models. *β*_se: standard error for estimated beta-values. *p*: p-values for association tests. *p-adj: p-values corrected for multiple testing using the Bonferroni method. Power indicates the power to replicate, with Bonferroni adjusted p-value < 0.05, the in UK biobank considering the effect size and allele frequency of each SNP in GIANT.
(DOCX)

S3 Table Comparison of SNP-effects on BMI between male and female participants of UK Biobank. *β* is the effect size estimate of the SNP, *SE β* is the standard error of the effect size estimate, and *p* is the corresponding p-value from association tests between SNPs and BMI. *p*^*#*^—p-values from student’s two-sample t-tests to compare means between males and females. *p*-adj*—p values adjusted for multiple testing using the Bonferroni method.
(DOCX)

S4 Table Effect by, and interaction with genetic risk score for dietary habits, assessed by self-report touchscreen questionnaire. N: number of individuals included in the respective analyses. *E*: the results, with corresponding estimates (*β)* and p-values *(p)* for the linear models testing for the effect on each lifestyle variable on BMI without including the interaction term. *GS*_*BMI*_ *× E*: Results for the interaction term from linear models for association with the genetic score for BMI composed of the effects of 94 SNPs associated with BMI. *β2*: Estimated effect sizes of the interaction. *p2*: p-value for tests of the estimated effect size deviating from zero. *GS*_*BMI*_*' × E* is the genetic score for BMI excluding the *FTO* SNP rs1558902 with corresponding estimates (*β3)* and p-values *(p3)* for the interaction terms.
(DOCX)

S5 Table Effect by, and interactions between genetic risk score for BMI and smoking, and alcohol consumption habits, assessed by self-report touchscreen questionnaire. N: number of individuals included in the respective analyses. *E*: the results, with corresponding estimates (*β)* and p-values *(p)* for the linear models testing for the effect on each lifestyle variable on BMI without including the interaction term. *GS*_*BMI*_ *× E*: Results for the interaction term from linear models for association with the genetic score for BMI composed of the effects of 94 SNPs associated with BMI. *β2*: Estimated effect sizes of the interaction. *p2*: p-value for tests of the estimated effect size deviating from zero. *GS*_*BMI*_*' × E* is the genetic score for BMI excluding the *FTO* SNP rs1558902 with corresponding estimates (*β3)* and p-values *(p3)* for the interaction terms.
(DOCX)

S6 Table Effect by, and interactions between genetic risk score for BMI and physical activity, assessed by self-report touchscreen questionnaire. N: number of individuals included in the respective analyses. *E*: the results, with corresponding estimates (*β)* and p-values *(p)* for the linear models testing for the effect on each lifestyle variable on BMI without including the interaction term. *GS*_*BMI*_ *× E*: Results for the interaction term from linear models for association with the genetic score for BMI composed of the effects of 94 SNPs associated with BMI. *β2*: Estimated effect sizes of the interaction. *p2*: p-value for tests of the estimated effect size deviating from zero. *GS*_*BMI*_*' × E* is the genetic score for BMI excluding the *FTO* SNP rs1558902 with corresponding estimates (*β3)* and p-values *(p3)* for the interaction terms.
(DOCX)

S7 Table Effect by, and interactions between genetic risk score for BMI and socioeconomic factors. **N: number of individuals included in the respective analyses.** *E*: the results, with corresponding estimates (*β)* and p-values *(p)* for the linear models testing for the effect on each lifestyle variable on BMI without including the interaction term. *GS*_*BMI*_ *× E*: Results for the interaction term from linear models for association with the genetic score for BMI composed of the effects of 94 SNPs associated with BMI. *β2*: Estimated effect sizes of the interaction. *p2*: p-value for tests of the estimated effect size deviating from zero. *GS*_*BMI*_*' × E* is the genetic score for BMI excluding the *FTO* SNP rs1558902 with corresponding estimates (*β3)* and p-values *(p3)* for the interaction terms.
(DOCX)

S8 Table Effect by, and interactions between genetic risk score for BMI and mental health factors. **N: number of individuals included in the respective analyses.** *E*: the results, with corresponding estimates (*β)* and p-values *(p)* for the linear models testing for the effect on each lifestyle variable on BMI without including the interaction term. *GS*_*BMI*_ *× E*: Results for the interaction term from linear models for association with the genetic score for BMI composed of the effects of 94 SNPs associated with BMI. *β2*: Estimated effect sizes of the interaction. *p2*: p-value for tests of the estimated effect size deviating from zero. *GS*_*BMI*_*' × E* is the genetic score for BMI excluding the *FTO* SNP rs1558902 with corresponding estimates (*β3)* and p-values *(p3)* for the interaction terms.
(DOCX)

S9 Table Effect by, and interactions between genetic risk score for BMI and general factors related to sleep, health and female-specific factors. N: number of individuals included in the respective analyses. *E*: the results, with corresponding estimates (*β)* and p-values *(p)* for the linear models testing for the effect on each lifestyle variable on BMI without including the interaction term. *GS*_*BMI*_ *× E*: Results for the interaction term from linear models for association with the genetic score for BMI composed of the effects of 94 SNPs associated with BMI. *β2*: Estimated effect sizes of the interaction. *p2*: p-value for tests of the estimated effect size deviating from zero. *GS*_*BMI*_*' × E* is the genetic score for BMI excluding the *FTO* SNP rs1558902 with corresponding estimates (*β3)* and p-values *(p3)* for the interaction terms.
(DOCX)

S10 Table Results from the final model generated by the stepwise regression model. Results for interactions between *GS*_*BMI*_ and environmental factors are shown. Results for the full model are available in the supplementary Data. Stepwise linear regression was performed as was described in the methods section. Age, age^2^ and sex were included as covariates. Interaction terms for all secondary interactions were included to control for potential confounding. The model also included a batch variable for the two genotyping platforms used in the UK Biobank, as well as 15 principal components. *p*-values < 0.05 were considered significant.
(DOCX)

S11 Table Interactions between environmental factors and *GS*_*BMI*_ when Townsend deprivation index (TDI) is included as a covariate in linear regression models, and when the genetic score is composed of effect estimates generated in UK Biobank (*GS*_*BMI*_*_UKBB*). N: number of individuals included in the respective analyses. *β1–3*: Estimated effect sizes of the interaction terms. *p1-3*: p-value for tests of the estimated effect size deviating from zero.
(DOCX)

S12 Table Results from Kendall rank correlation tests between the 19 environmental variables that were observed to interact with GSBMI. Values in the bottom diagonal represent Kendall tau coefficients (*τ*). Values in the upper diagonal represent p-values.
(DOCX)

S1 Data SNP-environment interactions for each individual SNP and environmental factors and results from stepwise linear regression analysis (SLR). Tests for SNP-environment interactions were performed for each individual SNP and environmental factor according to Eq (2) (see methods). Each tab contains data from linear regression models for all SNPs. The coding for each environmental factor is available in S1 Table. Beta-estimate—The estimated effect size of the interaction term. Standard error—The standard error for the estimate. t-value—t-test statistic. p—p-value for deviation from zero of the estimated interaction term.
(XLSX)

S1 Supporting Information Power-calculations. (DOCX)

S2 Supporting Information Validation of alcohol and physical activity data. (DOCX)
