# Supplementary figures and images for: Gene-environment interaction study for BMI reveals interactions between genetic factors and physical activity, alcohol consumption and socioeconomic status
Source: PLoS Genet. 2017 Sep 5;13(9):e1006977. doi: 10.1371/journal.pgen.1006977 (PMC5600404; doi:10.1371/journal.pgen.1006977)

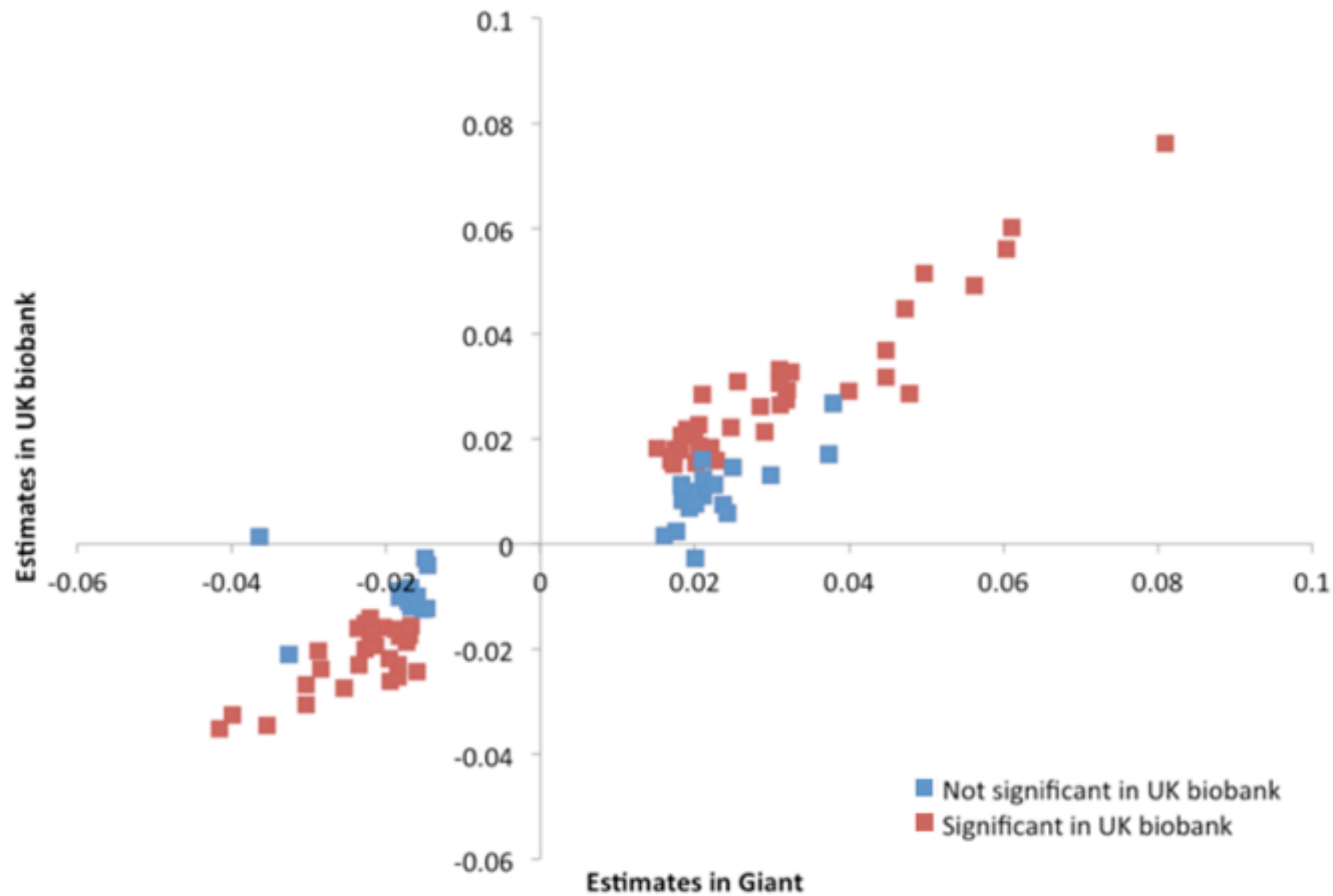

Supplement: S1 Fig — (PDF) [file pgen.1006977.s001.pdf]

**A**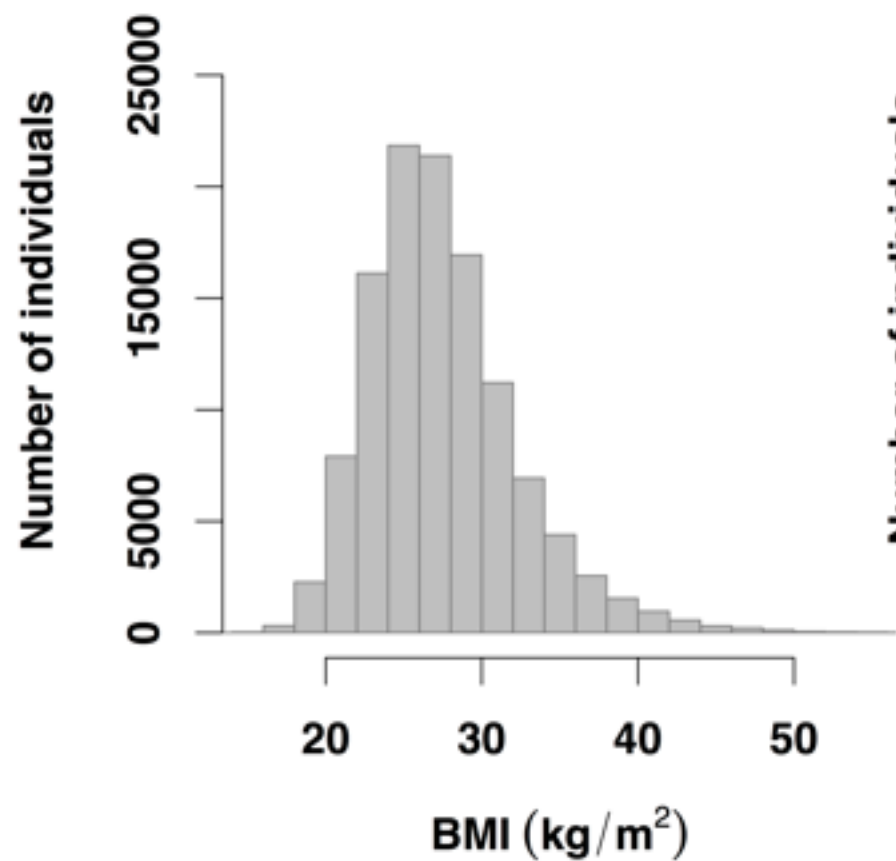**B**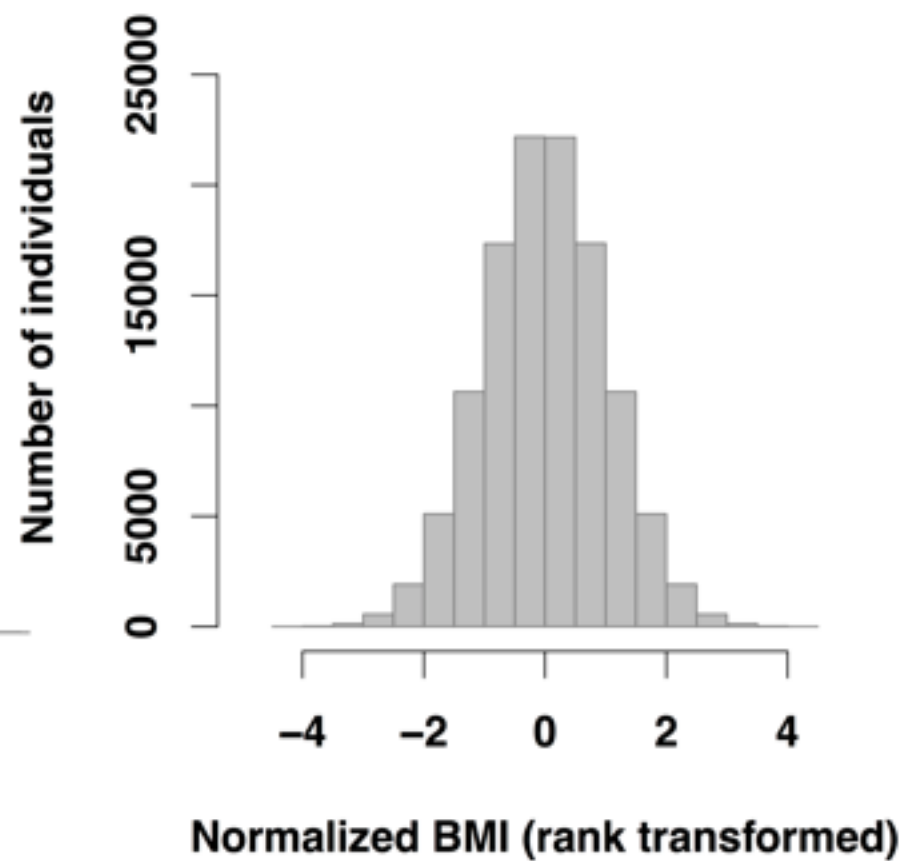

Supplement: S2 Fig — (A) Histogram of BMI for the 116,138 participants from the UK Biobank Resource. (B) BMI was normalized by rank transformation. (PDF) [file pgen.1006977.s002.pdf]

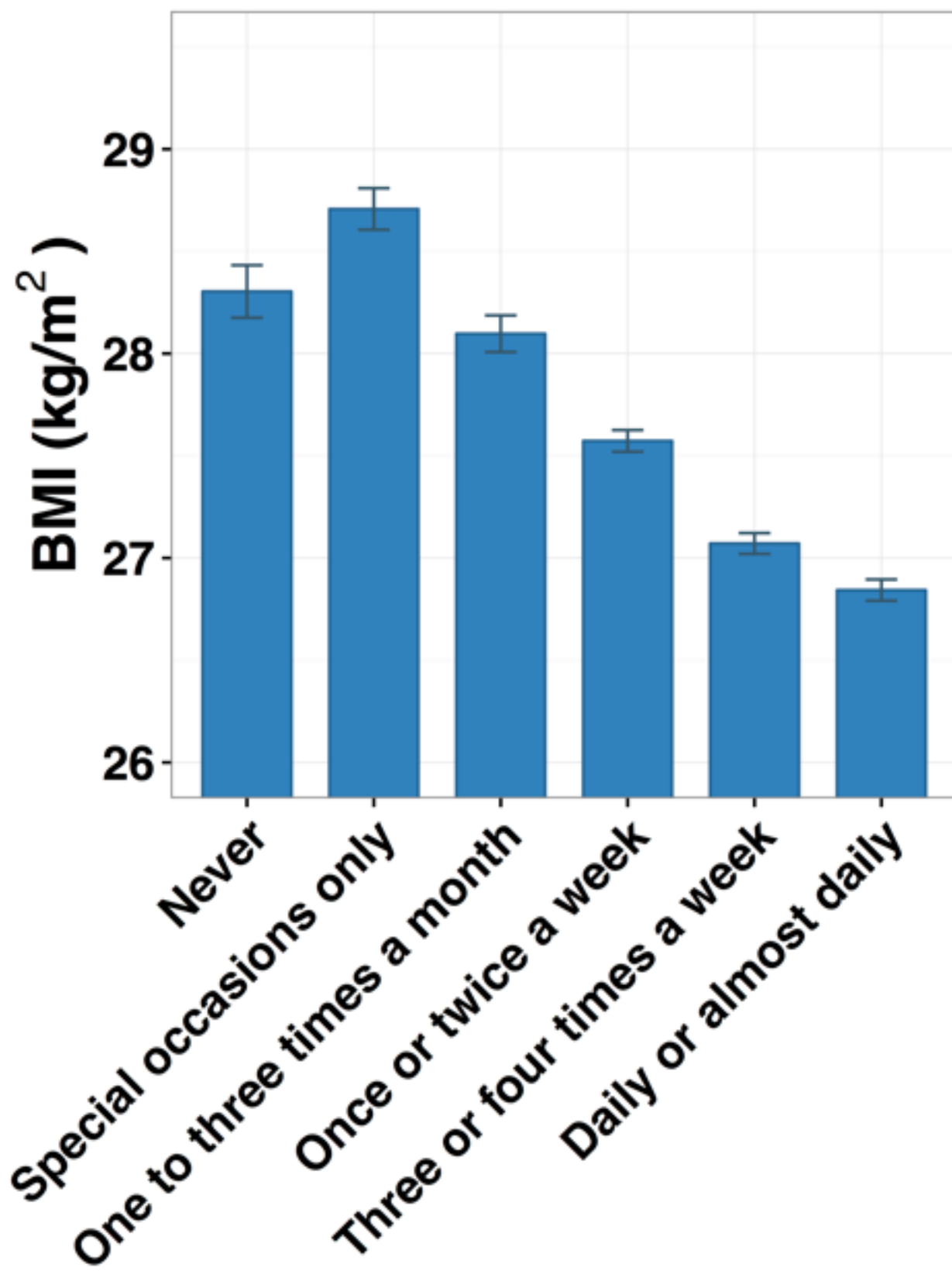

Supplement: S3 Fig — (PDF) [file pgen.1006977.s003.pdf]
