# Supplementary material for: Gene-environment interaction study for BMI reveals interactions between genetic factors and physical activity, alcohol consumption and socioeconomic status
Source: PLoS Genet. 2017 Sep 5;13(9):e1006977. doi: 10.1371/journal.pgen.1006977 (PMC5600404; doi:10.1371/journal.pgen.1006977)
Supplement: S1 Table — Quantitative variables are described by average ± standard deviation. Ordinal variables are described by the number and percentage of individuals in each category. (DOCX) [file pgen.1006977.s004.docx]

**S1 Table. Environmental variables that were included in the interaction analyses, along with their corresponding coding and a brief description of their data distribution.**

| **ID** | **Name** | **Coding / Unit** | **Distribution of data** |
| --- | --- | --- | --- |
| 1289 | Cooked vegetable intake | Tablespoons/day | 2.62 ± 1.34 |
| 1299 | Salad / raw vegetable intake | Tablespoons/day | 2.11 ± 1.69 |
| 1309 | Fresh fruit intake | Pieces/day | 2.15 ± 1.34 |
| 1319 | Dried fruit intake | Pieces/day | 0.70 ± 1.20 |
| 1329 | Oily fish intake | 0 = Never, 1 = Less than once a week, 2 = Once a week, 3 = 2-4 times a week, 4 = 5 = Once or more daily | 13032, 38727, 43565, 19307, 761, 215  (11.3%, 33.5%, 37.7%, 16.7%, 0.7%, 0.2%) |
| 1339 | Non-oily fish intake | 0 = Never, 1 = Less than once a week, 2 = Once a week, 3 = 2-4 times a week, 4 = 5 = Once or more daily | 4913, 33528, 58641, 17994, 484, 146  (4.2%, 29%, 50.7%, 15.6%, 0.4%, 0.1%) |
| 1349 | Processed meat intake | 0 = Never, 1 = Less than once a week, 2 = Once a week, 3 = 2-4 times a week, 4 = 5 = Once or more daily | 9199, 34425, 34665, 32914, 3823, 940  (7.9%, 29.7%, 29.9%, 28.4%, 3.3%, 0.8%) |
| 1359 | Poultry intake | 0 = Never, 1 = Less than once a week, 2 = Once a week, 3 = 2-4 times a week, 4 = 5 = Once or more daily | 5347, 12349, 42420, 53479, 2133, 227  (4.6%, 10.6%, 36.6%, 46.1%, 1.8%, 0.2%) |
| 1369 | Beef intake | 0 = Never, 1 = Less than once a week, 2 = Once a week, 3 = 2-4 times a week, 4 = 5 = Once or more daily | 10700, 53405, 37911, 13492, 169, 59  (9.2%, 46.1%, 32.8%, 11.7%, 0.1%, 0.1%) |
| 1379 | Lamb/mutton intake | 0 = Never, 1 = Less than once a week, 2 = Once a week, 3 = 2-4 times a week, 4 = 5 = Once or more daily | 19997, 66642, 25988, 2847, 16, 25  (17.3%, 57.7%, 22.5%, 2.5%, 0%, 0%) |
| 1389 | Pork intake | 0 = Never, 1 = Less than once a week, 2 = Once a week, 3 = 2-4 times a week, 4 = 5 = Once or more daily | 17202, 67549, 26870, 3795, 80, 43  (14.9%, 58.5%, 23.3%, 3.3%, 0.1%, 0%) |
| 1408 | Cheese intake | 0 = Never, 1 = Less than once a week, 2 = Once a week, 3 = 2-4 times a week, 4 = 5 = Once or more daily | 3081, 18866, 24360, 52595, 10731, 3898  (2.7%, 16.6%, 21.5%, 46.3%, 9.5%, 3.4%) |
| 1438 | Bread intake | Slices/week | 12.29 ± 7.77 |
| 1458 | Cereal intake | Bowl/week | 4.67 ± 2.62 |
| 1478 | Salt added to food | 1 = Never/rarely, 2 = Sometimes, 3 = Usually, 4 = Always | 64668, 32207, 13700, 5551  (55.7%, 27.7%, 11.8%, 4.8%) |
| 1488 | Tea intake | Cups/day | 3.46 ± 2.55 |
| 1498 | Coffee intake | Cups/day | 2.20 ± 2.02 |
| 1518 | Hot drink temperature | 1 = Very hot, 2 = Hot, 3 = Warm | 19555, 77683, 17690  (17%, 67.6%, 15.4%) |
| 1528 | Water intake | Glasses/day | 2.67 ± 2.02 |
| 1548 | Variation in diet | 1 = Never/rarely, 2 = Sometimes, 3 = Often | 40322, 66434, 9097  (34.8%, 57.3%, 7.9%) |
| 1558 | Alcohol intake frequency. | 1 = Daily or almost daily, 2 = Three or four times a week, 3 = Once or twice a week, 4 = One to three times a month, 5 = Special occasions only, 6 = Never. | 24724, 27250, 30412, 12966, 12767, 7944  (21.3%, 23.5%, 26.2%, 11.2%, 11%, 6.8%) |
| 1568 | Average weekly red wine intake | Number of glasses (125ml) | 3.59 ± 4.69 |
| 1578 | Average weekly champagne plus white wine intake | Number of glasses (125ml) | 2.37 ± 3.70 |
| 1588 | Average weekly beer plus cider intake | Number of Pints (0.57L) | 2.90 ± 4.74 |
| 1598 | Average weekly spirits intake | Number of measures (30ml) | 1.66 ± 3.68 |
| 1608 | Average weekly fortified wine intake | Number of glasses (58ml) | 0.16 ± 0.60 |
| 1618 | Alcohol usually taken with meals | 0 = No, 1 = Yes. | 20619, 39162  (34.5%, 65.5%) |
| 20117 | Alcohol drinker status | 0 = Never, 1 = Previous, 2 = Current. | 3782, 4162, 108119  (3.3%, 3.6%, 93.2%) |
| alc_combined | Average weekly alcohol intake | ml | 233.51 ± 220.41 |
| 1259 | Smoking/smokers in household | 0 = No, 1 = Yes, one household member smokes, 2 = Yes, more than one household member smokes | 94317, 9159, 1181  (90.1%, 8.8%, 1.1%) |
| 1269 | Exposure to tobacco smoke at home | Hours exposed | 0.55 ± 4.54 |
| 1279 | Exposure to tobacco smoke outside home | Hours exposed | 0.47 ± 2.28 |
| 20116 | Smoking status | 0 = Never, 1 = Previous, 2 = Current | 62062, 39773, 13992  (53.6%, 34.3%, 12.1%) |
| 20160 | Ever smoked | 0 = No, 1 = Yes | 46661, 61397  (43.2%, 56.8%) |
| 20161 | Pack years of smoking | Years | 27.65 ± 19.10 |
| 20162 | Pack years adult smoking as proportion of life span exposed to smoking | Units | 0.65 ± 0.42 |
| 806 | Job involves mainly walking or standing | 1 = Never/rarely, 2 = Sometimes, 3 = Usually, 4 = Always | 22963, 20099, 9609, 12881  (35%, 30.7%, 14.7%, 19.7%) |
| 864 | Number of days/week walked 10± minutes | Days a week | 5.38 ± 1.94 |
| 874 | Duration of walks | Minutes/day | 58.75 ± 65.00 |
| 884 | Number of days/week of moderate physical activity 10± minutes | Days per week | 3.62 ± 2.34 |
| 894 | Duration of moderate activity | Minutes/day | 63.42 ± 65.11 |
| 904 | Number of days/week of vigorous physical activity 10± minutes | Days a week | 1.81 ± 1.95 |
| 914 | Duration of vigorous activity | Minutes/day | 42.43 ± 37.11 |
| 924 | Usual walking pace | 1 = Slow pace, 2 = Steady average pace, 3 = Brisk pace | 9624, 61317, 44584  (8.3%, 53.1%, 38.6%) |
| 943 | Frequency of stair climbing in last 4 weeks | 0 = none, 1 = 1-5 times a day, 2 = 6 - 10 times a day, 3 = 11-15 times a day, 4 = 16-20 times a day, 5 = More than 20 times a day | 11075, 23059, 42275, 21331, 9883, 7621  (9.6%, 20%, 36.7%, 18.5%, 8.6%, 6.6%) |
| 971 | Frequency of walking for pleasure in last 4 weeks | 1 = Once in the last 4 weeks, 2 = 2-3 times in the last 4 weeks, 3 = 2-3 times a week , 4-5 times a week, 6 = Every day | 6201, 23839, 14231, 18847, 9149, 10049  (7.5%, 29%, 17.3%, 22.9%, 11.1%, 12.2%) |
| 981 | Duration walking for pleasure | 1 = Less than 15 minutes, 2 = Between 15 and 30 minutes, 3 = Between 30 minutes and 1 hour, 4 = Between 1 anbd 1.5 hours, 5 = Between 1.5 and 2 hours, 6 = Between 2 and 3 hours | 1359, 17181, 29942, 15162, 7444, 5500, 5677  (1.7%, 20.9%, 36.4%, 18.4%, 9%, 6.7%, 6.9%) |
| 1011 | Frequency of light DIY in last 4 weeks | 1 = Once in the last 4 weeks, 2 = 2-3 times in the last 4 weeks, 3 = 2-3 times a week , 4-5 times a week, 6 = Every day | 5575, 16980, 12091, 15438, 5280, 3322 (9.5%, 28.9%, 20.6%, 26.3%, 9%, 5.7%) |
| 1021 | Duration of light DIY | 1 = Less than 15 minutes, 2 = Between 15 and 30 minutes, 3 = Between 30 minutes and 1 hour, 4 = Between 1 anbd 1.5 hours, 5 = Between 1.5 and 2 hours, 6 = Between 2 and 3 hours | 3205, 13805, 18480, 9794, 5947, 4256, 2899 (5.5%, 23.6%, 31.7%, 16.8%, 10.2%, 7.3%, 5%) |
| 1070 | Time spent watching television (TV) | Hours/day | 2.95 ± 1.51 |
| 1080 | Time spent using computer | Hours/day | 1.16 ± 1.30 |
| 1090 | Time spent driving | Hours/day | 1.16 ± 1.23 |
| 1110 | Length of mobile phone use | 0 = Never used mobile phone at least once per week, 1 = One year or less, 2 = Two to four years, 3 = Five to eight years, 4 More than eight years | 17504, 2927, 19299, 34923, 40112  (15.3%, 2.6%, 16.8%, 30.4%, 35%) |
| 1120 | Weekly usage of mobile phone in last 3 months | 0 = Less than 5 mins, 1 = 5-29 mins, 2 = 30-59 mins, 3 = 1-3 hours, 4 = 4-6 hours, 5 = More than 6 hours | 21128, 38209, 16588, 13474, 3870, 4041  (21.7%, 39.3%, 17%, 13.8%, 4%, 4.2%) |
| 2237 | Plays computer games | 0 = Never/rarely, 1 = Sometimes, 2 = Often | 90827, 21130, 4124  (78.2%, 18.2%, 3.6%) |
| 2624 | Frequency of heavy DIY in last 4 weeks | 1 = Once in the last 4 weeks, 2 = 2-3 times in the last 4 weeks, 3 = 2-3 times a week , 4-5 times a week, 6 = Every day | 10658, 15644, 11437, 7316, 1839, 780  (22.4%, 32.8%, 24%, 15.3%, 3.9%, 1.6%) |
| 2634 | Duration of heavy DIY | 1 = Less than 15 minutes, 2 = Between 15 and 30 minutes, 3 = Between 30 minutes and 1 hour, 4 = Between 1 anbd 1.5 hours, 5 = Between 1.5 and 2 hours, 6 = Between 2 and 3 hours | 2980, 8432, 13097, 8476, 5676, 4776, 4315  (6.2%, 17.7%, 27.4%, 17.8%, 11.9%, 10%, 9%) |
| 189 | Townsend deprivation index at recruitment | Index | -1.49 ± 2.99 |
| 680 | Own or rent accommodation lived in | 1 = Own outright, 2 = Own with mortgage, 3 = Rent, from private landlord or letting agency, 4 = Rent, from local authority, local council, housing association, | 62755, 41197, 3027, 6700  (55.2%, 36.2%, 2.7%, 5.9%) |
| 699 | Length of time at current address | Years | 18.02 ± 11.52 |
| 709 | Number in household | People | 2.36 ± 1.24 |
| 728 | Number of vehicles in household | 1 = none, 2 = One, 3 = Two, 4 = Three, 5 = Four or more | 9137, 48346, 44709, 10041, 3211  (7.9%, 41.9%, 38.7%, 8.7%, 2.8%) |
| 738 | Average total household income before tax | 1 = less than 18.000, 18.000 - 30.999, 3 = 31.000 to 51.999, 4 = 52.000 - 100.00, 5 = Greater than 100.000 | 23029, 26239, 26467, 19725, 4961  (22.9%, 26.1%, 26.4%, 19.6%, 4.9%) |
| 757 | Time employed in main current job | Years | 13.73 ± 10.37 |
| 767 | Length of working week for main job | Hours | 35.03 ± 12.11 |
| 777 | Frequency of travelling from home to job workplace | Times | 4.26 ± 1.99 |
| 796 | Distance between home and job workplace | Miles | 14.37 ± 89.44 |
| 816 | Job involves heavy manual or physical work | 1 = Never/rarely, 2 = Sometimes, 3 = Usually, 4 = Always | 42159, 14210, 4692, 4496  (64.3%, 21.7%, 7.2%, 6.9%) |
| 826 | Job involves shift work | 1 = Never/rarely, 2 = Sometimes, 3 = Usually, 4 = Always | 54395, 4788, 1307, 4998  (83.1%, 7.3%, 2%, 7.6%) |
| 845 | Age completed full time education | Years | 16.49 ± 1.85 |
| 2405 | Number of children fathered | Number | 1.75 ± 1.17 |
| 4537 | Work/job satisfaction | 1= Extremely happy, 2 = Very happy, 3 = Moderately happy, 4 = Moderately unhappy, 5 = Very unhappy, 6 = Extremely unhappy | 2481, 10410, 12587, 1976, 545, 243, 14214  (5.8%, 24.5%, 29.6%, 4.7%, 1.3%, 0.6%, 33.5%) |
| 4581 | Financial situation satisfaction | 1= Extremely happy, 2 = Very happy, 3 = Moderately happy, 4 = Moderately unhappy, 5 = Very unhappy, 6 = Extremely unhappy, 7 = I am not employed | 3424, 14502, 19113, 3470, 1268, 692  (8.1%, 34.1%, 45%, 8.2%, 3%, 1.6%) |
| 6138 | Qualifications | 1 = College or University degree, 2 = A levels/AS levels or equivalent, 3 = O levels/GCSE or equivalent, 4 = CSEs or equivalent, 5 = NVQ or HND or HNC or equivalent, 6 = Other professional qualifications eg: nursing, teaching | 11171, 3066, 21901, 7962, 17190, 32757  (11.9%, 3.3%, 23.3%, 8.5%, 18.3%, 34.8%) |
| 1031 | Frequency of friend/family visits | 1 = Almost daily, 2 = 2-4 times a week, 3 = About once a week, 4 = About once a month, 5 = Once every few months, 6 = Never or almost never, 7 = No friends/family outside household | 13935, 36345, 40938, 15067, 7369, 1719, 236  (12.1%, 31.4%, 35.4%, 13%, 6.4%, 1.5%, 0.2%) |
| 1100 | Drive faster than motorway speed limit | 1 = Never/rarely, 2 = Sometimes, 3 = Often, 4 = Most of the time, 5 = Do not drive on the motorway | 47026, 42384, 11030, 4829, 7769  (41.6%, 37.5%, 9.8%, 4.3%, 6.9%) |
| 1200 | Sleeplessness / insomnia | 1 = Rarely, 2 = Sometimes, 3 = Usually | 27626, 54939, 33489  (23.8%, 47.3%, 28.9%) |
| 1920 | Mood swings | 0 = No, 1 = Yes | 62145, 51320  (54.8%, 45.2%) |
| 1930 | Miserableness | 0 = No, 1 = Yes | 65374, 48918  (57.2%, 42.8%) |
| 1940 | Irritability | 0 = No, 1 = Yes | 79793, 31426  (71.7%, 28.3%) |
| 1950 | Sensitivity / hurt feelings | 0 = No, 1 = Yes | 50556, 62438  (44.7%, 55.3%) |
| 1960 | Fed-up feelings | 0 = No, 1 = Yes. | 67233, 46708  (59%, 41%) |
| 1970 | Nervous feelings | 0 = No, 1 = Yes | 87038, 26322  (76.8%, 23.2%) |
| 1980 | Worrier / anxious feelings | 0 = No, 1 = Yes | 49867, 63417  (44%, 56%) |
| 1990 | Tense / highly strung | 0 = No, 1 = Yes | 93648, 19136  (83%, 17%) |
| 2000 | Worry too long after embarrassment | 0 = No, 1 = Yes | 58895, 52658  (52.8%, 47.2%) |
| 2010 | Suffer from nerves | 0 = No, 1 = Yes | 88436, 23627  (78.9%, 21.1%) |
| 2020 | Loneliness, isolation | 0 = No, 1 = Yes | 93917, 20569  (82%, 18%) |
| 2030 | Guilty feelings | 0 = No, 1 = Yes | 81478, 31783  (71.9%, 28.1%) |
| 2040 | Risk taking | 0 = No, 1 = Yes | 83678, 28585  (74.5%, 25.5%) |
| 2050 | Frequency of depressed mood in last 2 weeks | 1 = Not at all, 2 = Several days, 3 = More than half the days, 4 = Nearly every day | 85712, 20545, 3051, 2058  (77%, 18.4%, 2.7%, 1.8%) |
| 2060 | Frequency of unenthusiasm / disinterest in last 2 weeks | 1 = Not at all, 2 = Several days, 3 = More than half the days, 4 = Nearly every day | 89127, 18261, 2937, 2158  (79.2%, 16.2%, 2.6%, 1.9%) |
| 2070 | Frequency of tenseness / restlessness in last 2 weeks | 1 = Not at all, 2 = Several days, 3 = More than half the days, 4 = Nearly every day | 82868, 24441, 2829, 1954  (73.9%, 21.8%, 2.5%, 1.7%) |
| 2090 | Seen doctor (GP) for nerves, anxiety, tension or depression | 0 = No, 1 = Yes | 75430, 40028  (65.3%, 34.7%) |
| 2100 | Seen a psychiatrist for nerves, anxiety, tension or depression | 0 = No, 1 = Yes | 102447, 13264  (88.5%, 11.5%) |
| 2110 | Able to confide | 0 = Never or almost never, 1 = Once every few months, 2 = About once a month, 3 = About once a week, 4 = 2-4 times a week, 5 = Almost daily | 17114, 6083, 5874, 11990, 10528, 61160  (15.2%, 5.4%, 5.2%, 10.6%, 9.3%, 54.2%) |
| 4526 | Happiness | 1 = Extremely happy, 2 = very happy, 3 = Moderately happy, 4 = Moderately unhappy, 5 = Very unhappy, 6 = Extremely unhappy | 2497, 16894, 21230, 1539, 252, 85  (5.9%, 39.8%, 50%, 3.6%, 0.6%, 0.2%) |
| 4559 | Family relationship satisfaction | 1 = Extremely happy, 2 = very happy, 3 = Moderately happy, 4 = Moderately unhappy, 5 = Very unhappy, 6 = Extremely unhappy | 8794, 19181, 11709, 1791, 528, 238  (20.8%, 45.4%, 27.7%, 4.2%, 1.2%, 0.6%) |
| 4570 | Friendships satisfaction | 1 = Extremely happy, 2 = very happy, 3 = Moderately happy, 4 = Moderately unhappy, 5 = Very unhappy, 6 = Extremely unhappy | 5738, 22209, 12957, 1045, 155, 65  (13.6%, 52.7%, 30.7%, 2.5%, 0.4%, 0.2%) |
| 4598 | Ever depressed for a whole week | 0 = No, 1 = Yes | 19634, 22253  (46.9%, 53.1%) |
| 4609 | Longest period of depression | Weeks | 13.18 ± 20.97 |
| 4620 | Number of depression episodes | Periods | 3.86 ± 6.42 |
| 4631 | Ever unenthusiastic/disinterested for a whole week | 0 = No, 1 = Yes | 26069, 14948  (63.6%, 36.4%) |
| 4642 | Ever manic/hyper for 2 days | 0 = No, 1 = Yes | 39392, 2279  (94.5%, 5.5%) |
| 4653 | Ever highly irritable/argumentative for 2 days | 0 = No, 1 = Yes | 34499, 7287  (82.6%, 17.4%) |
| 20126 | Bipolar and major depression status_recoded | 0 = No Bipolar or Depression, 1 = Bipolar or depression | 19927, 7393  (72.9%, 27.1%) |
| 20127 | Neuroticism score | Score between 1 and 13 | 4.10 ± 3.26 |
| 137 | Number of treatments/ medications taken | Number | 2.55 ± 2.72 |
| 1160 | Sleep duration | Hours/day | 7.17 ± 1.10 |
| 1170 | Getting up in morning | 1= Not at all easy, 2 = Not very easy, 3 = Fairly easy, 4 = Very easy | 4383, 15495, 57433, 38686  (3.8%, 13.4%, 49.5%, 33.4%) |
| 1180 | Morning/evening person (chronotype) | 1= Definitely a 'morning' person, 2= More a 'morning' person than 'evening' person, 3 = More an 'evening' person than a 'morning' person, 4 = Definitely an 'evening' person | 27878, 37068, 29747, 9361  (26.8%, 35.6%, 28.6%, 9%) |
| 1190 | Nap during day | 1= Never/rarely, 2 = sometimes, 3 = Usually | 64807, 44858, 6433  (55.8%, 38.6%, 5.5%) |
| 1210 | Snoring | 1 = Yes, 2 = No | 41283, 66877  (38.2%, 61.8%) |
| 1220 | Daytime dozing / sleeping (narcolepsy) | 0 = Never/rarely, 1 = Sometimes, 2 = Often | 88834, 23789, 3121, 9  (76.7%, 20.6%, 2.7%, 0%) |
| 2080 | Frequency of tiredness / lethargy in last 2 weeks | 1 = Not at all, 2 = Several days, 3 = More than half the days, 4 = Nearly every day | 53392, 45666, 6642, 7154  (47.3%, 40.5%, 5.9%, 6.3%) |
| 2375 | Relative age of first facial hair | 1 = Younger than average, 2 = About average age, 3 = Older than average | 3567, 42818, 6893  (6.7%, 80.4%, 12.9%) |
| 2385 | Relative age voice broke | Years | 2.02 ± 0.32 |
| 2714 | Age when periods started (menarche) | Years | 12.94 ± 1.61 |
| 2724 | Had menopause | 0 = No, 1 = Yes, 2 = Not sure - had a hysterectomy, 3 = Not sure - other reason | 12998, 38238  (25.4%, 74.6%) |
| 2734 | Number of live births | Number of live births | 1.79 ± 1.13 |
| 2744 | Birth weight of first child | Pounds | 6.99 ± 1.20 |
| 2754 | Age at first live birth | Years | 25.16 ± 4.56 |
| 2764 | Age at last live birth | Years | 29.98 ± 4.82 |
| 2784 | Ever taken oral contraceptive pill | 0 = No, 1 = Yes. | 10583, 50456  (17.3%, 82.7%) |
| 2794 | Age started oral contraceptive pill | Years | 21.36 ± 4.58 |
| 2804 | Age when last used oral contraceptive pill | Years | 31.85 ± 7.55 |
| 2814 | Ever used hormone-replacement therapy (HRT) | 0 = No, 1 = Yes | 36529, 24452  (59.9%, 40.1%) |
| 3536 | Age started hormone-replacement therapy (HRT) | Years | 47.20 ± 5.48 |
| 4080 | Systolic blood pressure, automated reading | mmHg | 136.52 ± 18.71 |
| 20022 | Birth weight | Kg | 3.32 ± 0.67 |
| 24024 | Average 24-hour sound level of noise pollution | dB | 56.00 ± 4.22 |

Quantitative variables are described by average ± standard deviation. Ordinal variables are described by the number and percentage of individuals in each category.
