# Supplementary material for: Gene-environment interaction study for BMI reveals interactions between genetic factors and physical activity, alcohol consumption and socioeconomic status
Source: PLoS Genet. 2017 Sep 5;13(9):e1006977. doi: 10.1371/journal.pgen.1006977 (PMC5600404; doi:10.1371/journal.pgen.1006977)
Supplement: S2 Table — Three SNPs deviated from HWE and were excluded from further analysis. HWE: p-values from tests for deviation from hardy Weinberg equilibrium. β: estimated β-values from linear regression models. β_se: standard error for estimated beta-values. p: p-values for association tests. *p-adj: p-values corrected for multiple testing using the Bonferroni method. Power indicates the power to replicate, with Bonferroni adjusted p-value < 0.05, the in UK biobank considering the effect size and allele frequency of each SNP in GIANT. (DOCX) [file pgen.1006977.s005.docx]

**S2 Table. Results from tests for deviation from Hardy Weinberg equilibrium (HWE) and association with BMI in the UK Biobank cohort.**

| RSID | chr | **position** | **proximal gene** | **HWE** | ***β*** | ***β*_se** | ***p*** | ***p*-adj^*^** | ***Power*** |
| --- | --- | --- | --- | --- | --- | --- | --- | --- | --- |
| rs1558902 | 16 | 52361075 | *FTO* | 5.50E-01 | 7.62E-02 | 3.80E-03 | 4.49E-89 | 4.22E-87 | 1 |
| rs13021737 | 2 | 622348 | *TMEM18* | 1.85E-01 | 5.62E-02 | 4.97E-03 | 1.27E-29 | 1.19E-27 | 1 |
| rs543874 | 1 | 176156103 | *SEC16B* | 2.50E-01 | 5.15E-02 | 4.59E-03 | 3.25E-29 | 3.05E-27 | 1 |
| rs6567160 | 18 | 55980115 | *MC4R* | 4.73E-01 | 4.92E-02 | 4.39E-03 | 3.99E-29 | 3.75E-27 | 1 |
| rs10182181 | 2 | 25003800 | *ADCY3* | 5.44E-01 | 3.32E-02 | 3.74E-03 | 7.37E-19 | 6.93E-17 | 1 |
| rs3817334 | 11 | 47607569 | *MTCH2* | 3.68E-01 | 3.09E-02 | 3.78E-03 | 2.71E-16 | 2.55E-14 | 1 |
| rs10938397 | 4 | 44877284 | *GNPDA2* | 4.17E-02 | 2.90E-02 | 3.76E-03 | 1.13E-14 | 1.06E-12 | 1 |
| rs11030104 | 11 | 27641093 | *BDNF* | 1.50E-01 | -3.52E-02 | 4.63E-03 | 3.20E-14 | 3.01E-12 | 1 |
| rs7138803 | 12 | 48533735 | *BCDIN3D* | 1.71E-01 | 2.92E-02 | 3.85E-03 | 3.32E-14 | 3.12E-12 | 1 |
| rs2207139 | 6 | 50953449 | *TFAP2B* | 1.80E-01 | 3.68E-02 | 4.94E-03 | 1.01E-13 | 9.54E-12 | 1 |
| rs3101336 | 1 | 72523773 | *NEGR1* | 6.73E-01 | 2.74E-02 | 3.80E-03 | 5.32E-13 | 5.00E-11 | 1 |
| rs2287019 | 19 | 50894012 | *QPCTL* | 5.82E-02 | -3.46E-02 | 4.86E-03 | 1.14E-12 | 1.07E-10 | 1 |
| rs2112347 | 5 | 75050998 | *POC5* | 6.94E-02 | -2.74E-02 | 3.86E-03 | 1.43E-12 | 1.35E-10 | 1 |
| rs16951275 | 15 | 65864222 | *MAP2K5* | 2.86E-01 | -3.06E-02 | 4.41E-03 | 3.76E-12 | 3.53E-10 | 1 |
| rs3888190 | 16 | 28796987 | *ATP2A1* | 3.83E-01 | 2.65E-02 | 3.81E-03 | 3.99E-12 | 3.75E-10 | 1 |
| rs2365389 | 3 | 61211502 | *FHIT* | 3.63E-01 | -2.62E-02 | 3.81E-03 | 6.40E-12 | 6.01E-10 | 0.9 |
| rs205262 | 6 | 34671142 | *C6orf106* | 4.81E-01 | 2.84E-02 | 4.20E-03 | 1.39E-11 | 1.30E-09 | 0.82 |
| rs3810291 | 19 | 52260843 | *ZC3H4* | 2.85E-02 | 2.61E-02 | 3.98E-03 | 5.40E-11 | 5.07E-09 | 1 |
| rs1808579 | 18 | 19358886 | *C18orf8* | 1.14E-01 | -2.43E-02 | 3.73E-03 | 7.16E-11 | 6.73E-09 | 0.63 |
| rs13107325 | 4 | 103407732 | *SLC39A8* | 1.66E-01 | 4.47E-02 | 7.18E-03 | 4.81E-10 | 4.52E-08 | 0.99 |
| rs12446632 | 16 | 19842890 | *GPRC5B* | 7.41E-01 | -3.26E-02 | 5.35E-03 | 1.18E-09 | 1.11E-07 | 1 |
| rs2176598 | 11 | 43820854 | *HSD17B12* | 5.41E-02 | -2.52E-02 | 4.29E-03 | 4.26E-09 | 4.00E-07 | 0.76 |
| rs1516725 | 3 | 187306698 | *ETV5* | 3.27E-01 | 3.17E-02 | 5.40E-03 | 4.43E-09 | 4.17E-07 | 1 |
| rs12429545 | 13 | 53000207 | *OLFM4* | 6.40E-01 | 3.27E-02 | 5.59E-03 | 5.09E-09 | 4.78E-07 | 0.95 |
| rs4256980 | 11 | 8630515 | *TRIM66* | 4.39E-01 | 2.26E-02 | 3.90E-03 | 6.67E-09 | 6.27E-07 | 0.92 |
| rs7903146 | 10 | 114748339 | *TCF7L2* | 8.43E-01 | -2.31E-02 | 4.09E-03 | 1.76E-08 | 1.66E-06 | 0.94 |
| rs6091540 | 20 | 50521269 | *ZFP64* | 2.38E-02 | -2.30E-02 | 4.09E-03 | 1.82E-08 | 1.71E-06 | 0.81 |
| rs10968576 | 9 | 28404339 | *LINGO2* | 5.63E-01 | 2.21E-02 | 4.00E-03 | 3.14E-08 | 2.95E-06 | 0.98 |
| rs7164727 | 15 | 70881044 | *LOC100287559* | 8.05E-02 | 2.18E-02 | 3.95E-03 | 3.76E-08 | 3.53E-06 | 0.78 |
| rs1167827 | 7 | 75001105 | *HIP1* | 4.23E-03 | 2.07E-02 | 3.77E-03 | 4.16E-08 | 3.91E-06 | 0.91 |
| rs17724992 | 19 | 18315825 | *PGPEP1* | 1.41E-01 | -2.19E-02 | 4.22E-03 | 2.18E-07 | 2.05E-05 | 0.78 |
| rs17024393 | 1 | 109956211 | *GNAT2* | 4.28E-01 | 6.02E-02 | 1.17E-02 | 2.53E-07 | 2.38E-05 | 0.99 |
| rs751414 | 6 | 40382291 | *LFRN2* | 3.72E-01 | 2.07E-02 | 4.14E-03 | 5.24E-07 | 4.93E-05 | 0.72 |
| rs1016287 | 2 | 59159129 | *LINC01122* | 4.80E-01 | -1.99E-02 | 4.07E-03 | 9.75E-07 | 9.17E-05 | 0.95 |
| rs4787491 | 16 | 29922838 | *INO80E* | 3.88E-01 | 1.82E-02 | 3.73E-03 | 9.89E-07 | 9.30E-05 | 0.61 |
| rs11583200 | 1 | 50332407 | *ELAVL4* | 2.14E-03 | -1.87E-02 | 3.83E-03 | 1.08E-06 | 1.01E-04 | 0.76 |
| rs11165643 | 1 | 96696685 | *PTBP2* | 2.91E-02 | 1.84E-02 | 3.79E-03 | 1.25E-06 | 1.17E-04 | 0.97 |
| rs2820292 | 1 | 200050910 | *NAV1* | 1.68E-01 | 1.79E-02 | 3.74E-03 | 1.77E-06 | 1.66E-04 | 0.78 |
| rs1528435 | 2 | 181259207 | *UBE2E3* | 2.96E-01 | 1.80E-02 | 3.83E-03 | 2.59E-06 | 2.43E-04 | 0.83 |
| rs12940622 | 17 | 76230166 | *RPTOR* | 6.81E-01 | -1.75E-02 | 3.74E-03 | 2.93E-06 | 2.76E-04 | 0.71 |
| rs4740619 | 9 | 15624326 | *C9orf93* | 8.30E-01 | -1.74E-02 | 3.73E-03 | 3.04E-06 | 2.86E-04 | 0.69 |
| rs10132280 | 14 | 24998019 | *STXBP6* | 3.86E-01 | -1.91E-02 | 4.11E-03 | 3.29E-06 | 3.09E-04 | 0.96 |
| rs13078960 | 3 | 85890280 | *CADM2* | 8.40E-01 | 2.13E-02 | 4.69E-03 | 5.49E-06 | 5.16E-04 | 0.98 |
| rs29941 | 19 | 39001372 | *KCTD15* | 4.75E-02 | 1.81E-02 | 3.98E-03 | 5.63E-06 | 5.29E-04 | 0.73 |
| rs7599312 | 2 | 213121476 | *ERBB4* | 5.44E-01 | -1.93E-02 | 4.26E-03 | 5.95E-06 | 5.59E-04 | 0.83 |
| rs2650492 | 16 | 28240912 | *SBK1* | 5.16E-01 | 1.86E-02 | 4.15E-03 | 7.00E-06 | 6.58E-04 | 0.87 |
| rs11191560 | 10 | 104859028 | *NT5C2* | 6.00E-02 | 3.05E-02 | 6.86E-03 | 8.59E-06 | 8.07E-04 | 0.76 |
| rs11057405 | 12 | 121347850 | *CLIP1* | 8.63E-02 | -2.68E-02 | 6.14E-03 | 1.28E-05 | 1.20E-03 | 0.76 |
| rs12566985 | 1 | 74774781 | *FPGT-TNNI3K* | 3.56E-01 | -1.61E-02 | 3.77E-03 | 1.86E-05 | 1.75E-03 | 1 |
| rs10733682 | 9 | 128500735 | *LMX1B* | 7.83E-01 | -1.63E-02 | 3.81E-03 | 1.91E-05 | 1.80E-03 | 0.85 |
| rs7141420 | 14 | 78969207 | *NRXN3* | 3.38E-01 | 1.59E-02 | 3.75E-03 | 2.34E-05 | 2.20E-03 | 0.98 |
| rs13191362 | 6 | 162953340 | *PARK2* | 2.52E-01 | -2.37E-02 | 5.67E-03 | 2.85E-05 | 2.68E-03 | 0.88 |
| rs2245368 | 7 | 76446079 | *PMS2L11* | 2.21E-01 | -2.03E-02 | 4.91E-03 | 3.60E-05 | 3.38E-03 | 0.95 |
| rs977747 | 1 | 47457264 | *TAL1* | 2.00E-01 | -1.56E-02 | 3.79E-03 | 3.90E-05 | 3.67E-03 | 0.71 |
| rs2836754 | 21 | 39213610 | *ETS2* | 7.75E-01 | 1.58E-02 | 3.86E-03 | 4.20E-05 | 3.95E-03 | 0.68 |
| rs12885454 | 14 | 28806589 | *PRKD1* | 6.28E-01 | -1.59E-02 | 3.90E-03 | 4.53E-05 | 4.26E-03 | 0.79 |
| rs9400239 | 6 | 109084356 | *FOXO3* | 6.26E-01 | 1.66E-02 | 4.09E-03 | 4.87E-05 | 4.57E-03 | 0.65 |
| rs657452 | 1 | 49362434 | *AGBL4* | 1.33E-02 | -1.51E-02 | 3.81E-03 | 7.18E-05 | 6.75E-03 | 0.98 |
| rs1441264 | 13 | 78478920 | *MIR548A2* | 8.78E-02 | 1.51E-02 | 3.81E-03 | 7.30E-05 | 6.86E-03 | 0.56 |
| rs12401738 | 1 | 78219349 | *FUBP1* | 1.58E-01 | 1.53E-02 | 3.88E-03 | 7.64E-05 | 7.19E-03 | 0.88 |
| rs16851483 | 3 | 142758126 | *RASA2* | 5.18E-01 | 2.86E-02 | 7.46E-03 | 1.23E-04 | 1.15E-02 | 0.98 |
| rs7243357 | 18 | 55034299 | *GRP* | 4.37E-02 | -1.70E-02 | 4.88E-03 | 4.75E-04 | 4.46E-02 | 0.75 |
| rs17405819 | 8 | 76969139 | *HNF4G* | 4.13E-01 | -1.42E-02 | 4.08E-03 | 5.22E-04 | 4.91E-02 | 0.9 |
| rs16907751 | 8 | 81538012 | *ZBTB10* | 6.61E-02 | -2.10E-02 | 6.29E-03 | 8.57E-04 | 8.06E-02 | 0.87 |
| rs7239883 | 18 | 38401669 | *LOC284260* | 2.87E-01 | -1.25E-02 | 3.83E-03 | 1.16E-03 | 1.09E-01 | 0.51 |
| rs11688816 | 2 | 62906552 | *EHBP1* | 1.17E-01 | -1.22E-02 | 3.77E-03 | 1.21E-03 | 1.13E-01 | 0.52 |
| rs7715256 | 5 | 153518086 | *GALNT10* | 6.36E-01 | -1.20E-02 | 3.75E-03 | 1.35E-03 | 1.27E-01 | 0.71 |
| rs1460676 | 2 | 164275935 | *FIGN* | 3.40E-01 | 1.60E-02 | 5.10E-03 | 1.66E-03 | 1.56E-01 | 0.72 |
| rs17094222 | 10 | 102385430 | *HIF1AN* | 7.05E-01 | 1.46E-02 | 4.64E-03 | 1.69E-03 | 1.59E-01 | 0.91 |
| rs7899106 | 10 | 87400884 | *GRID1* | 4.75E-02 | 2.68E-02 | 8.57E-03 | 1.78E-03 | 1.67E-01 | 0.78 |
| rs6804842 | 3 | 25081441 | *RARB* | 1.97E-01 | 1.13E-02 | 3.77E-03 | 2.70E-03 | 2.53E-01 | 0.73 |
| rs2080454 | 16 | 47620091 | *CBLN1* | 3.03E-01 | -1.10E-02 | 3.86E-03 | 4.24E-03 | 3.99E-01 | 0.74 |
| rs3849570 | 3 | 81874802 | *GBE1* | 6.14E-01 | 1.11E-02 | 3.91E-03 | 4.61E-03 | 4.33E-01 | 0.79 |
| rs758747 | 16 | 3567359 | *NLRC3* | 8.61E-02 | 1.13E-02 | 4.18E-03 | 6.63E-03 | 6.24E-01 | 0.91 |
| rs9540493 | 13 | 65103705 | *MIR548X2* | 9.42E-01 | -1.01E-02 | 3.75E-03 | 7.26E-03 | 6.82E-01 | 0.79 |
| rs3736485 | 15 | 49535902 | *DMXL2* | 3.17E-01 | -1.00E-02 | 3.75E-03 | 7.35E-03 | 6.91E-01 | 0.7 |
| rs9581854 | 13 | 27443645 | *MTIF3* | 5.62E-02 | 1.31E-02 | 4.90E-03 | 7.38E-03 | 6.94E-01 | 0.99 |
| rs17203016 | 2 | 207963763 | *CREB1* | 4.38E-01 | 1.24E-02 | 4.72E-03 | 8.33E-03 | 7.83E-01 | 0.77 |
| rs12286929 | 11 | 114527614 | *CADM1* | 1.61E-01 | 9.14E-03 | 3.74E-03 | 1.45E-02 | 1.36E+00 | 0.97 |
| rs1928295 | 9 | 119418304 | *TLR4* | 2.12E-01 | -9.05E-03 | 3.74E-03 | 1.56E-02 | 1.47E+00 | 0.82 |
| rs9374842 | 6 | 120227364 | *LOC285762* | 4.36E-01 | 9.99E-03 | 4.42E-03 | 2.38E-02 | 2.24E+00 | 0.77 |
| rs6477694 | 9 | 110972163 | *EPB41L4B* | 8.00E-01 | -8.39E-03 | 3.90E-03 | 3.15E-02 | 2.96E+00 | 0.74 |
| rs1000940 | 17 | 5223976 | *RABEP1* | 1.27E-02 | 8.33E-03 | 4.06E-03 | 4.02E-02 | 3.78E+00 | 0.66 |
| rs11847697 | 14 | 29584863 | *PRKD1* | 7.21E-02 | 1.70E-02 | 8.71E-03 | 5.13E-02 | 4.82E+00 | 0.97 |
| rs9641123 | 7 | 93035668 | *CALCR* | 2.77E-01 | 6.75E-03 | 3.79E-03 | 7.53E-02 | 7.08E+00 | 0.78 |
| rs9914578 | 17 | 1951886 | *SMG6* | 1.20E-01 | 7.57E-03 | 4.59E-03 | 9.91E-02 | 9.32E+00 | 0.66 |
| rs13201877 | 6 | 137717234 | *IFNGR1* | 3.83E-01 | 7.41E-03 | 5.57E-03 | 1.83E-01 | 1.72E+01 | 0.62 |
| rs2176040 | 2 | 226801046 | *LOC646736* | 3.17E-01 | -4.11E-03 | 3.91E-03 | 2.93E-01 | 2.76E+01 | 0.49 |
| rs2121279 | 2 | 142759755 | *LRP1B* | 1.78E-01 | 5.84E-03 | 5.70E-03 | 3.06E-01 | 2.88E+01 | 0.72 |
| rs492400 | 2 | 219057996 | *USP37* | 1.27E-01 | -2.79E-03 | 3.76E-03 | 4.59E-01 | 4.32E+01 | 0.5 |
| rs11126666 | 2 | 26782315 | *KCNK3* | 1.33E-01 | -2.75E-03 | 4.26E-03 | 5.18E-01 | 4.87E+01 | 0.82 |
| rs2033732 | 8 | 85242264 | *RALYL* | 1.83E-01 | 2.34E-03 | 4.27E-03 | 5.84E-01 | 5.49E+01 | 0.59 |
| rs6465468 | 7 | 95007450 | *ASB4* | 3.71E-01 | 1.58E-03 | 4.09E-03 | 6.99E-01 | 6.57E+01 | 0.56 |
| rs11727676 | 4 | 145878514 | *HHIP* | 5.90E-01 | 1.34E-03 | 6.34E-03 | 8.33E-01 | 7.83E+01 | 0.94 |
| rs9925964 | 16 | 31037396 | *KAT8* | 0.00E+00 |  |  |  |  | 0.84 |
| rs2075650 | 19 | 50087459 | *TOMM40* | 0.00E+00 |  |  |  |  | 0.86 |
| rs17001654 | 4 | 77348592 | *SCARB2* | 3.84E-86 |  |  |  |  | 0.95 |

Three SNPs deviated from HWE and were excluded from further analysis. HWE: p-values from tests for deviation from hardy Weinberg equilibrium. *β:* estimated *β*-values from linear regression models. *β*_se: standard error for estimated beta-values. *p*: p-values for association tests. ^*^*p*-adj: p-values corrected for multiple testing using the Bonferroni method. Power indicates the power to replicate, with Bonferroni adjusted p-value < 0.05, the in UK biobank considering the effect size and allele frequency of each SNP in GIANT.
