# Supplementary material for: Gene-environment interaction study for BMI reveals interactions between genetic factors and physical activity, alcohol consumption and socioeconomic status
Source: PLoS Genet. 2017 Sep 5;13(9):e1006977. doi: 10.1371/journal.pgen.1006977 (PMC5600404; doi:10.1371/journal.pgen.1006977)
Supplement: S3 Table — β is the effect size estimate of the SNP, SE β is the standard error of the effect size estimate, and p is the corresponding p-value from association tests between SNPs and BMI. p#—p-values from student’s two-sample t-tests to compare means between males and females. p-adj*—p values adjusted for multiple testing using the Bonferroni method. (DOCX) [file pgen.1006977.s006.docx]

**S3 Table. Comparison of SNP-effects on BMI between male and female participants of UK Biobank.**

| **SNP** | ***Association in females*** | | | | | ***Association in males*** | | | ***Comparison between males and females*** | | |
| --- | --- | --- | --- | --- | --- | --- | --- | --- | --- | --- | --- |
|  | ***β*** | | **SE *β*** | | ***p*** | ***β2*** | **SE β3** | ***p4*** | ***p^#^*** | | ***p*-adj^*^** |
| rs9925964 | -0.107 | 0.027 | | 9.21E-05 | | -0.065 | 0.024 | 5.58E-03 | 0.25 | 1.00 | |
| rs2075650 | -0.095 | 0.041 | | 1.91E-02 | | -0.038 | 0.035 | 2.82E-01 | 0.28 | 1.00 | |
| rs17001654 | 0.022 | 0.041 | | 5.94E-01 | | 0.052 | 0.035 | 1.43E-01 | 0.58 | 1.00 | |
| rs1558902 | 0.402 | 0.030 | | 5.71E-40 | | 0.359 | 0.026 | 7.36E-42 | 0.28 | 1.00 | |
| rs13021737 | 0.304 | 0.040 | | 2.19E-14 | | 0.280 | 0.035 | 7.74E-16 | 0.65 | 1.00 | |
| rs6567160 | 0.301 | 0.035 | | 1.27E-17 | | 0.193 | 0.031 | 3.74E-10 | 0.02 | 1.00 | |
| rs543874 | 0.313 | 0.037 | | 1.14E-17 | | 0.147 | 0.032 | 4.87E-06 | 0.00 | 0.06 | |
| rs10182181 | 0.158 | 0.030 | | 1.20E-07 | | 0.146 | 0.026 | 2.06E-08 | 0.75 | 1.00 | |
| rs11030104 | -0.170 | 0.037 | | 4.84E-06 | | -0.226 | 0.032 | 3.07E-12 | 0.25 | 1.00 | |
| rs10938397 | 0.152 | 0.030 | | 5.08E-07 | | 0.126 | 0.026 | 1.59E-06 | 0.52 | 1.00 | |
| rs7138803 | 0.195 | 0.031 | | 3.15E-10 | | 0.120 | 0.027 | 7.98E-06 | 0.07 | 1.00 | |
| rs3817334 | 0.132 | 0.030 | | 1.34E-05 | | 0.141 | 0.026 | 8.59E-08 | 0.82 | 1.00 | |
| rs2287019 | -0.211 | 0.039 | | 6.20E-08 | | -0.133 | 0.034 | 8.46E-05 | 0.13 | 1.00 | |
| rs2207139 | 0.195 | 0.040 | | 1.00E-06 | | 0.161 | 0.035 | 3.07E-06 | 0.52 | 1.00 | |
| rs3101336 | 0.121 | 0.030 | | 7.38E-05 | | 0.142 | 0.026 | 8.12E-08 | 0.59 | 1.00 | |
| rs205262 | 0.164 | 0.034 | | 1.21E-06 | | 0.110 | 0.029 | 1.64E-04 | 0.23 | 1.00 | |
| rs2112347 | -0.187 | 0.031 | | 1.79E-09 | | -0.073 | 0.027 | 7.52E-03 | 0.01 | 0.53 | |
| rs2365389 | -0.173 | 0.031 | | 1.46E-08 | | -0.091 | 0.026 | 5.99E-04 | 0.04 | 1.00 | |
| rs16951275 | -0.182 | 0.036 | | 3.29E-07 | | -0.120 | 0.031 | 1.18E-04 | 0.19 | 1.00 | |
| rs1808579 | -0.103 | 0.030 | | 5.74E-04 | | -0.122 | 0.026 | 3.01E-06 | 0.64 | 1.00 | |
| rs3888190 | 0.092 | 0.031 | | 2.56E-03 | | 0.139 | 0.026 | 1.52E-07 | 0.25 | 1.00 | |
| rs3810291 | 0.135 | 0.032 | | 2.44E-05 | | 0.138 | 0.028 | 7.11E-07 | 0.95 | 1.00 | |
| rs7903146 | -0.139 | 0.033 | | 2.46E-05 | | -0.126 | 0.029 | 1.07E-05 | 0.77 | 1.00 | |
| rs13107325 | 0.228 | 0.057 | | 6.93E-05 | | 0.150 | 0.049 | 2.29E-03 | 0.30 | 1.00 | |
| rs1516725 | 0.159 | 0.043 | | 2.45E-04 | | 0.145 | 0.038 | 1.29E-04 | 0.81 | 1.00 | |
| rs2176598 | -0.135 | 0.035 | | 9.78E-05 | | -0.089 | 0.030 | 3.31E-03 | 0.32 | 1.00 | |
| rs12446632 | -0.143 | 0.043 | | 8.10E-04 | | -0.073 | 0.037 | 5.12E-02 | 0.22 | 1.00 | |
| rs4256980 | 0.127 | 0.032 | | 5.72E-05 | | 0.081 | 0.027 | 2.97E-03 | 0.27 | 1.00 | |
| rs12429545 | 0.143 | 0.045 | | 1.42E-03 | | 0.111 | 0.039 | 4.49E-03 | 0.59 | 1.00 | |
| rs10968576 | 0.128 | 0.032 | | 6.29E-05 | | 0.098 | 0.028 | 3.93E-04 | 0.48 | 1.00 | |
| rs1167827 | 0.134 | 0.030 | | 8.30E-06 | | 0.072 | 0.026 | 6.39E-03 | 0.12 | 1.00 | |
| rs7164727 | 0.114 | 0.032 | | 3.42E-04 | | 0.112 | 0.028 | 5.55E-05 | 0.96 | 1.00 | |
| rs1016287 | -0.098 | 0.033 | | 2.68E-03 | | -0.104 | 0.028 | 2.53E-04 | 0.90 | 1.00 | |
| rs6091540 | -0.068 | 0.033 | | 3.88E-02 | | -0.140 | 0.028 | 8.95E-07 | 0.10 | 1.00 | |
| rs751414 | 0.119 | 0.033 | | 3.08E-04 | | 0.091 | 0.029 | 1.52E-03 | 0.53 | 1.00 | |
| rs29941 | 0.121 | 0.032 | | 1.41E-04 | | 0.070 | 0.028 | 1.19E-02 | 0.22 | 1.00 | |
| rs2820292 | 0.075 | 0.030 | | 1.27E-02 | | 0.118 | 0.026 | 6.65E-06 | 0.28 | 1.00 | |
| rs17024393 | 0.379 | 0.095 | | 6.16E-05 | | 0.292 | 0.082 | 3.68E-04 | 0.49 | 1.00 | |
| rs17724992 | -0.117 | 0.034 | | 5.47E-04 | | -0.101 | 0.030 | 6.53E-04 | 0.71 | 1.00 | |
| rs12940622 | -0.097 | 0.030 | | 1.32E-03 | | -0.080 | 0.026 | 2.13E-03 | 0.68 | 1.00 | |
| rs11583200 | -0.088 | 0.031 | | 4.02E-03 | | -0.087 | 0.027 | 1.10E-03 | 0.98 | 1.00 | |
| rs13078960 | 0.093 | 0.037 | | 1.25E-02 | | 0.128 | 0.033 | 8.07E-05 | 0.48 | 1.00 | |
| rs1528435 | 0.094 | 0.031 | | 2.16E-03 | | 0.033 | 0.027 | 2.11E-01 | 0.14 | 1.00 | |
| rs2245368 | -0.071 | 0.040 | | 7.35E-02 | | -0.156 | 0.035 | 7.55E-06 | 0.11 | 1.00 | |
| rs4740619 | -0.096 | 0.030 | | 1.40E-03 | | -0.044 | 0.026 | 9.18E-02 | 0.19 | 1.00 | |
| rs4787491 | 0.068 | 0.030 | | 2.39E-02 | | 0.101 | 0.026 | 1.01E-04 | 0.40 | 1.00 | |
| rs7599312 | -0.069 | 0.034 | | 4.16E-02 | | -0.125 | 0.030 | 2.43E-05 | 0.22 | 1.00 | |
| rs10132280 | -0.065 | 0.033 | | 4.93E-02 | | -0.117 | 0.029 | 4.76E-05 | 0.24 | 1.00 | |
| rs11057405 | -0.169 | 0.048 | | 4.53E-04 | | -0.106 | 0.042 | 1.20E-02 | 0.32 | 1.00 | |
| rs12885454 | -0.082 | 0.031 | | 8.60E-03 | | -0.077 | 0.027 | 4.72E-03 | 0.90 | 1.00 | |
| rs13191362 | -0.119 | 0.045 | | 7.83E-03 | | -0.116 | 0.040 | 3.30E-03 | 0.97 | 1.00 | |
| rs11165643 | 0.083 | 0.030 | | 6.04E-03 | | 0.062 | 0.026 | 1.95E-02 | 0.60 | 1.00 | |
| rs977747 | -0.057 | 0.030 | | 5.91E-02 | | -0.091 | 0.026 | 5.59E-04 | 0.40 | 1.00 | |
| rs7141420 | 0.071 | 0.030 | | 1.83E-02 | | 0.100 | 0.026 | 1.47E-04 | 0.47 | 1.00 | |
| rs11191560 | 0.061 | 0.056 | | 2.80E-01 | | 0.196 | 0.049 | 5.49E-05 | 0.07 | 1.00 | |
| rs9400239 | 0.084 | 0.033 | | 1.11E-02 | | 0.093 | 0.029 | 1.16E-03 | 0.83 | 1.00 | |
| rs2650492 | 0.056 | 0.033 | | 9.02E-02 | | 0.112 | 0.029 | 9.33E-05 | 0.20 | 1.00 | |
| rs10733682 | -0.092 | 0.031 | | 2.66E-03 | | -0.066 | 0.027 | 1.32E-02 | 0.52 | 1.00 | |
| rs16851483 | 0.157 | 0.060 | | 8.98E-03 | | 0.130 | 0.053 | 1.33E-02 | 0.74 | 1.00 | |
| rs12566985 | -0.091 | 0.030 | | 2.68E-03 | | -0.003 | 0.026 | 9.18E-01 | 0.03 | 1.00 | |
| rs2836754 | 0.112 | 0.031 | | 3.27E-04 | | 0.043 | 0.027 | 1.09E-01 | 0.10 | 1.00 | |
| rs12401738 | 0.060 | 0.031 | | 5.13E-02 | | 0.070 | 0.027 | 9.35E-03 | 0.81 | 1.00 | |
| rs657452 | -0.067 | 0.031 | | 2.87E-02 | | -0.051 | 0.027 | 5.58E-02 | 0.70 | 1.00 | |
| rs1441264 | 0.062 | 0.030 | | 4.30E-02 | | 0.097 | 0.027 | 2.80E-04 | 0.39 | 1.00 | |
| rs17405819 | -0.090 | 0.033 | | 5.82E-03 | | -0.040 | 0.028 | 1.57E-01 | 0.25 | 1.00 | |
| rs17094222 | 0.034 | 0.037 | | 3.59E-01 | | 0.119 | 0.032 | 2.16E-04 | 0.08 | 1.00 | |
| rs7243357 | -0.048 | 0.039 | | 2.16E-01 | | -0.081 | 0.034 | 1.81E-02 | 0.53 | 1.00 | |
| rs6804842 | 0.054 | 0.030 | | 7.36E-02 | | 0.049 | 0.026 | 6.00E-02 | 0.91 | 1.00 | |
| rs7239883 | -0.074 | 0.031 | | 1.60E-02 | | -0.028 | 0.027 | 2.99E-01 | 0.26 | 1.00 | |
| rs7715256 | -0.076 | 0.030 | | 1.16E-02 | | -0.060 | 0.026 | 2.17E-02 | 0.69 | 1.00 | |
| rs16907751 | -0.147 | 0.052 | | 4.40E-03 | | -0.104 | 0.045 | 1.96E-02 | 0.53 | 1.00 | |
| rs11688816 | -0.079 | 0.030 | | 8.69E-03 | | -0.042 | 0.026 | 1.12E-01 | 0.35 | 1.00 | |
| rs9581854 | 0.078 | 0.039 | | 4.48E-02 | | 0.083 | 0.034 | 1.41E-02 | 0.92 | 1.00 | |
| rs7899106 | 0.122 | 0.068 | | 7.50E-02 | | 0.090 | 0.060 | 1.34E-01 | 0.73 | 1.00 | |
| rs1460676 | 0.103 | 0.041 | | 1.24E-02 | | 0.076 | 0.036 | 3.42E-02 | 0.62 | 1.00 | |
| rs2080454 | -0.111 | 0.031 | | 3.09E-04 | | -0.020 | 0.027 | 4.50E-01 | 0.03 | 1.00 | |
| rs17203016 | 0.064 | 0.038 | | 8.89E-02 | | 0.072 | 0.033 | 2.69E-02 | 0.87 | 1.00 | |
| rs3736485 | -0.085 | 0.030 | | 4.73E-03 | | -0.027 | 0.026 | 3.11E-01 | 0.14 | 1.00 | |
| rs12286929 | 0.035 | 0.030 | | 2.49E-01 | | 0.057 | 0.026 | 2.91E-02 | 0.57 | 1.00 | |
| rs9540493 | -0.066 | 0.030 | | 2.92E-02 | | -0.046 | 0.026 | 7.90E-02 | 0.62 | 1.00 | |
| rs758747 | 0.065 | 0.034 | | 5.19E-02 | | 0.069 | 0.029 | 1.90E-02 | 0.94 | 1.00 | |
| rs1000940 | 0.067 | 0.033 | | 3.88E-02 | | 0.048 | 0.028 | 9.26E-02 | 0.65 | 1.00 | |
| rs9374842 | 0.041 | 0.036 | | 2.48E-01 | | 0.068 | 0.031 | 2.73E-02 | 0.57 | 1.00 | |
| rs1928295 | -0.053 | 0.030 | | 8.00E-02 | | -0.024 | 0.026 | 3.66E-01 | 0.46 | 1.00 | |
| rs3849570 | 0.064 | 0.031 | | 4.03E-02 | | 0.008 | 0.027 | 7.84E-01 | 0.17 | 1.00 | |
| rs11847697 | 0.096 | 0.073 | | 1.86E-01 | | 0.017 | 0.064 | 7.93E-01 | 0.41 | 1.00 | |
| rs9641123 | 0.080 | 0.030 | | 8.35E-03 | | 0.014 | 0.026 | 5.87E-01 | 0.10 | 1.00 | |
| rs6477694 | -0.004 | 0.031 | | 9.11E-01 | | -0.057 | 0.027 | 3.75E-02 | 0.20 | 1.00 | |
| rs2176040 | -0.017 | 0.031 | | 5.93E-01 | | -0.061 | 0.027 | 2.53E-02 | 0.29 | 1.00 | |
| rs13201877 | 0.033 | 0.044 | | 4.50E-01 | | 0.021 | 0.038 | 5.91E-01 | 0.83 | 1.00 | |
| rs9914578 | 0.023 | 0.037 | | 5.33E-01 | | 0.030 | 0.032 | 3.48E-01 | 0.89 | 1.00 | |
| rs492400 | 0.014 | 0.030 | | 6.31E-01 | | -0.045 | 0.026 | 8.67E-02 | 0.14 | 1.00 | |
| rs2121279 | 0.013 | 0.045 | | 7.78E-01 | | 0.028 | 0.039 | 4.71E-01 | 0.80 | 1.00 | |
| rs2033732 | -0.008 | 0.034 | | 8.13E-01 | | 0.023 | 0.030 | 4.37E-01 | 0.49 | 1.00 | |
| rs11126666 | -0.023 | 0.034 | | 5.03E-01 | | 0.026 | 0.030 | 3.75E-01 | 0.28 | 1.00 | |
| rs11727676 | 0.074 | 0.050 | | 1.39E-01 | | -0.059 | 0.044 | 1.81E-01 | 0.05 | 1.00 | |
| rs6465468 | 0.011 | 0.032 | | 7.36E-01 | | 0.020 | 0.028 | 4.90E-01 | 0.84 | 1.00 | |

*β* is the effect size estimate of the SNP, *SE β* is the standard error of the effect size estimate, and *p* is the corresponding p-value from association tests between SNPs and BMI. *p^#^* - p-values from student’s two-sample t-tests to compare means between males and females. *p*-adj^*^ - p values adjusted for multiple testing using the Bonferroni method.
