# Supplementary material for: Gene-environment interaction study for BMI reveals interactions between genetic factors and physical activity, alcohol consumption and socioeconomic status
Source: PLoS Genet. 2017 Sep 5;13(9):e1006977. doi: 10.1371/journal.pgen.1006977 (PMC5600404; doi:10.1371/journal.pgen.1006977)
Supplement: S4 Table — N: number of individuals included in the respective analyses. E: the results, with corresponding estimates (β) and p-values (p) for the linear models testing for the effect on each lifestyle variable on BMI without including the interaction term. GSBMI × E: Results for the interaction term from linear models for association with the genetic score for BMI composed of the effects of 94 SNPs associated with BMI. β2: Estimated effect sizes of the interaction. p2: p-value for tests of the estimated effect size deviating from zero. GSBMI' × E is the genetic score for BMI excluding the FTO SNP rs1558902 with corresponding estimates (β3) and p-values (p3) for the interaction terms. (DOCX) [file pgen.1006977.s007.docx]

**S4 Table.** **Effect by, and interaction with genetic risk score for dietary habits, assessed by self-report touchscreen questionnaire.**

| **ID** | **NAME** | **N** | ***E*** | | ***GS_BMI_ × E*** | | ***GS_BMI_' × E*** | |
| --- | --- | --- | --- | --- | --- | --- | --- | --- |
|  |  |  | ***p*** | ***β*** | ***p2*** | ***β2*** | **p3** | ***β3*** |
| 1289 | Cooked vegetable intake | 111542 | 9.47E-22 | 2.14E-02 | 6.57E-01 | -6.10E-03 | 5.53E-01 | -8.71E-03 |
| 1299 | Salad / raw vegetable intake | 108682 | 8.38E-01 | -3.64E-04 | 4.50E-03 | -3.11E-02 | 4.76E-03 | -3.29E-02 |
| 1309 | Fresh fruit intake | 110667 | 1.30E-08 | 1.28E-02 | 6.75E-01 | -5.81E-03 | 3.89E-01 | -1.28E-02 |
| 1319 | Dried fruit intake | 105321 | 3.97E-206 | -7.88E-02 | 1.16E-01 | -2.50E-02 | 7.90E-02 | -2.98E-02 |
| 1329 | Oily fish intake | 115607 | 1.09E-28 | -3.55E-02 | 5.24E-01 | -1.25E-02 | 2.56E-01 | -2.38E-02 |
| 1339 | Non-oily fish intake | 115706 | 1.51E-04 | 1.43E-02 | 7.30E-01 | 7.94E-03 | 9.28E-01 | 2.22E-03 |
| 1349 | Processed meat intake | 115966 | 1.25E-300 | 1.07E-01 | 3.11E-03 | 5.22E-02 | 3.94E-02 | 3.89E-02 |
| 1359 | Poultry intake | 115955 | <2.2E-308 | 1.27E-01 | 4.88E-01 | 1.43E-02 | 6.37E-01 | 1.04E-02 |
| 1369 | Beef intake | 115736 | 4.02E-264 | 1.23E-01 | 7.08E-01 | 8.11E-03 | 8.47E-01 | -4.44E-03 |
| 1379 | Lamb/mutton intake | 115515 | 1.31E-78 | 7.88E-02 | 8.91E-02 | 4.39E-02 | 2.04E-01 | 3.49E-02 |
| 1389 | Pork intake | 115539 | 3.21E-235 | 1.35E-01 | 2.12E-02 | 5.84E-02 | 1.64E-01 | 3.76E-02 |
| 1408 | Cheese intake | 113531 | 3.35E-108 | -6.12E-02 | 5.15E-01 | -1.10E-02 | 8.84E-01 | 2.65E-03 |
| 1438 | Bread intake | 112695 | 8.69E-02 | -6.82E-04 | 9.60E-03 | 6.35E-03 | 2.44E-02 | 5.88E-03 |
| 1458 | Cereal intake | 110177 | 2.80E-81 | -2.19E-02 | 5.63E-01 | -4.07E-03 | 9.13E-01 | -8.24E-04 |
| 1478 | Salt added to food | 116126 | 3.33E-79 | 6.36E-02 | 8.20E-02 | 3.59E-02 | 4.75E-01 | 1.57E-02 |
| 1488 | Tea intake | 111506 | 2.46E-20 | -1.07E-02 | 4.13E-01 | -5.85E-03 | 1.94E-01 | -9.91E-03 |
| 1498 | Coffee intake | 107501 | 2.79E-64 | 2.54E-02 | 9.13E-01 | 1.00E-03 | 8.86E-01 | -1.40E-03 |
| 1518 | Hot drink temperature | 114928 | 6.87E-94 | 1.05E-01 | 3.86E-01 | 2.73E-02 | 1.99E-01 | 4.30E-02 |
| 1528 | Water intake | 106705 | 8.04E-34 | 1.85E-02 | 1.18E-01 | -1.46E-02 | 2.42E-01 | -1.17E-02 |
| 1548 | Variation in diet | 115853 | 0.00E+00 | 2.14E-01 | 2.13E-03 | 9.17E-02 | 1.54E-03 | 1.01E-01 |

N: number of individuals included in the respective analyses. *E*: the results, with corresponding estimates (*β)* and p-values *(p)* for the linear models testing for the effect on each lifestyle variable on BMI without including the interaction term. *GS_BMI_ × E*: Results for the interaction term from linear models for association with the genetic score for BMI composed of the effects of 94 SNPs associated with BMI. *β2*: Estimated effect sizes of the interaction. *p2:* p-value for tests of the estimated effect size deviating from zero. *GS_BMI_' × E* is the genetic score for BMI excluding the *FTO* SNP rs1558902 with corresponding estimates (*β3)* and p-values *(p3)* for the interaction terms.
