# Supplementary material for: Gene-environment interaction study for BMI reveals interactions between genetic factors and physical activity, alcohol consumption and socioeconomic status
Source: PLoS Genet. 2017 Sep 5;13(9):e1006977. doi: 10.1371/journal.pgen.1006977 (PMC5600404; doi:10.1371/journal.pgen.1006977)
Supplement: S5 Table — N: number of individuals included in the respective analyses. E: the results, with corresponding estimates (β) and p-values (p) for the linear models testing for the effect on each lifestyle variable on BMI without including the interaction term. GSBMI × E: Results for the interaction term from linear models for association with the genetic score for BMI composed of the effects of 94 SNPs associated with BMI. β2: Estimated effect sizes of the interaction. p2: p-value for tests of the estimated effect size deviating from zero. GSBMI' × E is the genetic score for BMI excluding the FTO SNP rs1558902 with corresponding estimates (β3) and p-values (p3) for the interaction terms. (DOCX) [file pgen.1006977.s008.docx]

**S5 Table.** **Effect by, and interactions between genetic risk score for BMI and smoking, and alcohol consumption habits, assessed by self-report touchscreen questionnaire.**

| **ID** | **NAME** | **N** | ***E*** | | ***GS_BMI_ × E*** | | ***GS_BMI_' × E*** | |
| --- | --- | --- | --- | --- | --- | --- | --- | --- |
|  |  |  | ***p*** | ***β*** | ***p2*** | ***β2*** | ***p3*** | ***β3*** |
| 1558 | Alcohol intake frequency. | 116063 | <2.2E-308 | 8.35E-02 | 1.87E-16 | 9.92E-02 | 4.37E-13 | 9.34E-02 |
| 1568 | Average weekly red wine intake | 81566 | 3.36E-09 | -4.12E-03 | 2.37E-04 | -1.58E-02 | 1.20E-04 | -1.77E-02 |
| 1578 | Average weekly champagne plus white wine intake | 81515 | 1.22E-04 | -3.43E-03 | 2.23E-01 | -6.69E-03 | 3.27E-01 | -5.74E-03 |
| 1588 | Average weekly beer plus cider intake | 81770 | 2.34E-63 | 1.30E-02 | 6.10E-01 | 2.42E-03 | 5.67E-01 | 2.88E-03 |
| 1598 | Average weekly spirits intake | 81730 | 1.62E-129 | 2.15E-02 | 8.42E-01 | 1.07E-03 | 8.65E-01 | 9.91E-04 |
| 1608 | Average weekly fortified wine intake | 81866 | 9.31E-04 | -1.81E-02 | 4.35E-01 | -2.54E-02 | 3.84E-01 | -3.06E-02 |
| 1618 | Alcohol usually taken with meals | 59781 | 4.90E-120 | -2.00E-01 | 9.06E-01 | -6.16E-03 | 8.92E-01 | -7.55E-03 |
| 20117 | Alcohol drinker status | 116063 | 3.94E-25 | -7.67E-02 | 1.27E-03 | -1.46E-01 | 1.73E-02 | -1.16E-01 |
| alc_combined | Average weekly alcohol intake | 81549 | 1.98E-48 | 2.33E-04 | 2.73E-01 | 1.03E-04 | 1.86E-01 | 1.32E-04 |
| 1259 | Smoking/smokers in household | 104657 | 4.11E-76 | 1.61E-01 | 2.15E-01 | 6.78E-02 | 7.53E-01 | 1.83E-02 |
| 1269 | Exposure to tobacco smoke at home | 102735 | 6.61E-37 | 8.51E-03 | 5.21E-01 | 2.53E-03 | 2.92E-01 | 4.51E-03 |
| 1279 | Exposure to tobacco smoke outside home | 96449 | 2.57E-73 | 2.50E-02 | 6.37E-01 | 3.98E-03 | 3.58E-01 | 8.38E-03 |
| 20116 | Smoking status | 115827 | 2.83E-18 | 3.68E-02 | 1.58E-04 | 9.73E-02 | 2.36E-03 | 8.36E-02 |
| 20160 | Ever smoked | 108058 | 6.17E-53 | 9.37E-02 | 3.86E-02 | 7.74E-02 | 1.26E-01 | 6.12E-02 |
| 20161 | Pack years of smoking | 38067 | 2.10E-74 | 5.50E-03 | 2.73E-02 | 3.70E-03 | 6.94E-02 | 3.25E-03 |
| 20162 | Pack years adult smoking as proportion of life span exposed to smoking | 38046 | 3.79E-70 | 2.38E-01 | 1.65E-02 | 1.79E-01 | 4.61E-02 | 1.58E-01 |

N: number of individuals included in the respective analyses. *E*: the results, with corresponding estimates (*β)* and p-values *(p)* for the linear models testing for the effect on each lifestyle variable on BMI without including the interaction term. *GS_BMI_ × E*: Results for the interaction term from linear models for association with the genetic score for BMI composed of the effects of 94 SNPs associated with BMI. *β2*: Estimated effect sizes of the interaction. *p2:* p-value for tests of the estimated effect size deviating from zero. *GS_BMI_' × E* is the genetic score for BMI excluding the *FTO* SNP rs1558902 with corresponding estimates (*β3)* and p-values *(p3)* for the interaction terms.
