# Supplementary material for: Gene-environment interaction study for BMI reveals interactions between genetic factors and physical activity, alcohol consumption and socioeconomic status
Source: PLoS Genet. 2017 Sep 5;13(9):e1006977. doi: 10.1371/journal.pgen.1006977 (PMC5600404; doi:10.1371/journal.pgen.1006977)
Supplement: S6 Table — N: number of individuals included in the respective analyses. E: the results, with corresponding estimates (β) and p-values (p) for the linear models testing for the effect on each lifestyle variable on BMI without including the interaction term. GSBMI × E: Results for the interaction term from linear models for association with the genetic score for BMI composed of the effects of 94 SNPs associated with BMI. β2: Estimated effect sizes of the interaction. p2: p-value for tests of the estimated effect size deviating from zero. GSBMI' × E is the genetic score for BMI excluding the FTO SNP rs1558902 with corresponding estimates (β3) and p-values (p3) for the interaction terms. (DOCX) [file pgen.1006977.s009.docx]

**S6 Table. Effect by, and interactions between genetic risk score for BMI and physical activity, assessed by self-report touchscreen questionnaire.**

| **ID** | **NAME** | **N** | ***E*** | | | ***GS_BMI_ × E*** | | | ***GS_BMI_' × E*** | |
| --- | --- | --- | --- | --- | --- | --- | --- | --- | --- | --- |
|  |  |  | ***p*** | ***β*** | | ***p2*** | ***β2*** | | ***p3*** | ***β3*** |
| 806 | Job involves mainly walking or standing | 65552 | 1.68E-05 | | 1.48E-02 | 5.50E-01 | -1.26E-02 | 6.86E-01 | | -9.11E-03 |
| 864 | Number of days/week walked 10+ minutes | 114174 | 1.35E-256 | | -5.16E-02 | 2.68E-05 | -3.92E-02 | 1.02E-04 | | -3.88E-02 |
| 874 | Duration of walks | 98543 | 1.00E-12 | | -3.40E-04 | 7.74E-01 | -8.40E-05 | 3.81E-01 | | -2.74E-04 |
| 884 | Number of days/week of moderate physical activity 10+ minutes | 110619 | 3.43E-290 | | -4.61E-02 | 1.46E-07 | -4.07E-02 | 1.65E-06 | | -3.96E-02 |
| 894 | Duration of moderate activity | 85751 | 5.84E-15 | | -3.95E-04 | 1.43E-01 | 4.52E-04 | 1.96E-01 | | 4.26E-04 |
| 904 | Number of days/week of vigorous physical activity 10+ minutes | 110534 | 1.80E-255 | | -5.22E-02 | 3.09E-04 | -3.38E-02 | 3.06E-04 | | -3.61E-02 |
| 914 | Duration of vigorous activity | 62644 | 2.22E-14 | | -7.68E-04 | 4.54E-02 | -1.25E-03 | 9.60E-02 | | -1.11E-03 |
| 924 | Usual walking pace | 115525 | <2.2E-308 | | -4.91E-01 | 1.10E-19 | -2.53E-01 | 1.04E-14 | | -2.31E-01 |
| 943 | Frequency of stair climbing in last 4 weeks | 115244 | <2.2E-308 | | -9.46E-02 | 3.67E-06 | -6.40E-02 | 2.34E-04 | | -5.44E-02 |
| 971 | Frequency of walking for pleasure in last 4 weeks | 82316 | 1.27E-10 | | -1.44E-02 | 6.63E-01 | -5.98E-03 | 7.97E-01 | | -3.78E-03 |
| 981 | Duration walking for pleasure | 82265 | 1.58E-182 | | -6.52E-02 | 2.27E-02 | -3.17E-02 | 1.11E-01 | | -2.37E-02 |
| 1011 | Frequency of light DIY in last 4 weeks | 58686 | 3.75E-20 | | -2.77E-02 | 5.75E-01 | 1.03E-02 | 5.88E-01 | | 1.07E-02 |
| 1021 | Duration of light DIY | 58386 | 7.03E-06 | | -1.20E-02 | 2.19E-01 | -2.01E-02 | 2.53E-01 | | -2.00E-02 |
| 1070 | Time spent watching television (TV) | 110003 | <2.2E-308 | | 1.27E-01 | 9.11E-07 | 5.96E-02 | 9.15E-06 | | 5.74E-02 |
| 1080 | Time spent using computer | 90683 | 1.34E-57 | | 4.10E-02 | 1.84E-01 | 2.08E-02 | 2.78E-01 | | 1.82E-02 |
| 1090 | Time spent driving | 77591 | 2.49E-105 | | 6.60E-02 | 2.46E-03 | -5.70E-02 | 1.17E-02 | | -5.05E-02 |
| 1110 | Length of mobile phone use | 114765 | 1.12E-138 | | 5.48E-02 | 7.19E-03 | -3.60E-02 | 2.05E-02 | | -3.32E-02 |
| 1120 | Weekly usage of mobile phone in last 3 months | 97310 | 1.59E-154 | | 6.67E-02 | 9.56E-01 | -8.61E-04 | 8.82E-01 | | -2.45E-03 |
| 2237 | Plays computer games | 116081 | <2.2E-308 | | 2.53E-01 | 1.47E-01 | 5.03E-02 | 1.56E-01 | | 5.27E-02 |
| 2624 | Frequency of heavy DIY in last 4 weeks | 47674 | 1.94E-02 | | -8.29E-03 | 8.46E-01 | 4.27E-03 | 7.20E-01 | | 8.36E-03 |
| 2634 | Duration of heavy DIY | 47752 | 8.33E-01 | | -5.32E-04 | 7.03E-01 | -5.91E-03 | 7.34E-01 | | -5.62E-03 |

N: number of individuals included in the respective analyses. *E*: the results, with corresponding estimates (*β)* and p-values *(p)* for the linear models testing for the effect on each lifestyle variable on BMI without including the interaction term. *GS_BMI_ × E*: Results for the interaction term from linear models for association with the genetic score for BMI composed of the effects of 94 SNPs associated with BMI. *β2*: Estimated effect sizes of the interaction. *p2:* p-value for tests of the estimated effect size deviating from zero. *GS_BMI_' × E* is the genetic score for BMI excluding the *FTO* SNP rs1558902 with corresponding estimates (*β3)* and p-values *(p3)* for the interaction terms.
