# Supplementary material for: Gene-environment interaction study for BMI reveals interactions between genetic factors and physical activity, alcohol consumption and socioeconomic status
Source: PLoS Genet. 2017 Sep 5;13(9):e1006977. doi: 10.1371/journal.pgen.1006977 (PMC5600404; doi:10.1371/journal.pgen.1006977)
Supplement: S7 Table — N: number of individuals included in the respective analyses. E: the results, with corresponding estimates (β) and p-values (p) for the linear models testing for the effect on each lifestyle variable on BMI without including the interaction term. GSBMI × E: Results for the interaction term from linear models for association with the genetic score for BMI composed of the effects of 94 SNPs associated with BMI. β2: Estimated effect sizes of the interaction. p2: p-value for tests of the estimated effect size deviating from zero. GSBMI' × E is the genetic score for BMI excluding the FTO SNP rs1558902 with corresponding estimates (β3) and p-values (p3) for the interaction terms. (DOCX) [file pgen.1006977.s010.docx]

**S7 Table. Effect by, and interactions between genetic risk score for BMI and socioeconomic factors. N: number of individuals included in the respective analyses.**

| **ID** | **NAME** | **N** | ***E*** | | ***GS_BMI_ × E*** | | ***GS_BMI_' × E*** | |
| --- | --- | --- | --- | --- | --- | --- | --- | --- |
|  |  |  | ***p*** | ***β*** | ***p2*** | ***β2*** | ***p3*** | ***β3*** |
| **189** | **Townsend deprivation index at recruitment** | **115988** | **1.32E-121** | **2.31E-02** | **2.38E-10** | **3.80E-02** | 3.57E-08 | 3.52E-02 |
| 680 | Own or rent accommodation lived in | 113679 | 8.24E-216 | 1.22E-01 | 1.34E-03 | 7.57E-02 | 2.32E-02 | 5.71E-02 |
| 699 | Length of time at current address | 112381 | 1.16E-01 | 4.41E-04 | 8.57E-01 | 3.11E-04 | 5.35E-01 | -1.14E-03 |
| **709** | **Number in household** | **115505** | **4.55E-05** | **-1.03E-02** | **1.65E-05** | **-6.71E-02** | 7.17E-05 | -6.81E-02 |
| **728** | **Number of vehicles in household** | **115444** | **9.39E-01** | **-2.62E-04** | **1.02E-06** | **-1.03E-01** | 4.29E-05 | -9.23E-02 |
| **738** | **Average total household income before tax** | **100421** | **7.50E-142** | **-7.21E-02** | **4.60E-07** | **-8.78E-02** | 5.85E-05 | -7.47E-02 |
| 757 | Time employed in main current job | 61669 | 3.80E-02 | -8.05E-04 | 1.46E-01 | 3.48E-03 | 1.85E-01 | 3.39E-03 |
| 767 | Length of working week for main job | 64213 | 6.50E-55 | 5.54E-03 | 3.98E-01 | 1.84E-03 | 6.57E-01 | 1.04E-03 |
| 777 | Frequency of travelling from home to job workplace | 64408 | 2.87E-23 | 1.96E-02 | 2.69E-01 | 1.41E-02 | 3.26E-01 | 1.30E-02 |
| 796 | Distance between home and job workplace | 55427 | 2.90E-04 | 1.69E-04 | 3.40E-01 | -3.63E-04 | 9.02E-01 | 4.87E-05 |
| 816 | Job involves heavy manual or physical work | 65557 | 4.51E-20 | 3.97E-02 | 9.23E-01 | -2.57E-03 | 8.34E-01 | 5.90E-03 |
| 826 | Job involves shift work | 65488 | 2.92E-61 | 7.47E-02 | 8.85E-03 | 7.30E-02 | 6.24E-03 | 8.12E-02 |
| 845 | Age completed full time education | 79238 | 2.98E-105 | -4.22E-02 | 2.84E-01 | -1.28E-02 | 6.33E-01 | -6.06E-03 |
| 2405 | Number of children fathered | 54292 | 1.39E-33 | 3.94E-02 | 5.92E-03 | -5.53E-02 | 1.31E-02 | -5.32E-02 |
| 4537 | Work/job satisfaction | 42456 | 1.29E-02 | 5.87E-03 | 3.39E-01 | 1.39E-02 | 4.83E-01 | 1.09E-02 |
| 4581 | Financial situation satisfaction | 42469 | 5.63E-94 | 1.05E-01 | 1.06E-03 | 1.03E-01 | 3.23E-02 | 7.15E-02 |
| 6138 | Qualifications | 94047 | 6.75E-39 | 2.44E-02 | 3.94E-01 | 9.82E-03 | 3.71E-01 | 1.10E-02 |

*E*: the results, with corresponding estimates (*β)* and p-values *(p)* for the linear models testing for the effect on each lifestyle variable on BMI without including the interaction term. *GS_BMI_ × E*: Results for the interaction term from linear models for association with the genetic score for BMI composed of the effects of 94 SNPs associated with BMI. *β2*: Estimated effect sizes of the interaction. *p2:* p-value for tests of the estimated effect size deviating from zero. *GS_BMI_' × E* is the genetic score for BMI excluding the *FTO* SNP rs1558902 with corresponding estimates (*β3)* and p-values *(p3)* for the interaction terms.
