# Supplementary material for: Gene-environment interaction study for BMI reveals interactions between genetic factors and physical activity, alcohol consumption and socioeconomic status
Source: PLoS Genet. 2017 Sep 5;13(9):e1006977. doi: 10.1371/journal.pgen.1006977 (PMC5600404; doi:10.1371/journal.pgen.1006977)
Supplement: S8 Table — N: number of individuals included in the respective analyses. E: the results, with corresponding estimates (β) and p-values (p) for the linear models testing for the effect on each lifestyle variable on BMI without including the interaction term. GSBMI × E: Results for the interaction term from linear models for association with the genetic score for BMI composed of the effects of 94 SNPs associated with BMI. β2: Estimated effect sizes of the interaction. p2: p-value for tests of the estimated effect size deviating from zero. GSBMI' × E is the genetic score for BMI excluding the FTO SNP rs1558902 with corresponding estimates (β3) and p-values (p3) for the interaction terms. (DOCX) [file pgen.1006977.s011.docx]

**S8 Table. Effect by, and interactions between genetic risk score for BMI and mental health factors. N: number of individuals included in the respective analyses.**

| **ID** | **NAME** | **N** | ***E*** | | ***GS_BMI_ × E*** | | ***GS_BMI_' × E*** | |
| --- | --- | --- | --- | --- | --- | --- | --- | --- |
|  |  |  | ***p*** | ***β*** | ***p2*** | ***β2*** | ***p3*** | ***β3*** |
| 1031 | Frequency of friend/family visits | 115609 | 3.82E-60 | -4.27E-02 | 8.66E-01 | -2.70E-03 | 9.47E-01 | -1.14E-03 |
| 1100 | Drive faster than motorway speed limit | 113038 | 1.12E-05 | 1.14E-02 | 9.06E-01 | 1.88E-03 | 7.91E-01 | 4.52E-03 |
| 1200 | Sleeplessness / insomnia | 116054 | 2.94E-79 | 7.69E-02 | 4.54E-01 | 1.88E-02 | 9.23E-01 | -2.59E-03 |
| 1920 | Mood swings | 113465 | 5.18E-117 | 1.37E-01 | 4.01E-02 | 7.52E-02 | 4.44E-02 | 7.86E-02 |
| 1930 | Miserableness | 114292 | 1.27E-85 | 1.18E-01 | 7.50E-03 | 9.89E-02 | 2.14E-02 | 9.08E-02 |
| 1940 | Irritability | 111219 | 3.29E-32 | 7.84E-02 | 7.71E-01 | -1.19E-02 | 5.75E-01 | -2.43E-02 |
| 1950 | Sensitivity / hurt feelings | 112994 | 1.18E-04 | 2.33E-02 | 4.23E-01 | 2.99E-02 | 5.28E-01 | 2.51E-02 |
| 1960 | Fed-up feelings | 113941 | 2.99E-203 | 1.83E-01 | 4.43E-07 | 1.86E-01 | 2.39E-06 | 1.86E-01 |
| 1970 | Nervous feelings | 113360 | 8.55E-154 | -1.85E-01 | 9.18E-01 | 4.39E-03 | 7.47E-01 | 1.47E-02 |
| 1980 | Worrier / anxious feelings | 113284 | 6.01E-59 | -9.73E-02 | 2.63E-01 | -4.13E-02 | 4.03E-01 | -3.30E-02 |
| 1990 | Tense / highly strung | 112784 | 2.51E-23 | -7.83E-02 | 6.88E-02 | 8.78E-02 | 9.15E-02 | 8.68E-02 |
| 2000 | Worry too long after embarrassment | 111553 | 3.26E-27 | -6.49E-02 | 5.62E-01 | -2.14E-02 | 5.76E-01 | -2.20E-02 |
| 2010 | Suffer from nerves | 112063 | 2.43E-13 | -5.31E-02 | 6.95E-01 | -1.75E-02 | 9.28E-01 | -4.31E-03 |
| 2020 | Loneliness, isolation | 114486 | 1.29E-96 | 1.60E-01 | 9.68E-03 | 1.22E-01 | 1.45E-02 | 1.22E-01 |
| 2030 | Guilty feelings | 113261 | 9.48E-01 | 4.29E-04 | 1.08E-01 | 6.52E-02 | 5.09E-02 | 8.45E-02 |
| 2040 | Risk taking | 112263 | 2.08E-22 | 6.73E-02 | 8.37E-01 | -8.75E-03 | 8.96E-01 | -5.90E-03 |
| 2050 | Frequency of depressed mood in last 2 weeks | 111366 | 1.49E-65 | 8.38E-02 | 1.14E-05 | 1.33E-01 | 3.59E-05 | 1.33E-01 |
| 2060 | Frequency of unenthusiasm / disinterest in last 2 weeks | 112483 | 3.60E-123 | 1.16E-01 | 1.73E-03 | 9.47E-02 | 3.97E-03 | 9.26E-02 |
| 2070 | Frequency of tenseness / restlessness in last 2 weeks | 112092 | 1.54E-21 | 4.65E-02 | 4.29E-02 | 6.08E-02 | 1.22E-01 | 4.94E-02 |
| 2090 | Seen doctor (GP) for nerves, anxiety, tension or depression | 115458 | 1.86E-67 | 1.08E-01 | 3.46E-01 | 3.59E-02 | 2.72E-01 | 4.47E-02 |
| 2100 | Seen a psychiatrist for nerves, anxiety, tension or depression | 115711 | 1.84E-18 | 8.03E-02 | 6.22E-01 | 2.78E-02 | 6.09E-01 | 3.07E-02 |
| 2110 | Able to confide | 112749 | 2.22E-11 | -1.05E-02 | 3.45E-01 | -9.14E-03 | 2.97E-01 | -1.08E-02 |
| 4526 | Happiness | 42497 | 1.02E-01 | 1.12E-02 | 5.06E-02 | 8.28E-02 | 1.22E-01 | 6.97E-02 |
| 4559 | Family relationship satisfaction | 42241 | 6.69E-01 | 2.30E-03 | 2.93E-01 | 3.52E-02 | 1.82E-01 | 4.75E-02 |
| 4570 | Friendships satisfaction | 42169 | 5.93E-02 | -1.24E-02 | 4.46E-01 | -3.12E-02 | 6.02E-01 | -2.28E-02 |
| 4598 | Ever depressed for a whole week | 41887 | 2.36E-11 | 6.56E-02 | 2.03E-02 | 1.41E-01 | 1.28E-02 | 1.61E-01 |
| 4609 | Longest period of depression | 17997 | 4.48E-03 | 1.02E-03 | 2.03E-01 | 2.86E-03 | 1.28E-01 | 3.73E-03 |
| 4620 | Number of depression episodes | 16216 | 7.31E-03 | 3.29E-03 | 3.44E-01 | 7.01E-03 | 1.03E-01 | 1.34E-02 |
| 4631 | Ever unenthusiastic/disinterested for a whole week | 41017 | 8.79E-22 | 9.82E-02 | 4.46E-02 | 1.27E-01 | 7.18E-02 | 1.21E-01 |
| 4642 | Ever manic/hyper for 2 days | 41671 | 8.09E-18 | 1.84E-01 | 9.74E-01 | -4.35E-03 | 7.84E-01 | -3.95E-02 |
| 4653 | Ever highly irritable/argumentative for 2 days | 41786 | 2.23E-73 | 2.33E-01 | 2.80E-01 | 8.61E-02 | 3.64E-01 | 7.71E-02 |
| 20126 | Bipolar and major depression status_recoded | 27320 | 1.08E-24 | 1.40E-01 | 3.48E-02 | 1.76E-01 | 1.23E-02 | 2.23E-01 |
| 20127 | Neuroticism score | 94717 | 5.96E-08 | 5.42E-03 | 3.73E-02 | 1.28E-02 | 3.18E-02 | 1.41E-02 |

*E*: the results, with corresponding estimates (*β)* and p-values *(p)* for the linear models testing for the effect on each lifestyle variable on BMI without including the interaction term. *GS_BMI_ × E*: Results for the interaction term from linear models for association with the genetic score for BMI composed of the effects of 94 SNPs associated with BMI. *β2*: Estimated effect sizes of the interaction. *p2:* p-value for tests of the estimated effect size deviating from zero. *GS_BMI_' × E* is the genetic score for BMI excluding the *FTO* SNP rs1558902 with corresponding estimates (*β3)* and p-values *(p3)* for the interaction terms.
