# Supplementary material for: Gene-environment interaction study for BMI reveals interactions between genetic factors and physical activity, alcohol consumption and socioeconomic status
Source: PLoS Genet. 2017 Sep 5;13(9):e1006977. doi: 10.1371/journal.pgen.1006977 (PMC5600404; doi:10.1371/journal.pgen.1006977)
Supplement: S9 Table — N: number of individuals included in the respective analyses. E: the results, with corresponding estimates (β) and p-values (p) for the linear models testing for the effect on each lifestyle variable on BMI without including the interaction term. GSBMI × E: Results for the interaction term from linear models for association with the genetic score for BMI composed of the effects of 94 SNPs associated with BMI. β2: Estimated effect sizes of the interaction. p2: p-value for tests of the estimated effect size deviating from zero. GSBMI' × E is the genetic score for BMI excluding the FTO SNP rs1558902 with corresponding estimates (β3) and p-values (p3) for the interaction terms. (DOCX) [file pgen.1006977.s012.docx]

**S9 Table. Effect by, and interactions between genetic risk score for BMI and general factors related to sleep, health and female-specific factors.**

| **ID** | **NAME** | **N** | ***E*** | | ***GS_BMI_ × E*** | | ***GS_BMI_' × E*** | |
| --- | --- | --- | --- | --- | --- | --- | --- | --- |
|  |  |  | ***p*** | ***β*** | ***p2*** | ***β2*** | ***p3*** | ***β3*** |
| 137 | Number of treatments/ medications taken | 116127 | <2.2E-308 | 8.17E-02 | 7.02E-05 | 2.63E-02 | 1.18E-03 | 2.29E-02 |
| 1160 | Sleep duration | 115531 | 1.10E-07 | -1.41E-02 | 5.40E-02 | 3.15E-02 | 2.85E-02 | 3.81E-02 |
| 1170 | Getting up in morning | 115997 | 7.13E-13 | -2.77E-02 | 3.17E-03 | -7.00E-02 | 1.76E-02 | -6.00E-02 |
| 1180 | Morning/evening person (chronotype) | 104054 | 1.32E-07 | 1.74E-02 | 1.61E-03 | 6.40E-02 | 7.40E-03 | 5.80E-02 |
| 1190 | Nap during day | 116098 | 4.14E-289 | 1.79E-01 | 3.96E-05 | 1.25E-01 | 1.76E-04 | 1.21E-01 |
| 1210 | Snoring | 108160 | <2.2E-308 | -4.05E-01 | 6.08E-01 | -1.95E-02 | 8.77E-01 | 6.29E-03 |
| 1220 | Daytime dozing / sleeping (narcolepsy) | 115753 | 8.97E-136 | 1.47E-01 | 4.45E-02 | 7.25E-02 | 2.34E-02 | 8.74E-02 |
| 2080 | Frequency of tiredness / lethargy in last 2 weeks | 112854 | <2.2E-308 | 1.50E-01 | 5.68E-07 | 1.09E-01 | 9.31E-06 | 1.03E-01 |
| 2375 | Relative age of first facial hair | 53278 | 1.93E-116 | -1.99E-01 | 9.61E-01 | 2.57E-03 | 9.58E-01 | -2.96E-03 |
| 2385 | Relative age voice broke | 50978 | 2.36E-96 | -2.55E-01 | 6.26E-01 | -3.63E-02 | 5.62E-01 | -4.59E-02 |
| 2714 | Age when periods started (menarche) | 59379 | <2.2E-308 | -1.03E-01 | 7.09E-01 | -6.22E-03 | 5.93E-01 | -9.53E-03 |
| 2724 | Had menopause | 51236 | 5.68E-01 | -1.08E-02 | 1.40E-02 | -2.86E-01 | 1.07E-02 | -3.19E-01 |
| 2734 | Number of live births | 61087 | 1.30E-22 | 3.85E-02 | 9.78E-05 | -9.41E-02 | 2.55E-04 | -9.41E-02 |
| 2744 | Birth weight of first child | 49043 | 1.71E-25 | 4.15E-02 | 7.45E-01 | 7.92E-03 | 7.74E-01 | -7.48E-03 |
| 2754 | Age at first live birth | 41577 | 3.00E-240 | -3.79E-02 | 8.08E-01 | -1.70E-03 | 7.13E-01 | 2.75E-03 |
| 2764 | Age at last live birth | 41518 | 5.94E-104 | -2.34E-02 | 9.91E-01 | 7.13E-05 | 5.94E-01 | 3.76E-03 |
| 2784 | Ever taken oral contraceptive pill | 61039 | 7.89E-07 | -5.96E-02 | 1.30E-02 | -1.85E-01 | 1.42E-02 | -1.94E-01 |
| 2794 | Age started oral contraceptive pill | 48756 | 1.36E-08 | -7.48E-03 | 4.43E-01 | 6.23E-03 | 5.10E-01 | 5.71E-03 |
| 2804 | Age when last used oral contraceptive pill | 44305 | 3.20E-23 | -6.79E-03 | 5.51E-03 | -1.17E-02 | 2.26E-03 | -1.38E-02 |
| 2814 | Ever used hormone-replacement therapy (HRT) | 60981 | 4.02E-07 | 4.95E-02 | 8.29E-01 | -1.29E-02 | 6.72E-01 | -2.72E-02 |
| 3536 | Age started hormone-replacement therapy (HRT) | 22023 | 3.14E-49 | -2.00E-02 | 7.09E-01 | -3.11E-03 | 6.37E-01 | -4.21E-03 |
| 4080 | Systolic blood pressure, automated reading | 109298 | <2.2E-308 | 9.20E-03 | 5.86E-01 | -5.58E-04 | 2.87E-01 | -1.16E-03 |
| 20022 | Birth weight | 66992 | 9.19E-12 | 4.01E-02 | 1.99E-01 | -4.61E-02 | 2.57E-01 | -4.35E-02 |
| 24024 | Average 24-hour sound level of noise pollution | 114563 | 1.47E-03 | 2.21E-03 | 7.81E-01 | -1.18E-03 | 6.61E-01 | 1.99E-03 |

N: number of individuals included in the respective analyses. *E*: the results, with corresponding estimates (*β)* and p-values *(p)* for the linear models testing for the effect on each lifestyle variable on BMI without including the interaction term. *GS_BMI_ × E*: Results for the interaction term from linear models for association with the genetic score for BMI composed of the effects of 94 SNPs associated with BMI. *β2*: Estimated effect sizes of the interaction. *p2:* p-value for tests of the estimated effect size deviating from zero. *GS_BMI_' × E* is the genetic score for BMI excluding the *FTO* SNP rs1558902 with corresponding estimates (*β3)* and p-values *(p3)* for the interaction terms.
