# Supplementary material for: Gene-environment interaction study for BMI reveals interactions between genetic factors and physical activity, alcohol consumption and socioeconomic status
Source: PLoS Genet. 2017 Sep 5;13(9):e1006977. doi: 10.1371/journal.pgen.1006977 (PMC5600404; doi:10.1371/journal.pgen.1006977)
Supplement: S10 Table — Results for interactions between GSBMI and environmental factors are shown. Results for the full model are available in the supplementary Data. Stepwise linear regression was performed as was described in the methods section. Age, age2 and sex were included as covariates. Interaction terms for all secondary interactions were included to control for potential confounding. The model also included a batch variable for the two genotyping platforms used in the UK Biobank, as well as 15 principal components. p-values < 0.05 were considered significant. (DOCX) [file pgen.1006977.s013.docx]

**S10 Table. Results from the final model generated by the stepwise regression model.**

| **Factor** | ***β*** | ***p*** |
| --- | --- | --- |
| *GS_BMI_* × 1558: Frequency of alcohol intake | 5.77E-02 | 2.82E-15 |
| *GS_BMI_* × 924: Usual walking pace | -1.42E-01 | 1.55E-14 |
| *GS_BMI_* × 864: Number of days/week walked 10+ minutes | -1.92E-02 | 6.26E-04 |
| *GS_BMI_* × 20116: Smoking status | 5.09E-02 | 1.45E-03 |
| *GS_BMI_* × 904: Number of days/week of vigorous physical activity 10+ minutes | -1.70E-02 | 2.28E-03 |
| *GS_BMI_* × 728: Number of vehicles in household | -3.37E-02 | 1.03E-02 |
| *GS_BMI_* × 189: Townsend deprivation index | 8.49E-03 | 3.02E-02 |
| *GS_BMI_* × 2080: Frequency of tiredness / lethargy in last 2 weeks | 2.79E-02 | 3.87E-02 |
| *GS_BMI_* × 943: Frequency of stair climbing in last 4 weeks | -1.56E-02 | 5.86E-02 |
| *GS_BMI_* × 1190: Nap during day | 2.69E-02 | 1.35E-01 |

Results for interactions between *GS_BMI_* and environmental factors are shown. Results for the full model are available in the supplementary Data. Stepwise linear regression was performed as was described in the methods section. Age, age^2^ and sex were included as covariates. Interaction terms for all secondary interactions were included to control for potential confounding. The model also included a batch variable for the two genotyping platforms used in the UK Biobank, as well as 15 principal components. *p*-values < 0.05 were considered significant.
