# Supplementary material for: Gene-environment interaction study for BMI reveals interactions between genetic factors and physical activity, alcohol consumption and socioeconomic status
Source: PLoS Genet. 2017 Sep 5;13(9):e1006977. doi: 10.1371/journal.pgen.1006977 (PMC5600404; doi:10.1371/journal.pgen.1006977)
Supplement: S11 Table — N: number of individuals included in the respective analyses. β1–3: Estimated effect sizes of the interaction terms. p1-3: p-value for tests of the estimated effect size deviating from zero. (DOCX) [file pgen.1006977.s014.docx]

**S11 Table. Interactions between environmental factors and *GS_BMI_* when Townsend deprivation index (TDI) is included as a covariate in linear regression models, and when the genetic score is composed of effect estimates generated in UK Biobank (*GS_BMI__UKBB*).**

| **ID** | **name** | **N** | ***GS_BMI_*** *×* ***E*** | | | ***GS_BMI_*** *×* ***E***  **(TDI included as covariate)** | | | ***GS_BMI__UKBB*** × ***E*** | | |
| --- | --- | --- | --- | --- | --- | --- | --- | --- | --- | --- | --- |
|  |  |  | ***p1*** | | ***β1*** | ***p2*** | | ***β2*** | ***p3*** | | ***β3*** |
| 924 | Usual walking pace | 115,525 | 1.10E-19 | -2.53E-01 | | 4.81E-17 | -2.36E-01 | | 1.48E-21 | -3.09E-01 | |
| 1558 | Alcohol intake frequency. | 116,063 | 1.87E-16 | 9.92E-02 | | 8.03E-14 | 9.05E-02 | | 6.78E-18 | 1.21E-01 | |
| 189 | Townsend deprivation index at recruitment | 115,988 | 2.38E-10 | 3.80E-02 | | 2.03E-10 | 3.81E-02 | | 1.76E-10 | 4.43E-02 | |
| 884 | Number of days/week of moderate physical activity 10+ minutes | 110,619 | 1.46E-07 | -4.07E-02 | | 1.13E-07 | -4.09E-02 | | 5.51E-08 | -4.87E-02 | |
| 1960 | Fed-up feelings | 113,941 | 4.43E-07 | 1.86E-01 | | 9.33E-06 | 1.64E-01 | | 3.67E-07 | 2.18E-01 | |
| 738 | Average total household income before tax | 100,421 | 4.60E-07 | -8.78E-02 | | 5.74E-04 | -6.25E-02 | | 7.28E-07 | -1.00E-01 | |
| 2080 | Frequency of tiredness / lethargy in last 2 weeks | 112,854 | 5.68E-07 | 1.09E-01 | | 1.09E-05 | 9.61E-02 | | 1.08E-06 | 1.23E-01 | |
| 1070 | Time spent watching television (TV) | 110,003 | 9.11E-07 | 5.96E-02 | | 2.98E-05 | 5.09E-02 | | 2.62E-06 | 6.61E-02 | |
| 728 | Number of vehicles in household | 115,444 | 1.02E-06 | -1.03E-01 | | 3.60E-03 | -6.56E-02 | | 7.46E-06 | -1.09E-01 | |
| 943 | Frequency of stair climbing in last 4 weeks | 115,244 | 3.67E-06 | -6.40E-02 | | 1.51E-04 | -5.26E-02 | | 1.64E-06 | -7.67E-02 | |
| 2050 | Frequency of depressed mood in last 2 weeks | 111,366 | 1.14E-05 | 1.33E-01 | | 9.26E-05 | 1.19E-01 | | 4.26E-05 | 1.44E-01 | |
| 709 | Number in household | 115,505 | 1.65E-05 | -6.71E-02 | | 9.35E-03 | -4.12E-02 | | 5.21E-04 | -6.38E-02 | |
| 864 | Number of days/week walked 10+ minutes | 114,174 | 2.68E-05 | -3.92E-02 | | 4.03E-06 | -4.30E-02 | | 2.58E-05 | -4.54E-02 | |
| 1190 | Nap during day | 116,098 | 3.96E-05 | 1.25E-01 | | 2.99E-04 | 1.10E-01 | | 7.32E-06 | 1.57E-01 | |
| 137 | Number of treatments/ medications taken | 116,127 | 7.02E-05 | 2.63E-02 | | 9.98E-04 | 2.19E-02 | | 1.99E-05 | 3.28E-02 | |
| 2734 | Number of live births | 61,087 | 9.78E-05 | -9.41E-02 | | 1.33E-04 | -9.19E-02 | | 6.57E-05 | -1.12E-01 | |
| 20116 | Smoking status | 115,827 | 1.58E-04 | 9.73E-02 | | 1.08E-02 | 6.67E-02 | | 6.17E-05 | 1.20E-01 | |
| 1568 | Average weekly red wine intake | 81,566 | 2.37E-04 | -1.58E-02 | | 6.52E-04 | -1.47E-02 | | 1.21E-04 | -1.92E-02 | |
| 904 | Number of days/week of vigorous physical activity 10+ minutes | 110,534 | 3.09E-04 | -3.38E-02 | | 5.37E-04 | -3.24E-02 | | 3.16E-04 | -3.93E-02 | |
| 4581 | Financial situation satisfaction | 42,469 | 1.06E-03 | 1.03E-01 | | 6.44E-03 | 8.64E-02 | | 9.58E-03 | 9.39E-02 | |
| 20117 | Alcohol drinker status | 116,063 | 1.27E-03 | -1.46E-01 | | 5.86E-03 | -1.24E-01 | | 3.68E-04 | -1.87E-01 | |
| 680 | Own or rent accommodation lived in | 113,679 | 1.34E-03 | 7.57E-02 | | 3.09E-01 | 2.61E-02 | | 1.28E-03 | 8.82E-02 | |
| 1180 | Morning/evening person (chronotype) | 104,054 | 1.61E-03 | 6.40E-02 | | 3.07E-03 | 5.99E-02 | | 1.48E-03 | 7.48E-02 | |
| 2060 | Frequency of unenthusiasm / disinterest in last 2 weeks | 112,483 | 1.73E-03 | 9.47E-02 | | 1.40E-02 | 7.47E-02 | | 3.24E-03 | 1.04E-01 | |
| 1548 | Variation in diet | 115,853 | 2.13E-03 | 9.17E-02 | | 3.62E-03 | 8.67E-02 | | 1.10E-02 | 8.81E-02 | |
| 1090 | Time spent driving | 77,591 | 2.46E-03 | -5.70E-02 | | 2.19E-02 | -4.35E-02 | | 8.25E-03 | -5.74E-02 | |
| 1349 | Processed meat intake | 115,966 | 3.11E-03 | 5.22E-02 | | 3.80E-03 | 5.10E-02 | | 2.78E-03 | 6.12E-02 | |
| 1170 | Getting up in morning | 115,997 | 3.17E-03 | -7.00E-02 | | 6.43E-03 | -6.45E-02 | | 7.24E-03 | -7.39E-02 | |
| 1299 | Salad / raw vegetable intake | 108,682 | 4.50E-03 | -3.11E-02 | | 3.68E-03 | -3.17E-02 | | 4.02E-03 | -3.65E-02 | |
| 2804 | Age when last used oral contraceptive pill | 44,305 | 5.51E-03 | -1.17E-02 | | 9.82E-03 | -1.09E-02 | | 3.20E-03 | -1.44E-02 | |
| 2405 | Number of children fathered | 54,292 | 5.92E-03 | -5.53E-02 | | 1.75E-02 | -4.79E-02 | | 4.78E-03 | -6.59E-02 | |
| 1110 | Length of mobile phone use | 114,765 | 7.19E-03 | -3.60E-02 | | 1.56E-02 | -3.24E-02 | | 2.79E-03 | -4.66E-02 | |
| 1930 | Miserableness | 114,292 | 7.50E-03 | 9.89E-02 | | 2.89E-02 | 8.09E-02 | | 1.89E-02 | 1.01E-01 | |
| 826 | Job involves shift work | 65,488 | 8.85E-03 | 7.30E-02 | | 2.38E-02 | 6.33E-02 | | 3.50E-02 | 6.86E-02 | |
| 1438 | Bread intake | 112,695 | 9.60E-03 | 6.35E-03 | | 2.15E-02 | 5.62E-03 | | 4.26E-03 | 8.12E-03 | |
| 2020 | Loneliness. isolation | 114,486 | 9.68E-03 | 1.22E-01 | | 6.62E-02 | 8.70E-02 | | 2.84E-02 | 1.20E-01 | |
| 2784 | Ever taken oral contraceptive pill | 61,039 | 1.30E-02 | -1.85E-01 | | 1.77E-02 | -1.76E-01 | | 3.59E-02 | -1.81E-01 | |

N: number of individuals included in the respective analyses. *β1-3*: Estimated effect sizes of the interaction terms. *p1-3:* p-value for tests of the estimated effect size deviating from zero.
