# Supplementary material for: Gene-environment interaction study for BMI reveals interactions between genetic factors and physical activity, alcohol consumption and socioeconomic status
Source: PLoS Genet. 2017 Sep 5;13(9):e1006977. doi: 10.1371/journal.pgen.1006977 (PMC5600404; doi:10.1371/journal.pgen.1006977)
Supplement: S12 Table — Values in the bottom diagonal represent Kendall tau coefficients (τ). Values in the upper diagonal represent p-values. (DOCX) [file pgen.1006977.s015.docx]

**S12 Table.** **Results from Kendall rank correlation tests between the 19 environmental variables that were observed to interact with GSBMI.**

| ***τ \ p*** | **Name** | **1558** | **1568** | **20116** | **864** | **884** | **904** | **924** | **943** | **1070** | **189** | **709** | **728** | **738** | **1960** | **2050** | **137** | **1190** | **2080** | **2734** |
| --- | --- | --- | --- | --- | --- | --- | --- | --- | --- | --- | --- | --- | --- | --- | --- | --- | --- | --- | --- | --- |
| **1558** | Alcohol intake |  | 2.2*  10^-308^ | 9.0*  10^-223^ | 0.95 | 0.19 | 1.2*  10^-64^ | 2.2*  10^-308^ | 1.0*  10^-99^ | 2.2*  10^-308^ | 2.2*  10^-308^ | 4.1*  10^-28^ | 2.2*  10^-308^ | 2.2*  10^-308^ | 2.2*  10^-308^ | 2.2*  10^-308^ | 2.2*  10^-308^ | 2.2*  10^-308^ | 2.2*  10^-308^ | 2.2*  10^-308^ |
| **1568** | Red wine | -0.29 |  | 6.6*  10^-4^ | 1.8*  10^-4^ | 9.5*  10^-4^ | 2.2*  10^-308^ | 2.2*  10^-308^ | 2.2*  10^-308^ | 2.4*  10^-204^ | 8.9*  10^-190^ | 2.2*  10^-308^ | 2.2*  10^-308^ | 2.2*  10^-308^ | 6.7*  10^-118^ | 1.1*  10^-38^ | 4.42E-30 | 7.2*  10^-32^ | 3.0*  10^-68^ | 1.9*  10^-15^ |
| **20116** | Smoking | -0.08 | -0.01 |  | 0.88 | 9.2*  10^-2^ | 7.9*  10^-54^ | 1.0*  10^-267^ | 1.6*  10^-176^ | 2.2*  10^-308^ | 2.2*  10^-308^ | 1.4*  10^-165^ | 2.7*  10^-263^ | 2.2*  10^-308^ | 2.2*  10^-308^ | 2.2*  10^-308^ | 2.2*  10^-308^ | 2.2*  10^-308^ | 2.2*  10^-308^ | 5.1*  10^-2^ |
| **864** | Walking | 0.00 | -0.01 | 0.00 |  | 2.2*  10^-308^ | 2.2*  10^-308^ | 2.2*  10^-308^ | 2.2*  10^-308^ | 6.1*  10^-24^ | 2.2*  10^-308^ | 5.8*  10^-41^ | 4.9*  10^-198^ | 7.6*  10^-148^ | 1.7*  10^-42^ | 2.2*  10^-34^ | 8.8*  10^-16^ | 2.2*  10^-4^ | 7.5*  10^-183^ | 1.9*  10^-12^ |
| **884** | Moderate phys. | 0.00 | -0.01 | 0.00 | 0.31 |  | 2.2*  10^-308^ | 2.2*  10^-308^ | 2.2*  10^-308^ | 9.2*  10^-34^ | 2.2*  10^-308^ | 1.3*  10^-7^ | 2.1*  10^-27^ | 3.6*  10^-193^ | 1.9*  10^-55^ | 6.5*  10^-34^ | 2.5*  10^-32^ | 0.99 | 2.4*  10^-176^ | 2.2*  10^-308^ |
| **904** | vigorous phys. | -0.04 | 0.03 | -0.04 | 0.18 | 0.40 |  | 2.2*  10^-308^ | 2.2*  10^-308^ | 2.6*  10^-261^ | 7.6*  10^-21^ | 2.2*  10^-308^ | 2.2*  10^-308^ | 2.2*  10^-308^ | 1.3*  10^-104^ | 6.9*  10^-79^ | 2.2*  10^-308^ | 1.2*  10^-40^ | 2.2*  10^-308^ | 2.7*  10^-2^ |
| **924** | Walking pace | -0.10 | 0.09 | -0.10 | 0.10 | 0.09 | 0.19 |  | 2.2*  10^-308^ | 2.2*  10^-308^ | 5.5*  10^-215^ | 2.2*  10^-308^ | 2.2*  10^-308^ | 2.2*  10^-308^ | 2.2*  10^-308^ | 5.2*  10^-169^ | 2.2*  10^-308^ | 2.2*  10^-308^ | 2.2*  10^-308^ | 2.0*  10^-25^ |
| **943** | Stairs | -0.05 | 0.05 | -0.07 | 0.08 | 0.09 | 0.09 | 0.15 |  | 4.9*  10^-278^ | 1.7*  10^-201^ | 2.2*  10^-308^ | 2.2*  10^-308^ | 2.2*  10^-308^ | 5.5*  10^-68^ | 3.8*  10^-43^ | 3.9*  10^-206^ | 2.4*  10^-72^ | 4.8*  10^-116^ | 2.2*  10^-308^ |
| **1070** | TV | 0.07 | -0.09 | 0.09 | -0.02 | -0.03 | -0.08 | -0.17 | -0.09 |  | 2.2*  10^-308^ | 2.2*  10^-308^ | 2.2*  10^-308^ | 2.2*  10^-308^ | 2.2*  10^-308^ | 2.2*  10^-308^ | 2.2*  10^-308^ | 2.2*  10^-308^ | 2.2*  10^-308^ | 2.2*  10^-308^ |
| **189** | TDI | 0.07 | -0.07 | 0.13 | 0.04 | 0.02 | -0.02 | -0.07 | -0.07 | 0.06 |  | 2.2*  10^-308^ | 2.2*  10^-308^ | 2.2*  10^-308^ | 2.2*  10^-308^ | 2.2*  10^-308^ | 2.2*  10^-308^ | 2.2*  10^-308^ | 2.2*  10^-308^ | 1.9*  10^-20^ |
| **709** | household n. | -0.03 | 0.02 | -0.07 | -0.03 | -0.01 | 0.04 | 0.07 | 0.14 | -0.11 | -0.11 |  | 2.2*  10^-308^ | 2.2*  10^-308^ | 3.9*  10^-4^ | 4.4*  10^-10^ | 2.2*  10^-308^ | 1.0*  10^-197^ | 1.0*  10^-4^ | 2.2*  10^-308^ |
| **728** | vehicles n. | -0.10 | 0.08 | -0.09 | -0.08 | -0.03 | 0.04 | 0.08 | 0.09 | -0.11 | -0.25 | 0.43 |  | 2.2*  10^-308^ | 1.6*  10^-105^ | 7.1*  10^-160^ | 2.2*  10^-308^ | 1.1*  10^-203^ | 2.2*  10^-89^ | 2.2*  10^-308^ |
| **738** | income | -0.17 | 0.14 | -0.12 | -0.07 | -0.08 | 0.06 | 0.19 | 0.11 | -0.24 | -0.17 | 0.36 | 0.41 |  | 4.5*  10^-216^ | 1.1*  10^-177^ | 2.2*  10^-308^ | 2.2*  10^-308^ | 5.1*  10^-109^ | 1.3*  10^-16^ |
| **1960** | Fed-up | 0.07 | -0.07 | 0.09 | -0.04 | -0.04 | -0.06 | -0.11 | -0.05 | 0.10 | 0.09 | 0.01 | -0.06 | -0.09 |  | 2.2*  10^-308^ | 2.2*  10^-308^ | 2.2*  10^-308^ | 2.2*  10^-308^ | 0.5 |
| **2050** | depressed | 0.05 | -0.04 | 0.07 | -0.03 | -0.03 | -0.05 | -0.08 | -0.04 | 0.04 | 0.08 | -0.02 | -0.07 | -0.08 | 0.46 |  | 2.2*  10^-308^ | 2.2*  10^-308^ | 2.2*  10^-308^ | 4.2*  10^-4^ |
| **137** | treatments | 0.08 | -0.03 | 0.07 | -0.02 | -0.03 | -0.09 | -0.20 | -0.07 | 0.15 | 0.05 | -0.13 | -0.11 | -0.19 | 0.09 | 0.09 |  | 2.2*  10^-308^ | 2.2*  10^-308^ | 3.9*  10^-10^ |
| **1190** | Naps | 0.04 | -0.04 | 0.08 | -0.01 | 0.00 | -0.04 | -0.13 | -0.05 | 0.13 | 0.05 | -0.08 | -0.08 | -0.14 | 0.07 | 0.05 | 0.14 |  | 2.2*  10^-308^ | 4.4*  10^-5^ |
| **2080** | tired/leth. | 0.09 | -0.05 | 0.07 | -0.07 | -0.07 | -0.11 | -0.16 | -0.06 | 0.05 | 0.08 | 0.01 | -0.05 | -0.06 | 0.35 | 0.38 | 0.15 | 0.13 |  | 1.5*  10^-2^ |
| **2734** | births | 0.04 | -0.03 | 0.01 | 0.02 | 0.04 | -0.01 | -0.04 | 0.04 | 0.03 | -0.03 | 0.25 | 0.11 | -0.03 | 0.00 | -0.01 | 0.02 | 0.01 | -0.01 |  |

Values in the bottom diagonal represent Kendall tau coefficients (*τ*). Values in the upper diagonal represent p-values.
