# Supplementary material for: Gene-environment interaction study for BMI reveals interactions between genetic factors and physical activity, alcohol consumption and socioeconomic status
Source: PLoS Genet. 2017 Sep 5;13(9):e1006977. doi: 10.1371/journal.pgen.1006977 (PMC5600404; doi:10.1371/journal.pgen.1006977)
Supplement: S1 Supporting Information — (DOCX) [file pgen.1006977.s017.docx]

# S1 Supporting Information - Power calculations

**Methods**

Power calculations were performed by simulating genotype and BMI data for all the 94 SNPs published by GIANT that were in HWE in UK biobank. For each SNP, the minor allele frequency (MAF) and effect size was taken from Locke et al. The sample size in the sample size was chosen to represent the UK biobank sample used in our study (N= 116,000)

## Association between SNPs or genetic score with BMI in UK biobank

A total of 1000 simulations were performed. The power is expressed as the fraction of times the P-value for the association between the SNP and BMI was lower than 0.05/94 SNPs tested = 5.32*10^-4^. For *GS_BMI_* the power is expressed as the fraction of times the P-value for the association between *GS_BMI_* and BMI was lower than 0.05.

## Effect of the interaction between individual SNPs and alcohol intake frequency on BMI

For simulating the interaction with alcohol consumption, the sample sizes for each alcohol category was taken from the UK biobank data (S1 Table). We assumed that the genetic effect was twice as high in the non-consumers compared to the most highly consuming group with a dose-dependent linear relation between alcohol categories. For individual SNPs the effect size was rescaled so that the average effect across alcohol intake categories were equal to one. This was done by multiplying the effect by 0.73, 0.87, 1.02, 1.16, 1.31,and 1.45 respectively for the group drinking daily or almost daily, three or four times a week, once or twice a week, one to three times a month, at special occasions only, and never. Here, 1000 replicates were performed for each SNP and the power for individual SNP was expressed as the number of times the P-value was lower than 0.05/131 lifestyle factors tested/94 SNPs tested =4.06 * 10^-6^.

## Effect of the interaction between GS_BMI_ and alcohol intake frequency on BMI

We calculated interaction scores for the 94 SNPs together using the rescaled effect sizes for the individual SNPs. Such simulations for the genetic score assume that the genetic effects for all SNPs are influenced in the same direction by alcohol consumption (the genetic effect is twice the effect in non-drinkers compared to frequent drinkers). To test how the power is influenced if only a subset of the SNPs are interacting with alcohol intake frequency, we repeated the simulations by assuming that only N SNPs interacted with alcohol intake frequency, and that the other (94-N) did not interact. A total of 1000 replicates were produced with N ranging from 0 to 94. To test how the power is influenced if a subset of the SNPs have negative interacting effects (the effect is larger in frequent drinkers compared to non-drinkers), we also simulated data where the effect was positive for N and negative for 94-N SNPs. A total of 1000 replicates were produced with N ranging from 47 to 94 where N=47 corresponds to that 50% of the SNPs have positive and 50% negative effects, and N=94 that all SNPs have positive effects.

# Results

## Association between SNPs or genetic score with BMI in UK biobank

The power to detect an association with BMI for individual SNPs are shown in the table below (Table 1). The ten most significant SNPs all had a power of 100% to be detected in UK biobank. Among the 31 SNPs that did not replicate in UK biobank, as many as 22 had a power below 80% to be detected in the cohort. The power to detect the association between BMI and the genetic score in UK biobank was, not surprisingly, estimated to 100%

**Table 1. The 94 SNPs for which we attempt to replicate the association with BMI.**

| **RSID** | ***p*** | ***p*-adj** | **power**  **(adj 94 test)** |
| --- | --- | --- | --- |
| rs1558902 | 4.49*10^-89^ | 4.22*10^-87^ | 1.00 |
| rs13021737 | 1.27*10^-29^ | 1.19*10^-27^ | 1.00 |
| rs543874 | 3.25*10^-29^ | 3.05*10^-27^ | 1.00 |
| rs6567160 | 3.99*10^-29^ | 3.75*10^-27^ | 1.00 |
| rs10182181 | 7.37*10^-19^ | 6.93*10^-17^ | 1.00 |
| rs3817334 | 2.71*10^-16^ | 2.55*10^-14^ | 1.00 |
| rs10938397 | 1.13*10^-14^ | 1.06*10^-12^ | 1.00 |
| rs11030104 | 3.20*10^-14^ | 3.01*10^-12^ | 1.00 |
| rs7138803 | 3.32*10^-14^ | 3.12*10^-12^ | 1.00 |
| rs2207139 | 1.01*10^-13^ | 9.54*10^-12^ | 1.00 |
| rs3101336 | 5.32*10^-13^ | 5.00*10^-11^ | 1.00 |
| rs2287019 | 1.14*10^-12^ | 1.07*10^-10^ | 1.00 |
| rs2112347 | 1.43*10^-12^ | 1.35*10^-10^ | 1.00 |
| rs16951275 | 3.76*10^-12^ | 3.53*10^-10^ | 1.00 |
| rs3888190 | 3.99*10^-12^ | 3.75*10^-10^ | 1.00 |
| rs2365389 | 6.40*10^-12^ | 6.01*10^-10^ | 0.90 |
| rs205262 | 1.39*10^-11^ | 1.30*10^- 9^ | 0.82 |
| rs3810291 | 5.40*10^-11^ | 5.07*10^-9^ | 1.00 |
| rs1808579 | 7.16*10^-11^ | 6.73*10^-9^ | 0.63 |
| rs13107325 | 4.81*10^-10^ | 4.52*10^-8^ | 0.99 |
| rs12446632 | 1.18*10^-9^ | 1.11*10^-7^ | 1.00 |
| rs2176598 | 4.26*10^-9^ | 4.00*10^-7^ | 0.76 |
| rs1516725 | 4.43*10^-9^ | 4.17*10^-7^ | 1.00 |
| rs12429545 | 5.09*10^-9^ | 4.78*10^-7^ | 0.95 |
| rs4256980 | 6.67*10^-9^ | 6.27*10^-7^ | 0.92 |
| rs7903146 | 1.76*10^-8^ | 1.66*10^-6^ | 0.94 |
| rs6091540 | 1.82*10^-8^ | 1.71*10^-6^ | 0.81 |
| rs10968576 | 3.14*10^-8^ | 2.95*10^-6^ | 0.98 |
| rs7164727 | 3.76*10^-8^ | 3.53*10^-6^ | 0.78 |
| rs1167827 | 4.16*10^-8^ | 3.91*10^-6^ | 0.91 |
| rs17724992 | 2.18*10^-7^ | 2.05*10^-5^ | 0.78 |
| rs17024393 | 2.53*10^-7^ | 2.38*10^-5^ | 0.99 |
| rs751414 | 5.24*10^-7^ | 4.93*10^-5^ | 0.72 |
| rs1016287 | 9.75*10^-7^ | 9.17*10^-5^ | 0.95 |
| rs4787491 | 9.89*10^-7^ | 9.30*10^-5^ | 0.61 |
| rs11583200 | 1.08*10^-6^ | 1.01*10^-4^ | 0.76 |
| rs11165643 | 1.25*10^-6^ | 1.17*10^-4^ | 0.97 |
| rs2820292 | 1.77*10^-6^ | 1.66*10^-4^ | 0.78 |
| rs1528435 | 2.59*10^-6^ | 2.43*10^-4^ | 0.83 |
| rs12940622 | 2.93*10^-6^ | 2.76*10^-4^ | 0.71 |
| rs4740619 | 3.04*10^-6^ | 2.86*10^-4^ | 0.69 |
| rs10132280 | 3.29*10^-6^ | 3.09*10^-4^ | 0.96 |
| rs13078960 | 5.49*10^-6^ | 5.16*10^-4^ | 0.98 |
| rs29941 | 5.63*10^-6^ | 5.29*10^-4^ | 0.73 |
| rs7599312 | 5.95*10^-6^ | 5.59*10^-4^ | 0.83 |
| rs2650492 | 7.00*10^-6^ | 6.58*10^-4^ | 0.87 |
| rs11191560 | 8.59*10^-6^ | 8.07*10^-4^ | 0.76 |
| rs11057405 | 1.28*10^-5^ | 1.20*10^-3^ | 0.76 |
| rs12566985 | 1.86*10^-5^ | 1.75*10^-3^ | 1.00 |
| rs10733682 | 1.91*10^-5^ | 1.80*10^-3^ | 0.85 |
| rs7141420 | 2.34*10^-5^ | 2.20*10^-3^ | 0.98 |
| rs13191362 | 2.85*10^-5^ | 2.68*10^-3^ | 0.88 |
| rs2245368 | 3.60*10^-5^ | 3.38*10^-3^ | 0.95 |
| rs977747 | 3.90*10^-5^ | 3.67*10^-3^ | 0.71 |
| rs2836754 | 4.20*10^-5^ | 3.95*10^-3^ | 0.68 |
| rs12885454 | 4.53*10^-5^ | 4.26*10^-3^ | 0.79 |
| rs9400239 | 4.87*10^-5^ | 4.57*10^-3^ | 0.65 |
| rs657452 | 7.18*10^-5^ | 6.75*10^-3^ | 0.98 |
| rs1441264 | 7.30*10^-5^ | 6.86*10^-3^ | 0.56 |
| rs12401738 | 7.64*10^-5^ | 7.19*10^-3^ | 0.88 |
| rs16851483 | 1.23*10^-4^ | 1.15*10^-2^ | 0.98 |
| rs7243357 | 4.75*10^-4^ | 4.46*10^-2^ | 0.75 |
| rs17405819 | 5.22*10^-4^ | 4.91*10^-2^ | 0.90 |
| rs16907751 | 8.57*10^-4^ | 8.06*10^-2^ | 0.87 |
| rs7239883 | 1.16*10^-3^ | 1.09*10^-1^ | 0.51 |
| rs11688816 | 1.21*10^-3^ | 0.11 | 0.52 |
| rs7715256 | 1.35*10^-3^ | 0.13 | 0.71 |
| rs1460676 | 1.66*10^-3^ | 0.16 | 0.72 |
| rs17094222 | 1.69*10^-3^ | 0.16 | 0.91 |
| rs7899106 | 1.78*10^-3^ | 0.17 | 0.78 |
| rs6804842 | 2.70*10^-3^ | 0.25 | 0.73 |
| rs2080454 | 4.24*10^-3^ | 0.40 | 0.74 |
| rs3849570 | 4.61*10^-3^ | 0.43 | 0.79 |
| rs758747 | 6.63*10^-3^ | 0.62 | 0.91 |
| rs9540493 | 7.26*10^-3^ | 0.68 | 0.79 |
| rs3736485 | 7.35*10^-3^ | 0.69 | 0.70 |
| rs9581854 | 7.38*10^-3^ | 0.69 | 0.99 |
| rs17203016 | 8.33*10^-3^ | 0.78 | 0.77 |
| rs12286929 | 1.45*10^-2^ | 1.36 | 0.97 |
| rs1928295 | 1.56*10^-2^ | 1.47 | 0.82 |
| rs9374842 | 2.38*10^-2^ | 2.24 | 0.77 |
| rs6477694 | 3.15*10^-2^ | 2.96 | 0.74 |
| rs1000940 | 4.02*10^-2^ | 3.78 | 0.66 |
| rs11847697 | 5.13*10^-2^ | 4.82 | 0.97 |
| rs9641123 | 7.53*10^-2^ | 7.08 | 0.78 |
| rs9914578 | 9.91*10^-2^ | 9.32 | 0.66 |
| rs13201877 | 0.18 | 1.72 | 0.62 |
| rs2176040 | 0.29 | 2.76 | 0.49 |
| rs2121279 | 0.31 | 2.88 | 0.72 |
| rs492400 | 0.46 | 4.32 | 0.50 |
| rs11126666 | 0.52 | 4.87 | 0.82 |
| rs2033732 | 0.58 | 5.49 | 0.59 |
| rs6465468 | 0.70 | 6.57 | 0.56 |
| rs11727676 | 0.83 | 7.83 | 0.94 |

*p* is the p-value for the association in UK biobank. *p*-adj is adjusted fro the 94 tests performed. power (adj 94 test) is the power to detect an association in UK biobank with a *p*-adj < 0.05.

## Effect of the interaction between individual SNPs and alcohol intake frequency on BMI

The power to detect an interaction between BMI and any of the individual SNPs was very limited with values below 5% for all SNP except the FTO variant (rs1558902) with a power estimate of 38%.

## Effect of the interaction between GS_BMI_ and alcohol intake frequency on BMI

The simulations with a subset of the SNPs interacting with alcohol intake frequency showed that already with 37 interacting SNPs, the power to detect the association is above 80% (Fig 1). The power to detect the association between BMI and the genetic score, if effect of alcohol consumption on the genetic effects were in opposite direction for some sites, dropt below 80% when less than 72% of the SNPs had an interaction effect that was in the same direction (Fig 2)


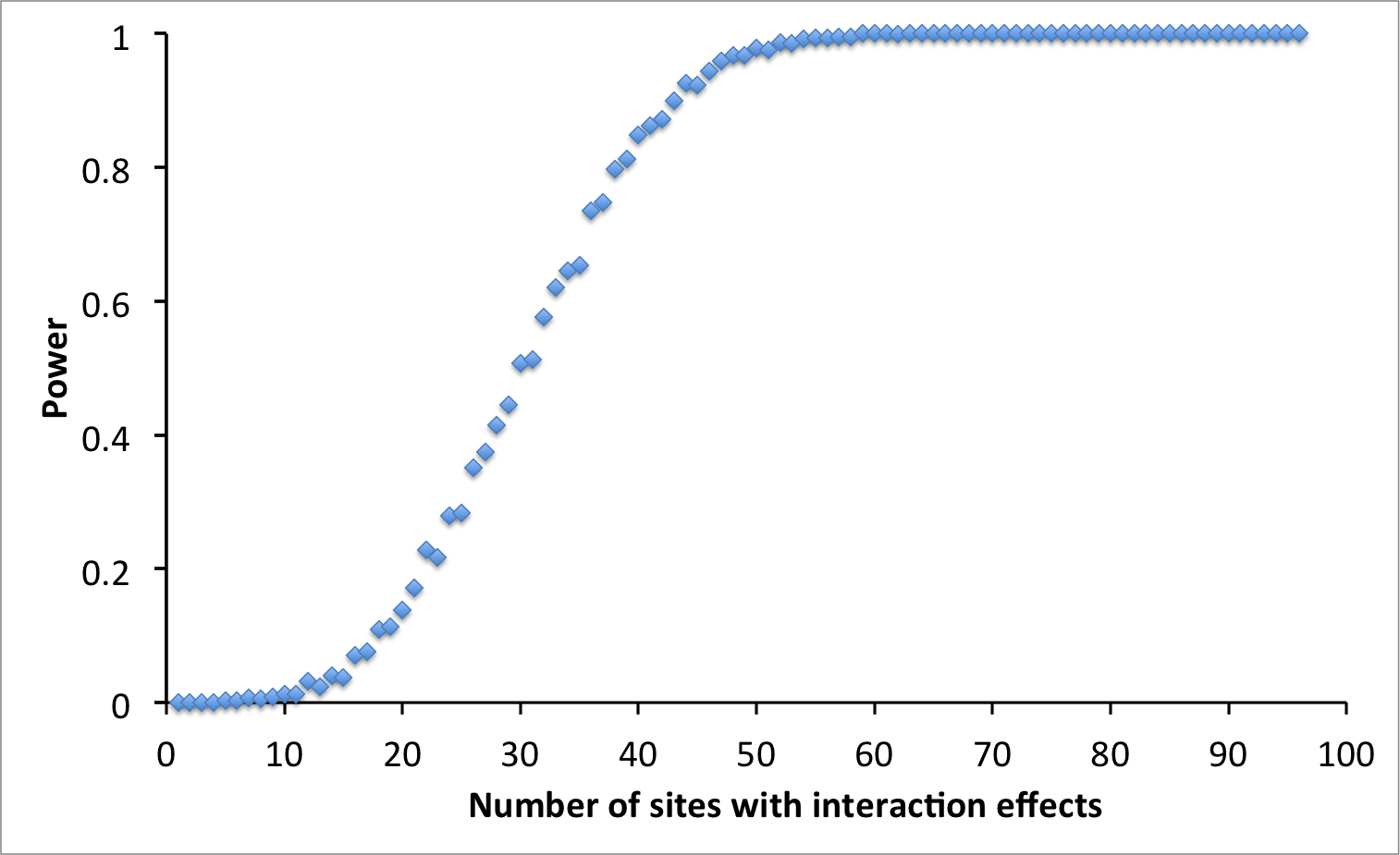


**Fig 1**. Power to detect an interaction depending on how many of the SNPs having interaction effects.


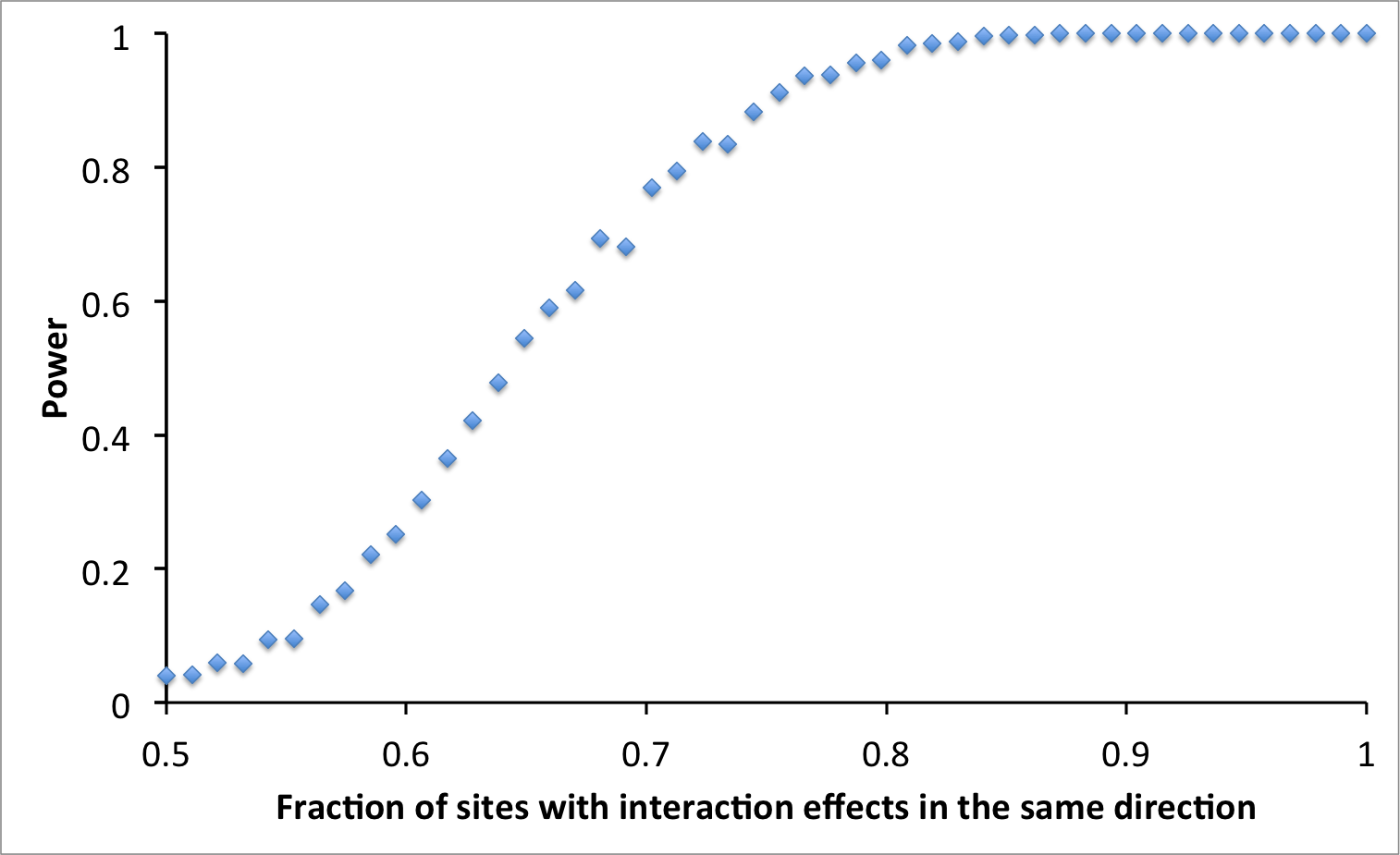


**Fig 2.**  Power to detect an interaction depending on what fraction of the SNPs having interaction in the same direction. A fraction of 0.5 means that alcohol consumption increases the genetic effect for 50% of the SNPs and decreases the genetic effect for 50% of the SNPs.
