# Supplementary material for: Gene-environment interaction study for BMI reveals interactions between genetic factors and physical activity, alcohol consumption and socioeconomic status
Source: PLoS Genet. 2017 Sep 5;13(9):e1006977. doi: 10.1371/journal.pgen.1006977 (PMC5600404; doi:10.1371/journal.pgen.1006977)
Supplement: S2 Supporting Information — (DOCX) [file pgen.1006977.s018.docx]

# S2 Supporting Information - Validation of alcohol and physical activity data

## Method

To test the validity of measurements related to physical activity and alcohol consumption, we performed logistic regression modeling to assess the statistical significance of the association between the self-report questionnaire data and the complementary, 24-hour recall questionnaire data. For this purpose, we were able to utilize data from the entire cohort. We included participants who self reported as being white and having British ancestry. 24-hour recall data was available for 211,050 individuals also answered the question: “Did you have any alcoholic drinks yesterday?” For physical activity, 24-hour recall data was available for: time spent doing vigorous physical activity (N = 211,048), moderate physical activity (N = 211,048) and light physical activity (N = 211,048). The definition of light physical activities includes, but is not restricted to, walks. Nevertheless, we make use of this variable to validate the frequency/duration of walks.

Frequency of alcohol intake was coded in a reversed order: “1” corresponds to “Daily or almost daily”, “2” to “Three or four times a week”, “3” to “Once or twice a week”, 4 to “One or three times a month”, “5” to “Special occasions only” and “6” to “Never”. Frequency of alcohol intake was modeled against “Did you have any alcohol drinks yesterday?” (yes/no) in the 24-hour questionnaire. Physical activity was assessed in the 24-hour recall data by multiple choice questions in the format: “Yesterday, about how long did you spend doing activities that needed moderate effort, making you somewhat short of breath?”. Participants were given the options to reply “None”, “Under 10 minutes”, “10-30 minutes”, “½-1hour”, “1-2 hours”, “2-4 hours”, “4-6 hours” or “6+ hours”. Light activity was assessed by the question: “Yesterday, about how long did you spend doing activities that needed some light effort, involving movement but not making you short of breath”, with the options to answer: “None”, “Under 1 hour”, “1-3 hours”, “3-5 hours”, “5-7 hours”, “7-9 hours”, “9-12 hours”, “12+ hours”.

The 24-hour recall data for duration of physical activity was recoded to follow an increasing sequence $0,1,\ldots,m-1$ where *m* denotes the number of categories of the variable in question. Hence, category “0” denotes the 24-hour-recall response with the shortest duration, i.e. “Under 10 minutes”, while category “$m-1$” denotes the response with the longest duration. To be able to model the self-report questionnaire data on frequency of physical activity against 24-hour recall data using logistic regression, we dichotomized the durations reported in the 24-hour recall data. Individuals who answered that they spent $\geq10$ min on physical activities yesterday were considered to have spent time on physical activities (coded as “1”), while those individuals who spent $<10$ min of physical activities were not (coded as “0”).

Self-report data on frequency of more than 10 minutes of walking was tested for correlation with 24-hour recall data on time spent doing light physical activity. 24-hour recall data on light physical activity was recoded as a dichotomous variable. Participants who reported no light physical activity were set as “0” and participants who reported 1-3 hours or more were set as “1”. The self-report data on frequency of walking had a lower cut-off of ten minutes, while the lowest category for physical activity in the 24-hour recall data was “Under one hour”. Because of this difference, we set a random sample of 5/6 of the participants who reported to have performed < 1h of light physical activity as ‘1’, to represent having performed more than 10 minutes of light physical activity, and the remaining 1/6 as ‘0’, to represent having performed less than 10 minutes of light physical activity.

The frequency data were modeled using simple logistic regression where the independent predictor variable *X* can either denote the self-reported frequency of alcohol consumption, or the number of days in a typical week doing physical activities $\geq10$ minutes, and let the corresponding binary output response variable *Y* = “Consumed alcohol” or “spent time doing physical activities yesterday”, coded as “yes” = 1 and “no” = 0. Then, we may assume that $Y_{i}\sim Bernoulli(p_{i})$ for each individual *i = 1,…,N*, where ${p_{i}=p}_{i}\left( x_{i} \right)\mathbb{= P(}Y_{i}=1|x_{i})$. By taking the logit-transformation of this conditional probability and assume that the transformed variable can be linearly modeled, we have that:

$\text{logit}\left( p_{i} \right):=\ln\left( \frac{p_{i}}{1-p_{i}} \right)=\gamma_{0}+\gamma_{1}\cdot x_{i}, i=1,\ldots,N.$ (3)

Logistic regression was performed in R using the glm function included in the stats-package.

The duration of physical activity was modeled using ordinal logistic regression (i.e., proportional odds model), where the response data *Y_i_* have *m* ordered categories. In this case, the logit-transformation is performed on the cumulative probabilities $\pi_{i,j}(x_{i}\mathbb{)= P(}Y_{i}\leq j|x_{i})$, such that:

$\text{logit}\text{(}\pi_{i,j}\text{)}:=\ln\left( \frac{\pi_{i,j}}{1-\pi_{i,j}} \right)=\ln\left( \frac{\mathbb{P(}Y_{i}\leq j|x_{i})}{\mathbb{P(}Y_{i}>j|x_{i})} \right)=\gamma_{0,j}-\gamma_{1}\cdot x_{i},$ (4)

where *i = 1,…,N* and *j = 0,…,m-2* since $\pi_{i,m-1}\mathbb{=P}\left( Y_{i}\leq m-1 | x_{i} \right)=1$ for all *x_i_* and needs not to be modeled. Note that the log-odds ratio $\gamma_{1}$is independent of *j*, while the intercepts $\gamma_{0,j}$ obey the inequality $\gamma_{0,j}< \gamma_{0,j´}$ for $j<j$´*.* The ordinal logistic regression modeling was performed in R using the polr command, which is included in the MASS library. The *z*-test was used to test for deviance of the slope of *γ_1_* from zero, see equation (3) and (4). P-values correspond to results from *z*-tests for deviance of the slope of *γ_1_* from zero.

**Results**

Logistic regression analyses reveal that self-reported touchscreen questionnaire data on physical activity and alcohol consumption predicted the responses to the 24-hour recall questionnaire. For alcohol intake frequency, a lower probability for having consumed alcohol yesterday was observed for each increase in alcohol consumption frequency category (OR = 0.354, *p* < 2.2 * 10^-308^) (Fig 1). An increase in response-category corresponds to a decrease in alcohol intake frequency, as this variable was coded in a reversed order (see the method section above).

**Fig 1: Higher frequency of self-reported alcohol intake frequency increases the probability of having consumed alcohol within the last 24 hours.** Frequency of alcohol intake was coded in a reversed order: “1” corresponds to “Daily or almost daily”, “2” to “Three or four times a week”, “3” to “Once or twice a week”, 4 to “One or three times a month”, “5” to “Special occasions only” and “6” to “Never”. The black, connected dots denote UK Biobank data while the small, red dots are the predictions from a logistic model fitted to the data. A highly significant association is observed between self-reported data and 24-hour recall (OR = 0.354, p < 2.2*10^-308^).

Higher frequency of self-reported physical activity was associated with an increased probability of physical activity in the last 24 hours. This was observed for light, moderate and vigorous physical activity; OR = 1.15, 1.21, 1.41 respectively, all p < 4.5 * 10^-308^. Ordinal logistic regression modeling also revealed self-reported durations of weekly physical activity to be associated with longer duration of physical activity in the last 24 hours; OR = 1.34, 1.33 and 1.93 for light, moderate and vigorous activity, respectively; all p-values < 2.2*10^-308^. For example, for vigorous physical activity, an increase in the self-reported duration is associated with a higher probability of reporting a higher amount in the 24-hour questionnaire (Fig 2A). Ordinal logistic regression modeling allows us to estimate the increased odds for reporting a higher category in the 24-hour recall data, as the number of hours in the self-report data increases. In our model, the odds of reporting a higher category in the 24-hour recall increases by a factor of 1.93 per one-hour increase in self-reported duration of average daily vigorous physical activity (Fig 2B).

**Fig 2:** **Duration of vigorous physical activity within the last 24 hours was associated with self-reported hours of daily vigorous physical activity.** (A): Each curve corresponds to a specific 24-hour recall category, denoted *j* (see box legend), and describes the probability that the duration of vigorous physical activity within the last 24 hours was reported to be higher than that of category *j* (*y*-axes), given the participant’s self-reported daily duration of vigorous physical activities in hours (*x*-axes). A clear ordering between the categories is observed, which is indicative of an association between the self-report-, and 24-hour recall data on vigorous physical activity. (B): The ordinal logistic regression model fitted to the vigorous physical activity data. This model shows, that an increase in the duration of self-reported activity in hours increases the probability of reporting a higher category of duration of activity in the 24-hour recall data.
